# Design of a multi-epitope vaccine against *Mycobacterium tuberculosis* using reverse vaccinology and immunoreactive peptides

**DOI:** 10.1186/s44342-026-00075-6

**Published:** 2026-07-08

**Authors:** Narjes Noori Goodarzi, Sepideh Fereshteh, Behzad Shahbazi, Niloofar Rezaie, Mahshid Khazani Asforooshani, Farzad Badmasti

**Affiliations:** 1 Department of Bacteriology, Pasteur Institute of Iran, Tehran, Iran; 2 Department of Pathobiology, School of Public Health, Tehran University of Medical Sciences, Tehran, Iran; 3 School of Pharmacy, Semnan University of Medical Sciences, Semnan, Iran; 4 Department of Microbiology, Faculty of Biological Sciences, Alzahra University, Tehran, Iran

**Keywords:** Tuberculosis, *Mycobacterium tuberculosis*, Mucosal immunity, Multi-epitope vaccine, Reverse vaccinology

## Abstract

**Background:**

*Mycobacterium tuberculosis* infects one-fourth of the global population, and current challenges such as latent infections, multidrug-resistant strains, and the limited efficacy of the BCG vaccine emphasize the urgent need for next-generation vaccines. This study aimed to introduce novel vaccine candidates, immunoreactive epitopes, and a novel multi-epitope vaccine (MEV).

**Methods:**

New immunogenic targets were identified based on different characteristics, including subcellular localization, antigenicity, non-similarity to the host proteome, sequence conservation, prevalence, and B-cell and T-cell epitopes. In the next step, IFN-γ releasing immunoreactive epitopes with a high similarity to TCR-interacting epitopes were identified. The MEV was generated using shortlisted epitopes and the C-terminal fragment of *Clostridium perfringens* enterotoxin (CPE). Finally, the interactions of MEV epitopes with human MHC I and MHC II alleles were investigated.

**Results:**

In the first step, a total of seven proteins with desired immunogenic properties were introduced as novel immunogenic targets. Comparison of surface-exposed proteins to 4718 immunoreactive linear B-cell epitopes of *M. tuberculosis* resulted in identification of 719 non-redundant immunoreactive epitopes. Finally, seven immunoreactive, IFN-γ releasing epitopes with significant homology to TCR binding epitopes were employed to design a MEV. This MEV showed desirable structural and immunogenic properties. Moreover, it revealed promising interactions with human MHC I and MHC II alleles in molecular docking.

**Conclusion:**

This study suggests PE/PPE proteins and TCR-recognized immunoreactive peptides as promising vaccine components against tuberculosis. In addition, the designed MEV with the C-terminal fragment of CPE may represent a potential candidate for future development as a mucosal vaccine against *M. tuberculosis*.

**Supplementary Information:**

The online version contains supplementary material available at 10.1186/s44342-026-00075-6.

## Introduction

Through a long co-evolutionary history with humans, *Mycobacterium tuberculosis* has become a highly successful pathogen, infecting 23–32% of the world's population. Currently, tuberculosis has become one of the leading infectious killers [1]. According to the 2024 World Health Organization (WHO) Global tuberculosis report, in 2023, the number of newly diagnosed tuberculosis (TB) cases worldwide reached 8.2 million, up from previous reports in the past five years. The recent increase in diagnosed TB cases is likely due to a backlog of individuals who developed the disease in earlier years but experienced delays in diagnosis and treatment as a result of disruptions caused by the COVID-19 pandemic. The global reduction in TB mortality from 2015 to 2023 was 23%, which falls significantly below the “WHO End TB Strategy” goal of a 75% reduction by 2025 [2].

Tuberculosis control strategies are primarily aimed at minimizing transmission through prompt detection and treatment of infectious cases. Controlling disease development in the infected population will be necessary to move forward to eliminate tuberculosis by 2050 [3].

Tuberculosis is an airborne disease, transmitted through the inhalation of droplet nuclei containing *M. tuberculosis*. These droplets travel through the oral or nasal cavities, pass through the upper respiratory tract and bronchi, and subsequently reach the pulmonary alveoli [4].

Exposure to *M. tuberculosis* can result in different outcomes from elimination of pathogen, to latent, subclinical and active TB disease. Any alteration in host immunity or comorbidities can lead to either progression or reversion of the condition. The pathogen can be eliminated by innate immune responses or acquired T-cell mediated immunity. If the pathogen is not eliminated, bacteria persist in a latent state. Patients with active TB disease exhibit symptoms such as cough, fever and weight loss [5].

Some symptomatic and active TB patients, such as those with smear-negative pulmonary TB or extrapulmonary TB, are considered non-infectious and pose a low risk of transmission [6, 7]. Certain active TB patients, which do not experience any symptoms despite their positive-culture results, are classified as subclinical TB patient [6]. However, patients with subclinical TB disease, either non-infectious or infectious, can transmit the pathogen to others [7].

On the other hand, antimicrobial resistance is an increasingly serious threat to global public health. Multidrug-resistant (MDR) *M. tuberculosis* strains, which are resistant to both major first-line tuberculosis drugs (*e.g.* rifampicin and isoniazid), account for approximately a quarter of all deaths caused by antibiotic-resistant infections [8]. Unfortunately, extensively drug-resistant (XDR) strains have arisen after the mis-management of individuals harboring MDR-TB [9].

To fulfill “End TB” globally, there is a pivotal need to develop effective vaccines that can disrupt the transmission cycle [10]. Bacille Calmette-Guérin (BCG) remains the only approved TB vaccine and is widely administered to neonates in most countries as part of the expanded WHO immunization program. While BCG vaccination in infants is moderately effective, it has shown controversial efficacy in preventing tuberculosis in adolescents and adults in several clinical trials and global epidemics [11, 12]. However, a recombinant BCG (VPM1002) has been developed that expresses a listeriolysin gene that assists with internalization to cell cytoplasm, and deletion of the urease enables the bacilli to escape the macrophage lysosome. The rBCG was less reactogenic, but its immunogenicity was not inferior to BCG in phase II clinical trial [13].

Nowadays, efforts are continuing on four major vaccine platforms: whole-organism vaccines (*e.g.* live-attenuated or inactivated), protein subunit vaccines, viral-vectored, and nucleic acid vaccines [14]. Because of the difficulties in identifying antigens capable of eliciting a protective response, whole-cell-derived vaccines have gained more attention. However, there are concerns that whole-cell vaccines may simply induce a semi-effective immune response similar to that seen in natural *M. tuberculosis* infection [15].

The stumbling block for the development of an effective tuberculosis vaccine is the inadequate comprehension of *M. tuberculosis* pathogenesis and protective approaches. The elimination of this pathogen involves both innate and adaptive immune responses. Antigen presenting cells (APCs) such as macrophages and dendritic cells (DCs) play a significant role through phagocytosis of *M. tuberculosis*. T-cells recognize *M. tuberculosis* peptides presented on the surface of DCs via major histocompatibility complex (MHC) molecules [16, 17]. Since the interaction between APCs and T-cells is based on peptide recognition rather than recognition of the full-length protein, identification of immunogenic epitopes is the key strategy to develop a novel effective vaccine against tuberculosis. Multi-epitope vaccines (MEVs) are a combination of immunodominant epitopes that aggregate the immunogenic features of different proteins and lower the side effects [18].

The Immune Epitope Database and Analysis Resource (IEDB) provides a vast depository of immunoreactive epitopes that are curated from published literature. Applying this collection to immunoinformatic studies can facilitate the development of protective vaccines and therapeutics [19]. Therefore, apart from the identification of novel vaccine candidates, this study aimed to utilize experimentally validated immunoreactive peptides to develop a MEV against *M. tuberculosis.* Furthermore, considering that the combination of antigens with novel adjuvants might provide protective and long-lasting immunity against TB, we aimed to use a novel adjuvant that can trigger mucosal immunity against tuberculosis.

## Methods

**Fig. 1:**
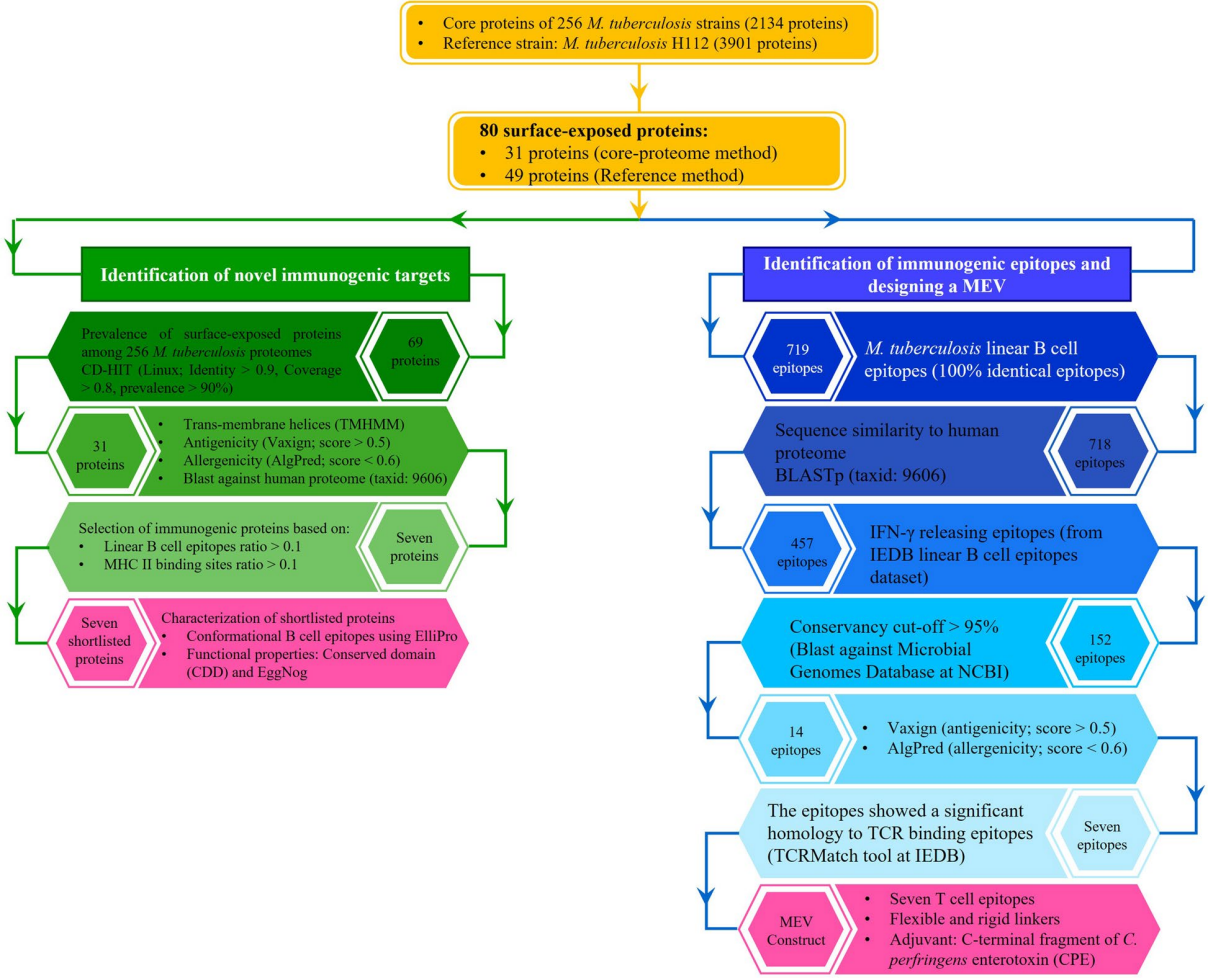
The workflow for the identification of novel immunogenic targets and immunodominant epitopes against *M. tuberculosis*. A total of seven immunogenic proteins and seven immunogenic epitopes were identified through immunoinformatic approaches. The immunogenic epitopes were utilized to develop a MEV against this pathogen

The workflow of the present study is illustrated in Fig. 1. The study consists of two distinct parts. The first strategy focuses on the investigation of immunogenic targets, whereas the second one deals involves the design of a multi-epitope vaccine (MEV) based on selected immunoreactive epitopes.

### Protein content analysis of M. tuberculosis strains

The protein content of 256 *M. tuberculosis* strains with complete whole genome sequences were downloaded from GenBank database and analyzed using BPGA software as a pan-genome analysis pipeline [20]. Core-proteins were retrieved with a cut-off = 0.8, and the core-pan plot was generated based on BPGA analysis.

### Identification of promising immunogenic targets

#### Identification of highly prevalent proteins

To identify the most promising immunogenic proteins, two approaches were followed. In the first method, core proteins were included, and on the second method, *M. tuberculosis* strain H112 (NZ_CP019613) was selected as the reference strain, and all its coding sequences (CDSs) were included. *M. tuberculosis* H112 is a hyper-virulent strain from the Beijing lineage. It also shows faster growth and improved survival within macrophages, which contributes to its heightened virulence [21]. The surface-exposed proteins were identified using PSORTb v3.0 (https://www.psort.org/psortb/) [22]. On the next step, the most prevalent surface-exposed proteins were identified through the CD-HIT tool with identity > 0.9 and coverage > 0.8. Each protein with a prevalence > 90% was selected.

#### Evaluating the immunogenic proteins

The number of transmembrane helices was determined using TMHMM (https://services.healthtech.dtu.dk/services/TMHMM-2.0/) [23]. The antigenicity of proteins was determined using the VaxiJen web server (http://www.ddg-pharmfac.net/vaxijen/VaxiJen/VaxiJen.html) with a cut-off ≥ 0.5 [24]. Then, the allergenicity was determined using AlgPred [25]. Next, surface-exposed proteins were compared to proteins of *Homo sapiens* (taxid: 9606) using BLASTp in the NCBI database (https://blast.ncbi.nlm.nih.gov/Blast.cgi?PAGE=Protein) [26]. Finally, the surface-exposed, antigenic, and non-allergen proteins with no similarity to the human proteome were selected for further analyses.

#### Identification of the linear B-cell epitope ratio and MHC II binding site ratio

To select the immunoreactive vaccine candidates, the number of linear B-cell epitopes and MHC II binding sites were determined using the BepiPred 3.0 (https://services.healthtech.dtu.dk/services/BepiPred-2.0/) [27] and the TepiTool (http://tools.iedb.org/tepitool/) [28], respectively. Then, the linear B-cell epitope ratio was calculated by dividing the number of linear B-cell epitopes by the total number of amino acids of each protein. Similarly, the MHC II binding sites ratio of each protein was calculated as well. Finally, proteins with ratios > 0.1 were selected for further investigation.

#### Prediction of conformational B-cell epitopes

The tertiary structures of the shortlisted proteins were predicted using the I-TASSER web tool (https://zhanggroup.org/I-TASSER/) [29]. After validation of predicted tertiary structures using ProSA-web (https://prosa.services.came.sbg.ac.at/prosa.php) [30], conformational B-cell epitopes were identified using the Ellipro web server available at (http://tools.iedb.org/ellipro/) [31] with a cut-off > 0.8. Next, the conservancy of shortlisted proteins was assessed using the ConSurf web server (https://consurf.tau.ac.il/) [32]. Finally, the function of proteins was investigated using the Conserved Domain Database (CDD) at NCBI (https://www.ncbi.nlm.nih.gov/Structure/cdd/cdd.shtml) [33] and EggNOG (http://eggnog5.embl.de/#/app/home) [34].

### Identification of immunogenic peptides

#### Identification of the immuno-reactive epitopes of M. tuberculosis

The immunoreactive epitopes of *M. tuberculosis* were retrieved from IEDB at (https://www.iedb.org/). The surface-exposed core proteins of *M. tuberculosis* strains were screened against the collection of immunoreactive epitopes of IEDB using BLAST, and epitopes with 100% identity and coverage were selected. Then, the redundant epitopes were excluded using Jalview [35].

#### Assessment of homology to the human proteome

The immuno-reactive epitopes were compared to proteins of *Homo sapiens* (taxid: 9606) using BLASTp in the NCBI database (https://blast.ncbi.nlm.nih.gov/Blast.cgi?PAGE=Protein). Proteins with any similarity to the host proteome were excluded.

#### Identification of IFN-γ releasing epitopes

The selected epitopes were compared with epitopes that are involved in B-cell, T-cell and MHC binding assays at IEDB database (https://www.iedb.org/). Then, the IFN-γ releasing epitopes were selected for further evaluation [36].

#### Conservancy status of IFN-γ releasing epitopes

To assess epitope conservancy, the 80 surface-exposed proteins of *M. tuberculosis* were compared against the Microbial Nucleotide blast database of NCBI available at (https://blast.ncbi.nlm.nih.gov/Blast.cgi?PAGE_TYPE=BlastSearch&BLAST_SPEC=MicrobialGenomes) [37]. Homologous proteins showing > 70% sequence identity and query coverage were retrieved and aligned using MegaX [38]. The resulting multiple sequence alignments (MSAs) were then used to evaluate the conservation of the epitopes using the IEDB conservancy database (http://tools.iedb.org/conservancy/) [39].

#### Evaluation of antigenicity and allergenicity of epitopes

The antigenicity of epitopes was determined using the VaxiJen web server (http://www.ddg-pharmfac.net/vaxijen/VaxiJen/VaxiJen.html) with a cut-off ≥ 1 [24]. Then, the allergenic epitopes were excluded using the AllerTOP v. 2.0 web tool (https://www.ddg-pharmfac.net/AllerTOP/) [40].

#### Sequence similarity to TCR binding epitopes

To select the most immunogenic epitopes, we compared the epitopes that were selected based on the above-mentioned criteria to a dataset of TCR epitopes available at the TCRMatch tool in the IEDB database (http://tools.iedb.org/tcrmatch/). Epitopes having TCR interactions with coverage > 50% and identity > 50% were selected.

#### Development of a multi-epitope vaccine

Epitopes with high sequence similarity to the TCR epitopes were selected and joined together using GPGPG flexible linkers. Then, to target the mucosal immunogenicity of the MEV, the C-terminal fragment of *Clostridium perfringens* toxin (CPE) was fused to the C-terminal of the MEV using a EAAAK rigid linker. This fragment lacks cytotoxicity but is capable of inducing mucosal immune responses [41].

#### Evaluation of the designed MEV

First, second and third structural properties of the designed MEV were evaluated. After predicting the antigenicity and allergenicity of the MEV, its molecular weight, theoretical pI, instability and aliphatic indices, and hydropathicity were predicted using the ProtParam web server (https://web.expasy.org/protparam/) [42]. Then, the solubility and toxicity of the construct was identified using the Protein-Sol (https://protein-sol.manchester.ac.uk/) [43] and the ToxinPred2 servers (https://webs.iiitd.edu.in/raghava/toxinpred2/) [44]. The secondary structure features, including disordered regions, alpha helices, and beta sheets, were predicted using the Phyre2 database (http://www.sbg.bio.ic.ac.uk/~phyre2/html/page.cgi?id=index) [45].

#### Identification of MHC-I and MHC-II epitopes of the MEV and Molecular Docking

The tertiary structure of the MEV was predicted using the I-TASSER database and validated using the ProSA-web tool. The MHC-I binding epitopes of the MEV were identified using NetCTL-1.2 (https://services.healthtech.dtu.dk/services/NetCTL-1.2/) with a threshold of 0.7. Moreover, the MHC-II binding sites were predicted using IEDB web tool (https://tools.iedb.org/mhcii/) with top 2% percentile rank. Epitopes showing the highest score were selected for molecular docking. The molecular interactions of MEV with HLA-B39 (PDB: 9C6V) and HLA-DRB1*01:01 (PDB: 5V4N) were investigated using the HDOCK web server (http://hdock.phys.hust.edu.cn/) [46]. Then, the interacting residues were visualized by the SPICE server (https://spice.cs.ucr.edu) [47].

#### Immune Simulation

The immune profile elicited by the designed MEV was simulated using the C-ImmSim web server (https://kraken.iac.rm.cnr.it/C-IMMSIM/). C-ImmSim employs a combination of position-specific scoring matrices (PSSM) and machine learning algorithms to simulate immune responses [48]. The simulation parameters were defined with a stimulation volume of 10 and 168 stimulation steps (56 days). The default simulation volume (10) and host immunological parameters were used. Three antigen administrations were simulated at 14-day intervals. B-cell populations were evaluated across functional states, including activated, antigen-internalizing, MHC class II–presenting, duplicating (proliferating), anergic, and resting cells. Cytokine responses (*e.g.*, IFN-γ, IL-2, IL-4, IL-6, IL-10, IL-12, TNF-α, and TGF-β) and antibody levels were recorded throughout the simulation to assess the humoral and cellular immune response following each antigen exposure.

## Results

### Pan/core analysis of proteomes

**Fig. 2:**
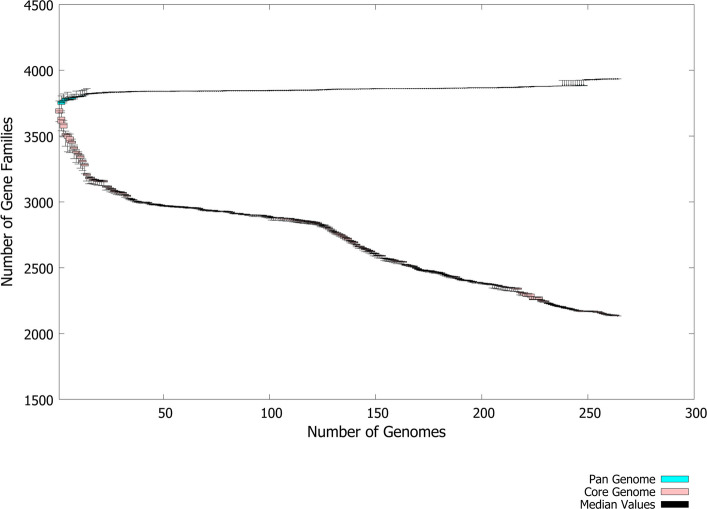
The core-pan plot of 256 *M. tuberculosis* strains generated using BPGA software. The identity > 0.8 was considered. Core proteins account for 49% of pan-proteins. The numbers of core, pan, and accessory genes were 2134, 4354, and 1720, respectively

The numbers of core, pan, and accessory genes were 2134, 4354, and 1720, respectively. Core proteins were selected to investigate common immunogenic targets against *M. tuberculosis* strains. A core-pan plot is shown in Fig. 2. The core proteins account for 49% of the pan-proteome of *M. tuberculosis* strains. The B-parameter was 0.0173889, which presents the almost closed pan-genome of *M. tuberculosis*.

### Identification of immunogenic proteins

#### Screening the surface-exposed and high-prevalent proteins

Considering the 2134 core proteins of 256 *M. tuberculosis* and the 3901 CDSs of *M. tuberculosis* strain H112, a total of 80 surface-exposed proteins consisting of 67 extracellular proteins and 13 cell wall-associated proteins were identified. Out of 80 surface-exposed proteins, 69 had a prevalence > 90% among 256 *M. tuberculosis* strains.

Sixty-five proteins had ≤ 1 transmembrane helix, while four proteins were located inside the membrane and were excluded. On the next step, 48 antigenic proteins with a score > 0.5 were identified. Among antigenic proteins, seven allergens were investigated and excluded from the study. Then, 10 proteins with sequence similarity to the human proteome were excluded, and 31 surface-exposed, antigenic and non-allergen proteins with no similarity to the human proteome remained. See Supplementary data 1 and Table 1.

**Table 1:** Thirty-one proteins were retrieved through screening different characteristics including subcellular localization, transmembrane helices, antigenicity, allergenicity, similarity to human proteome, linear B-cell epitope ratios, and MHC II binding site ratios

No	Protein Name	Accession Number	Subcellular localization (Psortb)	TMH^*^	Antigenicity (cut-off = 0.5)	Allergenicity (cut-off = 0.5)	Human Blast	Antigenicity (cut-off = 0.5)	Linear B-cell epitopes ratio	MHC II binding sites ratio
1	C40 family peptidase	WP_003901769.1	Extracellular	Outside	0.5015	Non-Allergen (−0.46)	-	0.5015	0.1	0.146
2	Low molecular weight antigen MTB12	WP_003412264.1	Extracellular	Outside	0.5110	Non-Allergen (0.25)	-	0.511	0.061	0.109
3	Peptidoglycan hydrolase RipC	WP_003411373.1	Extracellular	Outside	0.5221	Non-Allergen (0.38)	-	0.5221	0.147	0.118
4	Adhesin Apa	WP_003409337.1	Extracellular	Outside	0.5264	Non-Allergen (0.31)	-	0.5264	0.095	0.071
5	DUF1906 domain-containing protein	WP_003412957.1	Extracellular	Outside	0.5407	Non-Allergen (0.36)	-	0.5407	0.07	0.094
6	Hypothetical protein	WP_003898724.1	Extracellular	Outside	0.5418	Non-Allergen (0.38)	-	0.5418	0	0.057
7	Class A beta-lactamase BlaC	WP_003410677.1	Extracellular	Outside	0.5478	Non Allergen (−0.48)	-	0.5478	0.112	0.102
8	ABC transporter substrate-binding protein	WP_003901692.1	Cell wall	Outside	0.5538	Non-Allergen (0.22)	-	0.5538	0.019	0.104
9	Ag85B	WP_003409456.1	Extracellular	Outside	0.5842	Non-Allergen (0.28)	-	0.5842	0.05	0.159
10	DNA polymerase III subunit delta	WP_003903885.1	Cell wall	Outside	0.5867	Non-Allergen (0.03)	-	0.5867	0	0.192
11	Heparin-binding hemagglutinin HbhA	WP_003402339.1	Cell wall	Outside	0.5986	Non-Allergen (−0.36)	-	0.5986	0.109	0.057
12	Immunoprotective protein Mpt64	WP_003409954.1	Extracellular	Outside	0.6005	Non Allergen (−0.33)	-	0.6005	0	0.111
13	RipB	WP_003407525.1	Extracellular	Outside	0.6120	Non-Allergen (−0.35)	-	0.612	0.185	0.09
14	Type VII secretion system ESX-5 target PE18	WP_003408976.1	Extracellular	Outside	0.6240	Non-Allergen (−0.21)	-	0.624	0	0.31
15	Stress-responsive chaperone Acr2	WP_003900838.1	Cell wall	Outside	0.6248	Non-Allergen (−0.35)	-	0.6248	0.104	0.111
16	Phosphoglycerate mutase PE_PGRS11	WP_034161830.1	Extracellular	outside	0.6446	Non-Allergen (0.38)	-	0.6446	0.078	0.164
17	RipA	WP_003407523.1	Extracellular	Outside	0.6576	Non-Allergen (0.11)	-	0.6576	0.233	0.104
18	Hypothetical protein	WP_009937839.1	Extracellular	Outside	0.7749	Non-Allergen (0.14)	-	0.7749	0	0.27
19	Alpha-crystallin HspX	WP_003410189.1	Cell wall	Outside	0.8053	Non Allergen (−0.43)	-	0.8053	0.042	0.159
20	Resuscitation-promoting factor protein RpfE	WP_224994151.1	Extracellular	Outside	0.8055	Non-Allergen (0.38)	-	0.8055	0.065	0.037
21	PPE family protein	WP_031730943.1	Extracellular	outside	0.8490	Non-Allergen (−0.19)	-	0.849	0.654	0.079
22	PPE family protein	WP_003904827.1	Extracellular	Outside	0.8764	Non-Allergen (−0.18)	-	0.8764	0.54	0.14
23	DUF3060 domain-containing protein	WP_003901898.1	Extracellular	Outside	0.9613	Non-Allergen (0.41)	-	0.9613	0.195	0.045
24	Hypothetical protein	WP_003901458.1	Extracellular	Outside	0.9696	Non-Allergen (0.18)	-	0.9696	0.36	0.09
25	PE family protein	WP_003412039.1	Extracellular	Outside	1.0461	Non-Allergen (−0.3)	-	1.0461	0.36	0.17
26	PE family protein	WP_031651465.1	Extracellular	Outside	1.7386	Non-Allergen (−0.13)	-	1.7386	0.282	0.098
27	Hypothetical protein	WP_015631092.1	Extracellular	Outside	1.8485	Non-Allergen (0.3)	-	1.8485	0.219	0.035
28	PE family protein	WP_003910446.1	Extracellular	Outside	1.9053	Non-Allergen (−0.15)	-	1.9053	0.353	0.083
29	Hypothetical protein	WP_003401880.1	Extracellular	Outside	2.1849	Non-Allergen (0.37)	-	2.1849	0.375	0.02
30	Hypothetical protein	WP_003411064.1	Extracellular	Outside	2.3300	Non-Allergen (0.32)	-	2.33	0.372	0.051
31	PE family protein	WP_031651290.1	Extracellular	Outside	2.5362	Non-Allergen (0.35)	-	2.5362	0.472	0.06

TMH: Transmembrane helices

#### Selection of immunogenic proteins based on linear B-cell epitopes and MHC II binding sites

**Fig. 3:**
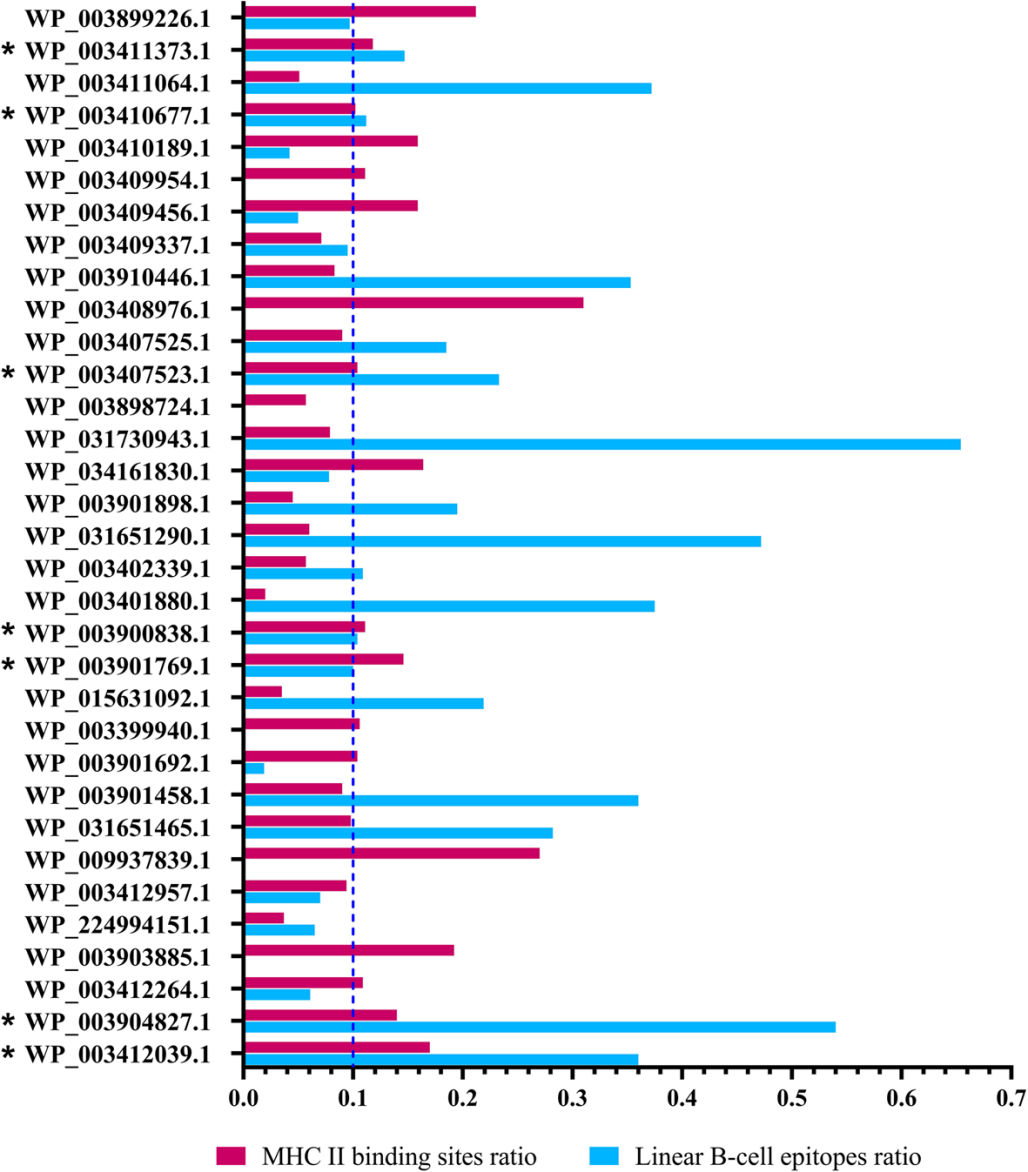
Selection of immunogenic proteins using linear B-cell epitope ratio and MHC II binding sites ratio. Seven proteins with ratios > 0.1 for both epitope types were selected as shortlisted proteins. The blue dotted line shows the cut-off

To select the promising immunogen proteins, the B-cell epitopes ratio and MHC II binding sites ratio of proteins were calculated. The B-cell epitopes ratio varied between 0 and 0.661, whereas the MHC II ratio ranged from 0.02 to 0.31. Finally, seven proteins with ratios > 0.1 for both B-cell epitopes and MHC II binding sites, were selected (Fig. 3).

#### Detection of conformational B-cell epitopes

**Fig. 4:**
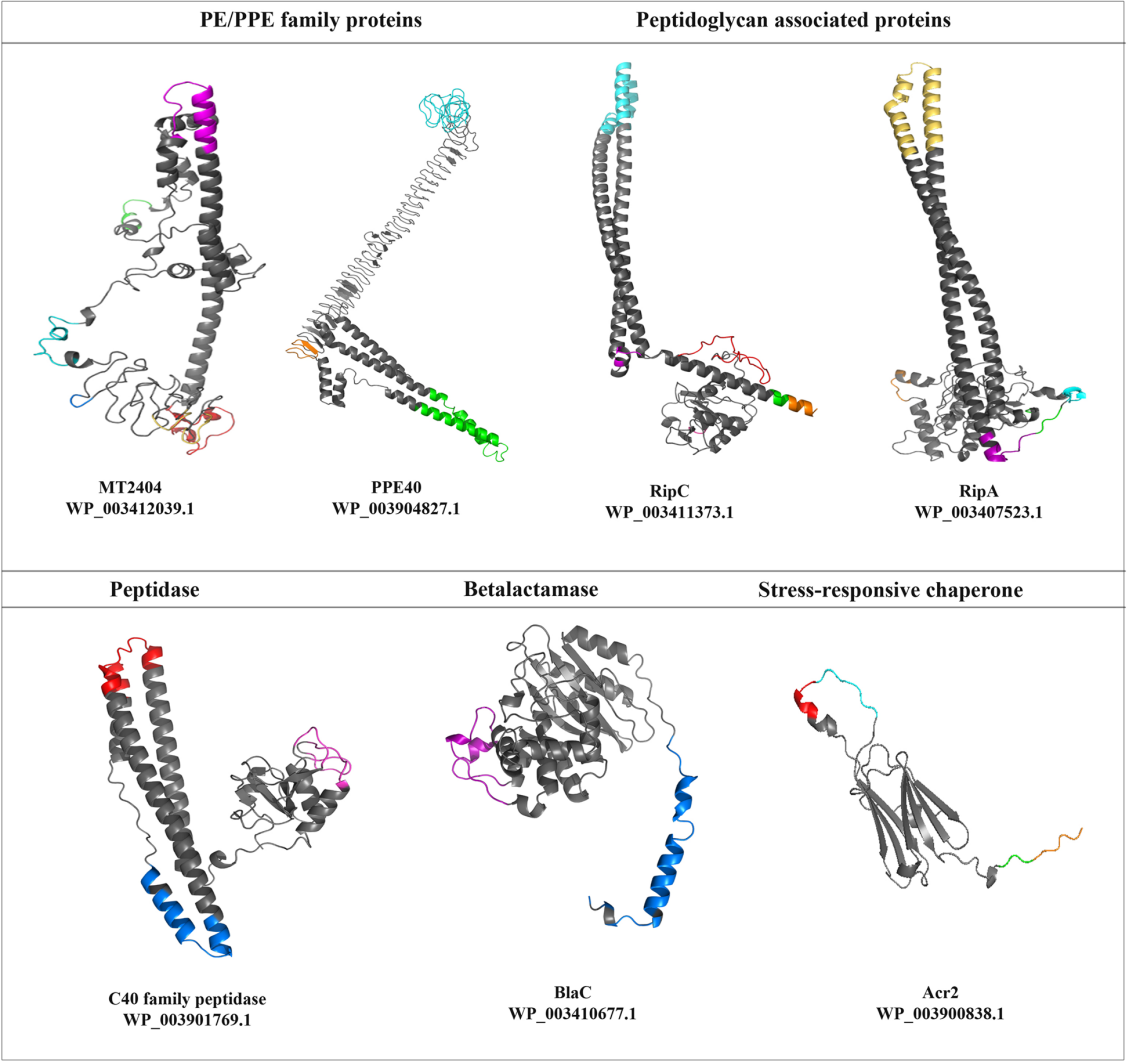
Conformational B-cell epitopes of seven immunogenic proteins against *M. tuberculosis.* The epitopes were identified using ElliPro with a cut-off > 0.8. Gray color shows the backbone of proteins and other colors each are used to show a distinct conformational B-cell epitope. RipC (WP_003411373.1) and MT2404 (WP_003412039.1) had the highest number of conformation B-cell epitopes

**Fig. 5:**
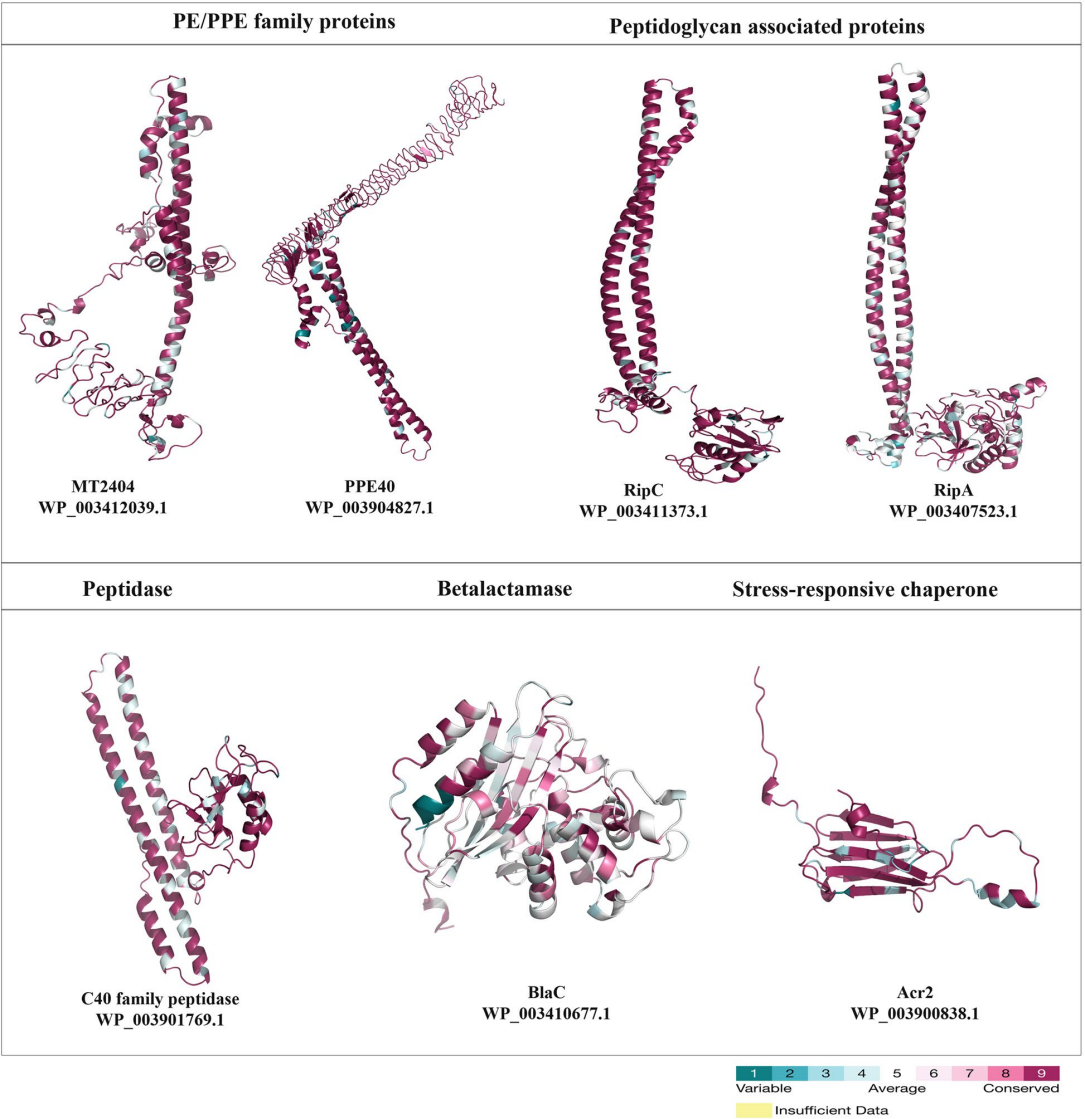
Sequence conservation of seven immunogenic targets against *M. tuberculosis* using the ConSurf database. Deep magenta and dark turquoise colors show the most conserved and variable regions, respectively. All proteins showed high sequence conservancy among *M. tuberculosis* strains

The conformational B-cell epitopes of the selected proteins were determined and are shown in the 3D structure of each protein in Fig. 4. RipC (WP_003411373.1) and MT2404 (WP_003412039.1) had the highest number of conformation B-cell epitopes. The detailed information of sequence and color of conformational epitopes are demonstrated in Supplementary Data 2. On the next step, the conservancy of proteins were assessed. Fortunately, the shortlisted proteins showed high sequence conservation among *M. tuberculosis* strains, which is an essential feature to select a protective antigen for vaccine development (Fig. 5).

### Shortlisted proteins

A total of seven proteins is introduced as promising immunogenic targets against *M. tuberculosis*. The proteins are as follows: two PE/PPE family proteins (MT2404: WP_003412039.1; and PPE40: WP_003904827.1), one stress-responsive chaperone (Acr2: WP_003900838.1), one beta-lactamase (BlaC: WP_003410677.1), two peptidoglycan-associated proteins (RipC: WP_003411373.1, and RipA: WP_003407523.1), and one peptidase (C40 family peptidase: WP_003901769.1). The functional properties of shortlisted proteins were investigated through the EggNOG and CDD databases. Three proteins, RipC, RipA, and C40 family peptidase, were associated with peptidoglycan and cell wall biogenesis. MT2404 and PPE40 belong to the PE/PPE protein family, although their precise functions remain unknown. Acr2 belongs to the small heat shock protein (HSP20) family and is involved in post-translational modifications and protein turnover. BlaC is a beta-lactamase class A from the PenP superfamily (Table 2).

**Table 2:** Functional properties of seven shortlisted immunogenic targets against *M. tuberculosis* based on the conserved domains and EggNOG classification

Protein (accession number)	Conserved domains	EggNOG
MT2404 (WP_003412039.1)	PE superfamily (high glycine content)	PE family protein
PPE40 (WP_003904827.1)	COG5651 super family Proline–Proline–Glutamate (PPE) Family Unknown function	Cell motility PPE family protein
Acr2 (WP_003900838.1)	IbpA superfamily HSP20 family Post-translational modification	Post-translational modification, protein turnover, and chaperones Belongs to the small heat shock protein (HSP20) family
BlaC (WP_003410677.1)	PenP super family Beta-lactamase class A	Defense mechanisms Beta-lactamase enzyme family
RipC (WP_003411373.1)	wall_hydro_RipC superfamily a peptidoglycan hydrolase	Function unknown cell wall organization
RipA (WP_003407523.1)	NlpC_p60_RipA peptidoglycan endopeptidase	Function unknown cell wall organization
C40 family peptidase (WP_003901769.1)	NLPC_P60 super family Unknown function, found in several lipoproteins	Cell wall/membrane/envelope biogenesis cysteine-type peptidase activity

### Identification of immunogenic peptides

#### Identification of immunoreactive epitopes

A total of 4718 immunoreactive linear B-cell epitopes of *M. tuberculosis* were retrieved from the IEDB database. Comparison of the 80 surface-exposed core proteins with the epitopes resulted in the identification of 2284 immunoreactive epitopes that 719 non-redundant epitopes with 100% identity were determined. Only one epitope resembled the human proteome, and 718 non-homologous epitopes remained. See Supplementary data 3.

#### Identification of epitopes confirmed in experimental assays

All selected epitopes were submitted to the IEDB database to find epitopes that are involved in experimental immunological assays. Our results represented that out of 718 epitopes, 460 were IFN-γ releasing, 229 were involved in proliferation, 176 were involved in qualitative binding, 50 had a role in cytotoxicity, 28 were IL-4 releasing, 18 were TNF-α releasing, 6 had a role in pathogen burden after challenge, 6 were perforin releasing, and 5 epitopes release granzyme A. See Supplementary data 3.

#### Selecting the highly conserved, antigenic, and non-allergen epitopes

To identify the most conserved epitopes among *M. tuberculosis* strains, the IEDB Conservancy database was used. The conservancy of IFN-γ releasing epitopes ranged from 34 to 100%. Out of 460 epitopes, only 152 epitopes with a conservancy score > 95% were selected. Out of 152 conserved epitopes, 29 antigenic epitopes were identified with an antigenicity score > 1. Consequently, 14 non-allergens were retrieved. See Supplementary Data 4. Among 14 epitopes, seven epitopes showed significant homology to TCR binding epitopes and are introduced as novel immunogenic epitopes for immunization against *M. tuberculosis* (Table 3).

**Table 3:** Analyses of immunogenic epitopes for development of a MEV (multi epitope vaccine) against *M. tuberculosis*. Fourteen conserved, IFN-γ releasing, antigenic, and non-allergen epitopes were identified

Peptide^*^	Relevant protein	Conservancy	Ag score	Ag/non-Ag		Allergenicity	TCR epitopes resemblance	Cov ^✝^	Iden ^•^
**DIKVQFQSGGANSPALYLLD**	Ag85a	96.23%	1.3288	Ag		Non-Allergen	DIKVQFQSGGAN	60	100
							DIKVQFQSGG	50	100
**GNGKPSDLGGNNLPAKFLEG**	Ag85a	96.23%	1.2838	Ag		Non-Allergen	SDLATNNL	40	75
							GDGKMKDL	40	62
**FYSDWYQPACGKAGC**	Ag85a	98.11%	1.1331	Ag		Non-Allergen	YMAWYQQTPGKA	80	58.33
							FTSDYYQ	46	71.43
**AYNAGGGHNGVFDFP**	Ag85a	96.23%	1.3298	Ag		Non-Allergen	AYNAAGGHNAVF	80	83.33
**KVQFQSGGANSPALY**	Ag85a	96.23%	1.1385	Ag		Non-Allergen	KVQFQSGGAN	66	100
							KVQFQSGG	53	100
							QSTPARSPA	60	66.67
**SRADEEQQQALSSQMGF**	Esxb	95.12%	1.0284	Ag		Non-Allergen	DEQRAAAL	47	50
**RADEEQQQALSSQMGF**	Esxb	95.12%	1.032	Ag		Non-Allergen	DEQRAAAL	50	50
**QYSRADEEQQ**	Esxb	97.56%	1.1832	Ag		Non-Allergen	-	-	-
**EMKTDAATL**	Esxb	95.12%	1.282	Ag		Non-Allergen	TDPVTL	66	66.67
							TDLAT	55	80
**QVPSASMGRDIKVQF**	Ag85c	96.23%	1.4152	Ag		Non-Allergen	QVPSASMGRDIKVQF	100	100
							VPSPSMGRDIKVQF	93	82.86
							PSPSMGRDIKVQF	86	92.31
							SMGRDIKVQF	66	100
							MGRDIKVQF	60	100
**GQSVTGYNNSVSVTS**	Ppe12	96.27%	1.2471	Ag		Non-Allergen	YN–-SVTS	60	66.67
							YN–-SVTS	60	66.67
**APKTYCEELKGTDTGQACQI**	Mpt64	90.20%	1.3385	Ag		Non-Allergen	-	-	-
**TIKAERTEQKDFDGR**	Hspx	95.65%	1.7884		Ag	Non-Allergen	-	-	-
**VGADEDDIKATYDKG**	Hspx	95.65%	1.1029		Ag	Non-Allergen	-	-	-

^*^The underlined epitopes were selected to design the MEV

✝ Coverage

• Identity

#### Structural evaluation of the MEV

**Fig. 6:**
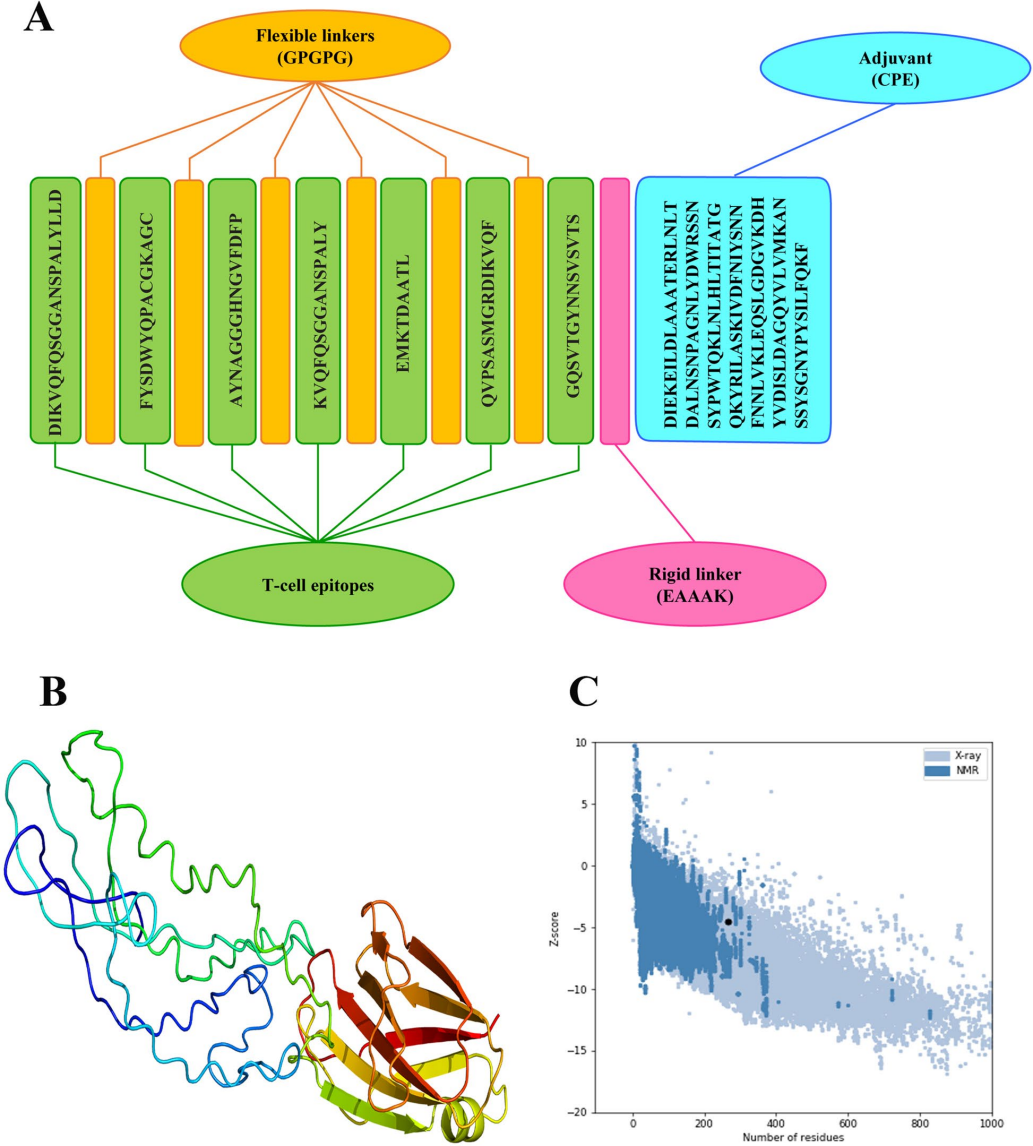
**A** Schematic representation of the MEV construct. Immunodominant epitopes were joined together by GPGPG flexible linkers, and the construct was fused to the C-terminal of CPE using a rigid EAAAK linker. **B** The tertiary structure of MEV was predicted by homology modeling using the I_TASSER web server. The 3D structure was visualized using PyMOL software. **C** The 3D structure of MEV was validated using the ProSA-web server. The Z-score of the structure was −4.53

Seven immunogenic epitopes with high similarity to TCR epitopes were joined together using GPGPG flexible linkers. Then the 194–319 aa C-terminal fragment of CPE was attached to the C-terminal of the construct using an EAAAK rigid linker (Fig. 6). The corresponding PDB file is available as Supplementary data 5. The construct was antigenic (Score = 0.96) and non-allergen, with a molecular weight of 27.85 kDa. The protein was soluble (Score = 0.52) with no similarity to the human proteome and no toxicity. The theoretical pI was 5.57, and the protein was hydrophilic (GRAVY score = −0.37). Secondary structure analysis revealed that 13%, 8% and 32% of the amino acids form disordered regions, alpha helices and Beta sheets, respectively (Table 4).

**Table 4:** Investigating the first and second structure properties of the designed MEV against *M. tuberculosis*

Features	Output
Molecular weight (kDa)	27.85
Theoretical pI	5.58
Total number of negatively charged residues (Asp + Glu)	21
Total number of positively charged residues (Arg + Lys)	18
Instability index	Stable (33.78)
Aliphatic index	69.62
Grand average of hydropathicity (GRAVY)	−0.37
Antigenicity	Antigen (0.96)
Allergenicity	Non-allergen
Homology to human proteome	No similarity
Disordered	13%
Alpha helix	8%
Beta strand	32%
Solubility	Soluble (0.52)
Toxicity	Non-toxin

**Fig. 7:**
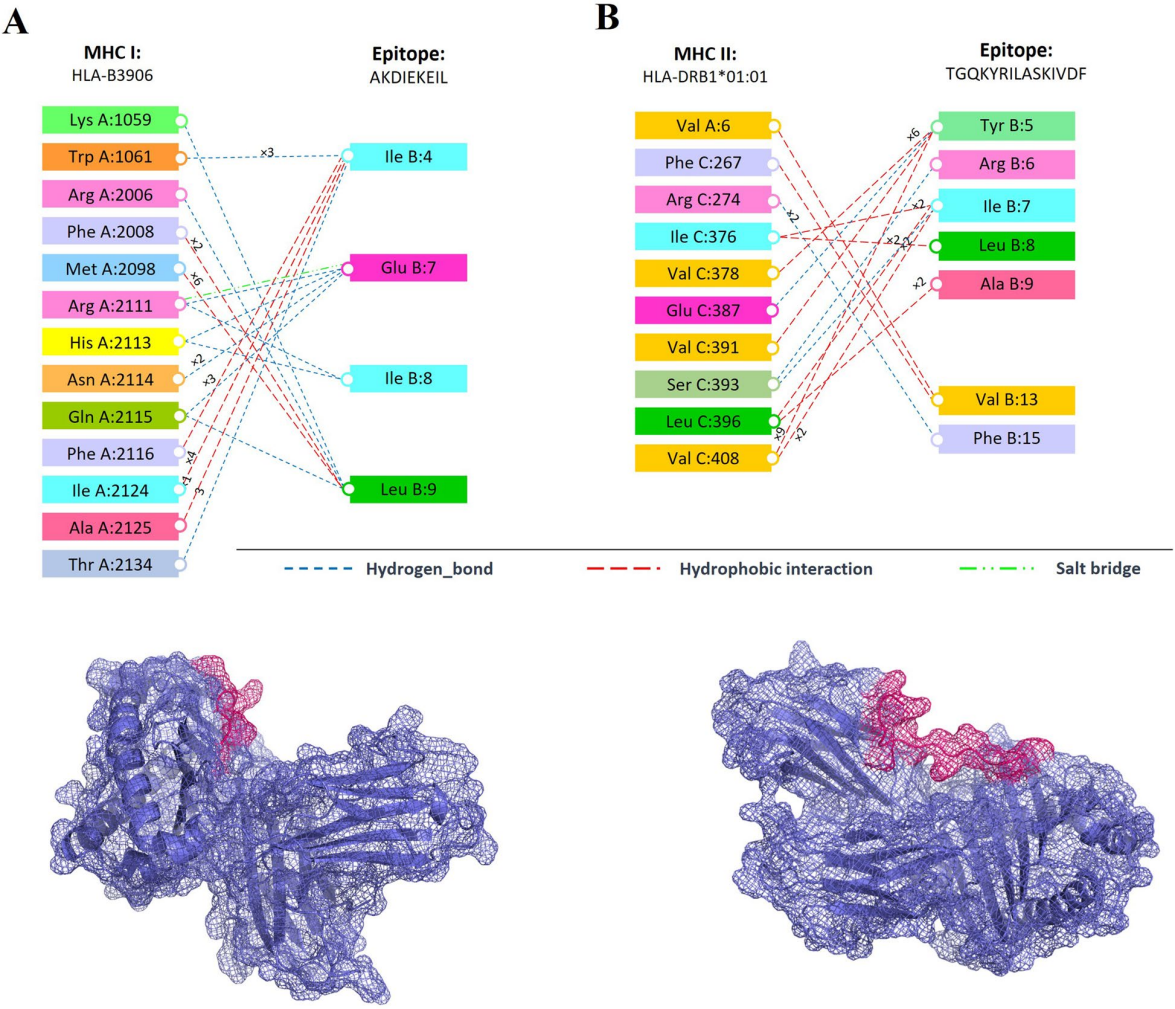
**A** Molecular docking of MHC I (HLA-B3906) and MHC I epitope (AKDIEKEIL). The interacting residues were visualized using the SPICE. A total of 29 hydrophobic interactions, 13 hydrogen bonds and one salt bridge were formed between the two components. **B** Molecular docking of MHC II (HLA-DRB1*01:01) and MHC II epitope (TGQKYRILASKIVDF). Thirty hydrophobic interactions, and five hydrogen bonds were observed between MHC II allele and its epitope. Interpretation of interactions: Blue dotted lines: hydrogen bonds, and red dotted lines: hydrophobic interactions, green dotted lines: salt bridges

A total of 10 MHC I and 30 MHC II epitopes were detected in the designed MEV (Supplementary data 6). Among the MHC class I epitopes, AKDIEKEIL exhibited the highest score and was selected for molecular docking. Similarly, among MHC II binding epitopes, TGQKYRILASKIVDF demonstrated the highest score. The molecular docking of the epitope AKDIEKEIL and HLA-B39 showed a total of 29 hydrophobic interactions, 13 hydrogen bonds and one salt bridge between the two components, with a docking score of −106.37. While the molecular docking of the epitope TGQKYRILASKIVDF and HLA-DRB1*01:01 showed thirty hydrophobic interactions, and five hydrogen bonds between two components, with a docking score of −172.29. The docking information of MEV and selected MHC Class I and MHC Class II molecules is presented in Table 5 and Fig. 7.

**Table 5:** The docking information of MEV and MHC Class I and MHC Class II molecules

MHC allele	Epitope	Docking Score	Confidence Score	Ligand rmsd (Å)
HLA-B39	AKDIEKEIL	−106.37	0.2947	46.87
HLA-DRB1*01:01	TGQKYRILASKIVDF	−172.29	0.6096	181.36

#### Immune simulation

**Fig. 8:**
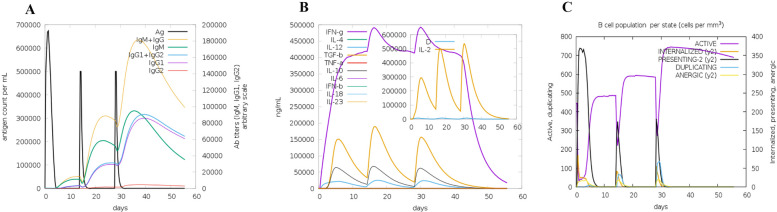
C-ImmSim immune simulation of the multi-epitope vaccine administered on days 0, 14, and 28. **A** Humoral immune responses showing rapid antigen clearance, primary IgM production, and enhanced IgG/IgG1 responses following booster doses. **B** Cytokine responses characterized by increased IL-2, IFN-γ, and TNF-α levels, indicating strong T-cell activation and a Th1-oriented immune response. **C** B-cell dynamics demonstrating expansion of activated, proliferating, antigen-processing, and MHC-II-presenting B cells, followed by enrichment of memory-like B cells, suggesting the development of long-term immune memory

The immune response elicited by the three-dose vaccination strategy was simulated using the C-ImmSim platform. As shown in Fig. 8A, each immunization step (days 0, 14, 28) resulted in rapid antigen clearance accompanied by a pronounced humoral immune response. Initial immunization induced a primary IgM response, whereas subsequent booster doses led to a substantial increase in IgG antibodies, particularly IgG1 subclass. The progressive elevation of IgG titers after the second and third injections shows effective class switching from IgM to IgG. The cytokine profile (Fig. 8B) demonstrated marked increases in key immune mediators following each antigen administration. Notably, elevated levels of IL-2, IFN-γ, and TNF-α were observed, suggesting strong T-cell activation and a Th1-skewed immune response. The repeated cytokine peaks after booster doses indicate sustained immune stimulation.

Additionally, B-cell population changes also supported this pattern. After each antigen dose, the number of active B cells increased quickly, and the duplicating B-cell population also rose, showing that these cells were expanding and multiplying in response to the vaccine. At the same time, more B cells became antigen-internalizing and MHC class II–presenting cells, indicating that antigen uptake and presentation to helper T cells were actively occurring. After the final immunization, the levels of activated and duplicating B cells declined, while resting and memory-like cells became more prominent, suggesting that the early response was contracting and immune memory was being established (Fig. 8C).

## Discussion

Following the ineffectiveness of BCG in adults owing to pre-sensitization with environmental Mycobacteria, the need for the development of an efficacious vaccine that is protective in both infants and adults, and able to overcome the shortcomings of BCG is inevitable [49]. Recently, the next generation of vaccines are being studied for immunization against *M. tuberculosis*. Several studies have taken place on the development of recombinant BCG vaccines. VPM1002, a recombinant BCG, with the addition of listeriolysin and deletion of urease, has the capability to enter the host cell cytoplasm, and escape the macrophage lysosome. Although VPM1002 was less reactogenic in comparison to BCG, its immunogenicity was not inferior [13]. Given the limitations of conventional and recombinant BCG-based vaccines, increasing attention has been directed toward reverse vaccinology and immunoinformatics approaches for the development of novel multi-epitope vaccines against *M. tuberculosis*. For example, Moodley et al*.* employed reverse vaccinology and immunoinformatics approaches to design a multi-epitope vaccine against *M. tuberculosis* based on the PE_PGRS17 biomarker. In their study, crucial immunogenic properties, including antigenicity, allergenicity, and toxicity, were evaluated for the target protein, selected B-cell and T-cell epitopes, and the final multi-epitope vaccine construct [50]. Moreover, Hasan et al*.* utilized the Vaxign2 pipeline to identify several promising antigenic proteins, including PPE35, Mpt83, MrsA, and RplK. After screening CTL, HTL, and B-cell epitopes based on their antigenicity, non-allergenicity, and non-toxicity, they constructed a multi-epitope vaccine incorporating the PADRE adjuvant and appropriate linkers to enhance immune responses [51]. Similarly, Bibi et al*.* designed a novel multi-epitope subunit vaccine using highly conserved and experimentally validated antigens, including Rv2608, Rv2684, Rv3804c (Ag85A), and Rv0125 (Mtb32A). B-cell and T-cell epitopes were predicted using the IEDB platform, and Griselimycin was included as an adjuvant to improve the immunogenicity of the final construct [52]. Despite recent advances in immunoinformatics and reverse vaccinology approaches for *M. tuberculosis* vaccine design, the identification of stable, highly immunogenic, and safe multi-epitope vaccine candidates remains a major challenge, which justifies further investigation in this field.

As already mentioned, considering the clinical significance of TB, low efficiency of BCG vaccination in adolescents and adults, and the high mortality rates in recent years [2], efforts to develop a protective vaccine against this pathogen are still ongoing. Taking this into account, the present study deals with immunization against *M. tuberculosis* on two levels. First, seven promising immunogenic targets were introduced for further evaluation. The appropriate features of the vaccine, including exposure on the cell surface, antigenicity, prevalence and conservation among circulating *M. tuberculosis* strains, and predicting immunogenic epitopes were considered to select these immunogenic targets.

In the next step, we aimed to identify the immunogenic peptides and presented them in the form of a MEV as a proposed novel array for protecting against *M. tuberculosis*. Previous clinical trials on the efficiency of recombinant protein vaccines against *M. tuberculosis* showed the superior potential of MEVs to integrate the immunogenic epitopes of different vaccine candidates [53]. This study is particularly noteworthy as it provides a large-scale genomic analysis of 256 complete *M. tuberculosis* strains, enhancing the reliability of its findings.

Granuloma formation and cell-mediated immunity are the major mechanisms of the immune response against *M. tuberculosis* [54]. Th1 and Th17 cells are involved in protection against *M. tuberculosis*. T CD4 + cells produce IFN-γ and TNF-α, which promote the killing of *M. tuberculosis* through phagosome maturation in macrophages [55, 56]. The identification of potential candidates that elicit strong IFN-γ is the major approach for the development of subunit vaccines against *M. tuberculosis* because IFN-γ is a key cytokine involved in the innate immune response and is mainly produced by immune cells activated through HTL stimulation [51, 57]. Thus, in this study, IFN-γ releasing epitopes were taken into consideration to develop a protective vaccine.

On the other hand, to induce a protective immune response against the majority of circulating *M. tuberculosis* strains, only epitopes with a conservancy > 95% were selected. Apart from considering the immunogenic properties of the epitopes, to minimize cross-reactivity with the host, peptides that have no similarity to the *Homo sapiens* proteome were prioritized.

Different TCRs recognize specific epitopes that are presented by MHC class I or II on the cell surface [58]. Therefore, to trigger cellular immunity for protection against *M. tuberculosis*, we selected seven epitopes that are identical to TCR binding epitopes, to design the MEV. These epitopes were derived from Ag85A, Ag85C, EsxB, and PPE family protein.

The Ag85 complex is composed of Ag85A, Ag85B, and Ag85C. This complex plays a significant role in host immune evasion, by binding to mycolyl-transferase and fibronectin, and subsequently prevents the formation of phagolysosomes. This complex has been widely used in the development of diagnostic approaches, and protective vaccines [59]. Ag85A has been evaluated as a vaccine candidate against *M. tuberculosis* several times. GamTBvac was formulated by fusing Ag85A and ESAT6-CFP10, and revealed desirable safety and immunogenicity in phase I and II clinical trials [60, 61]. This protein has also been employed in some viral-vectored vaccines against *M. tuberculosis* [62, 63].

The Esx family comprises 23 proteins that are associated with host‒pathogen interactions in *M. tuberculosis*. These immunodominant proteins have been extensively used to generate diagnostic methods and vaccines for tuberculosis [64]. Five IFN-γ producing Esx proteins (EsxB, EsxD, EsxG, EsxU, and EsxM), were utilized to design a fusion protein against *M. tuberculosis*. Hopefully, administration of the fusion protein showed desirable protection against *M. bovis* challenge [57].

Two proteins of the shortlisted vaccine candidates (MT2404 and PPE40) and one of the selected epitopes (PPE12) were from PE (proline-glutamic acid)/PPE (proline-proline-glutamic acid) family proteins. Since PE/PPE families are more abundant in pathogenic genera than in nonpathogenic Mycobacteria, they seem to be associated with the pathogenicity of Mycobacteria [65]. These genes account for almost 10% of the protein-coding genes of *M. tuberculosis*. PE/PPE family members modulate the host immune response through interaction with human TLRs [66]. These families are understudied due to weird characteristics such as high G + C% that interfere with cloning, sequencing, and alignment. However, these proteins are regarded as highly immunogenic owing to the induction of IFN-γ. Although scientists believe that some PE/PPE proteins might hamper the inflammatory responses in the host and evade immune surveillance, using particular immunodominant epitopes of these proteins might assist with the development of effective vaccines against *M. tuberculosis* [65]. Several antigens belonging to this family have been targeted for immunization against TB. For instance, a chimpanzee adenovirus expressing the PPE15 antigen of *M. tuberculosis* was administered intranasally and improved protection of BCG vaccine [67]. To enhance the vaccine’s efficacy, Stylianou E et al*.* developed a five-antigen fusion by combining PPE15 with three members of the Esx-5a secretion system and Ag85A into a multi-antigen construct. The fusion antigen vaccine was administered through mucosal route. ChAdOx1.5Ag enhanced the immunity of BCG in immunized mice [68].

Moreover, considering the evidence on antigenicity and immunogenicity of PE/PEE families [69, 70], a recent computational study has targeted these proteins to design a MEV against *M. tuberculosis* [71]. In addition, M72/AS01E which targets PPE18 could stop the progression of latent TB to active infection in 50% of the participants in phase II clinical trial [72]. Thus, the phase III clinical trial on M72/AS01E has been launched in March 2024 [73].

Acr2 was another shortlisted protein, which belongs to small heat shock protein (HSP20) family. Previously, Acr2-specific IgG and IgA antibody titers were found to be elevated in individuals with latent TB infection compared to uninfected controls, suggesting that this antigen is immunogenic and these findings imply that Acr2 possesses the ability to elicit antibody responses and could be a potential immunogenic component in TB vaccination [74].

RipA and RipC are involved in cell wall organization. RipA modulates host immune responses and promots intracellular survival. It activates the TLR4–NFκB signaling pathway, leading to the secretion of pro-inflammatory cytokines and M1 macrophage polarization. Additionally, it inhibits autophagy by activating the PI3K-AKT-mTORC1 pathway and suppressing ULK1. It further prevents apoptosis in infected macrophages. Together, these mechanisms enable RipA to create a permissive intracellular niche that facilitates *M. tuberculosis* survival and replication [75]. Although RipC is not required for survival under normal conditions, it plays a crucial role in cell elongation and division, making it essential for bacterial survival under stress [76]. BlaC, another potential vaccine candidate, is a class A extended-spectrum β-lactamase that hydrolyses β-lactam antibiotics [77].

Subunit vaccines are presented whether on viral vectors, in combination with adjuvants or in lipid nanoparticle-mRNA platform. The viral-vectored vaccines have the capacity to induce high levels of humoral, cellular and long-term memory immunity. Despite having notable advantages, including safety, no need for adjuvants, easy manipulation and robust memory immunity after a single administration of the vaccine, this approach comes with some drawbacks. Pre-existing anti-vector antibodies might impede the efficacy of viral vectored vaccines. Additionally, anti-vector responses mounted by the host's immune system have the potential to hinder the effects of booster doses, and most importantly, viral vectors are not suitable to immunize immunocompromised people [78]. Following the successful development of LNP-mRNA vaccines against SARS-CoV-2, using this approach for prevention of TB is gaining more attention. Nevertheless, its effectiveness in preventing persistent bacterial infections like TB is still ambiguous [79]. BNT164a1 and BNT164b1 are two multi-antigenic mRNA-based vaccines against TB that are being evaluated in phase I clinical trial [80].

However, subunit vaccines can be administered in conjugation with an adjuvant [49]. Thus, we decided to use a targeted adjuvant to induce strong mucosal immune responses against *M. tuberculosis*. BCG vaccine is generally administered via intradermal injection, which induces strong systemic immune responses but not mucosal immunity. Scientists hypothesize that the development of a novel vaccine that mimics the route of entry of *M. tuberculosis* is more successful in inducing robust immune responses at the site of entry [81]. Therefore, efforts are focused on the development of a mucosal vaccine against *M. tuberculosis*. Currently, there are no approved adjuvants for intranasal delivery in humans. However, certain compounds (*e.g., E. coli* LT, type 1 IFN, poly-i CLC, oleic acid, cholera toxin B subunit, etc.) have undergone clinical studies to explore their potential as mucosal adjuvants [82–85].

In this study, a MEV against *M. tuberculosis* was designed using computational immunoinformatics approaches, which closely mirrors the methodology employed by Yun et al*.* [86]. However, a notable difference is our inclusion of the C-terminal fragment of *C. perfringens* enterotoxin (CPE) as an adjuvant for mucosal immunity against *M. tuberculosis* for the first time. The C-terminal fragment of CPE has been found to lack cytotoxic effects, but it exhibits significant efficacy in mediating receptor binding. Claudins are the cellular receptors of CPE [87]. These specific proteins are tight junction membrane proteins that are situated at the apical contact region between epithelial and endothelial cells. They play a pivotal role in the modulation of paracellular permeability of epithelia [88]. The safety and efficacy of CPE in inducing nasal immune responses has been stated in several previous studies [89–91].

To generate the MEV, the C-terminal fragment (194–319 aa) of CPE was fused to the immunogenic epitopes of *M. tuberculosis*. The aforesaid fragment harbors high solubility and affinity toward claudins. Moreover, it increases the absorption of drugs in mucosal tissue membranes [41]. Surprisingly, the MHC I and MHC II epitopes of the MEV showed desirable interactions with human HLA-B39 and HLA-DRB1*01:01 in molecular docking. Moreover, this MEV may have the potential to be used as a nasal vaccine against *M. tuberculosis* and to induce mucosal immune responses against this pathogen. Taken together, this study introduces a novel multi-epitope vaccine against *M. tuberculosis* using immunoinformatics and protein engineering. In in silico vaccine design studies, variations in computational tools, algorithms, and selection thresholds often lead to discrepancies in the final list of predicted vaccine candidates across different studies. Therefore, despite the value of these predictive approaches in narrowing down potential targets, the immunogenicity and protective efficacy of the proposed candidates must be rigorously validated through comprehensive experimental evaluations, including both in vitro and in vivo studies. Therefore, one of the limitations of the present study is the confirmation of the immunoreactivity of the designed MEV in vivo, which will be carried out in the next studies.

## Conclusion

In the present study, we performed meticulous genome analysis of *M. tuberculosis* at the level of proteins and immunogenic epitopes. A MEV was generated by considering the most immunogenic epitopes with particular immunogenic properties, such as high conservation, IFN-γ release, and resemblance to TCR epitopes. To induce a probable mucosal immune response against *M. tuberculosis*, the C-terminal fragment of CPE was fused to the C-terminal of MEV. The designed MEV showed promising interactions with human MHC I and MHC II. It seems that the immunoreactive peptides can open the walls to introduce a promising vaccine against *M. tuberculosis* in mixed or fused formulations. The results presented here suggest that MEV might possess the capacity to function as a nasal vaccine against *M. tuberculosis*, can eliciting immune responses in the mucosal membranes. These results will be evaluated experimentally in the near future.

## Supplementary Information

Supplementary Material 1: Data 1. Screening the surface-exposed proteins of *M. tuberculosis* based on immunogenic properties, such as transmembrane helices, antigenicity, allergenicity, sequence similarity to the human proteome.

Supplementary Material 2: Data 2. Conformational B-cell epitopes of seven immunogenic proteins against *M. tuberculosis*. The detailed information of sequence and color of conformational epitopes are included.

Supplementary Material 3: Data 3. Detailed information of 718 epitopes of *M. tuberculosis* that are involved in experimental immunological assays.

Supplementary Material 4: Data 4. Identification of conserved, antigenic and non-allergen Epitopes IFN-γ releasing epitopes. Out of 460 IFN-γ releasing epitopes, 152 showed a conservancy score > 95%. Subsequently 14 antigenic and non-allergenic epitopes were identified.

Supplementary Material 5: Data 5. The predicted 3D structure of the designed MEV against *M. tuberculosis*.

Supplementary Material 6: Data 6. MHC I and MHC II epitopes of the MEV identified using NetCTL-1.2 and IEDB web tool.

## Data Availability

The data that supports the findings of this study are available in the supplementary material of this article.

## Abbreviations

BCG: Bacille Calmette-Guérin
TST: Tuberculin Skin Tests
IGRA: Interferon Gamma Release Assay
IEDB: Immune Epitope Database and Analysis Resource
CDS: Coding Sequence(s)
CPE: *Clostridium perfringens* Enterotoxin
MEV: Multi-Epitope Vaccine
MDR: Multidrug-Resistant
MHC: Major Histocompatibility Complex
PE/PPE: Proline-glutamic acid / Proline-Proline-glutamic acid family proteins
TB: Tuberculosis
