# Supplementary material for: Design of a multi-epitope vaccine against Mycobacterium tuberculosis using reverse vaccinology and immunoreactive peptides
Source: Genomics Inform. 2026 Jul 8;24:13. doi: 10.1186/s44342-026-00075-6 (PMC13348615; doi:10.1186/s44342-026-00075-6)
Supplement: Supplementary file 2 — Supplementary Material 2: Data 2. Conformational B-cell epitopes of seven immunogenic proteins against M. tuberculosis. The detailed information of sequence and color of conformational epitopes are included. [file 44342_2026_75_MOESM2_ESM.docx]

**Supplementary data 2.** **Conformational B-cell epitopes of seven immunogenic proteins against *M. tuberculosis.*** The detailed information of sequence and color of conformational epitopes are included.

| Protein | Conformational B-cell epitopes | Score | Color |
| --- | --- | --- | --- |
| RipC  WP_003411373.1 | M1,R2,L3,D4,Q5,R6,W7 | 0.93 | Orange |
|  | V106,A107,A108,A109,T110,Y111,M112,G113,G114,R115,T116,H117,G118,M119,D120,A121,I122,L123,T124,A125,E126,S127,P128,Q129,L130,L131,I132,D133,R134,L135,S136,V137,Q138,R139,V140,M141,A142,H143,Q144,M145,S146 | 0.91 | Cyan |
|  | A236,L237,P238,P239,G240,A241,P242,P243,G244,D245,G246,P249 | 0.85 | Red |
|  | L8,I9,A10,R11 | 0.84 | Green |
|  | T214,A217,D218,P219 | 0.82 | Magenta |
|  | G354,D355,G356 | 0.82 | Hot pink |
| BlaC  WP_003410677.1 | M1,N3,G5,F6,G7,R8,E10,L11,L12,V13,A14,M15,A16,M17,L18,V19,S20,V21,T22,G23,C24,A25,R26,H27,A28,S29,G30,A31,R32,P33 | 0.91 | Blue |
|  | K105,L106,I107,T108,Y109,T110,S111,D112,D113,I114,S118,P119,V120,Q122,Q123,H124,V125,Q126,T127,G128,M129,T130 | 0.80 | Purple |
| C40 family peptidase  WP_003901769.1 | R2,G3,S4,S5,E6,Y7,S8,E11,S103,D104,T105,T106,V107,T108,A109,V110,M111,P112,I113,A114,Q115,R116 | 0.90 | Red |
|  | T33,T34,P35,D38,L39,L40,S41,R42,A43,A44,D45,L46,N47,V48,G49,A50,G51,Q52,R53,R54,Y55,Q56 | 0.89 | Blue |
|  | R184,L185,G186,R187,P188,V190,A193,T194,G195,P196,N197,Q198,F199,H262,A263,G264,A265,S266,V267 | 0.80 | Light Magenta |
| MT2404  WP_003412039.1 | G309,N310,P311,A312,D313,P314,G315,N316 | 0.94 | Green |
|  | G13,V14,L15,A16,A17,G18,G19,D20,D21,V22,S23,A24,G25,I26,A27,L29,F30,R33 | 0.88 | Magenta |
|  | L231,M232,P233,G234,F235,G236,G237,A238,P239,V241,G242,G243,H244,W245,G246 | 0.88 | Cyan |
|  | G94,Q95,S96,R97,G98,A99,G100,G101,F102,G103,G104,G109,K111,G112,D114,G115,G116,P117,I118,G119,N120,G121,Q122,V123 | 0.86 | Red |
|  | S134,T135,D136,G137,N138,P139,G140,S153,H154,G155,S156,G157,A158 | 0.82 | Yellow |
|  | G214,D215,G216 | 0.81 | Blue |
| PPE40  WP_003904827.1 | A78,A79,R80,A81,A82,M83,V84,D85,P86,V87,V88,V89,A90,A91,N92,R93,S94,A95,F96,V97,Q98,L99,V100,L101,S102,N103,V104,F105,G106,Q107,N108,A109,P110,A111,I112,A113,A114,A115,E116,A117,T118,Y119,E120,Q121,M122,W123,A124,A125,D126,V127,A128,A129,M130,V131,G132,H134 | 0.93 | Green |
|  | N515,T516,G517,N518,A526,G527,P528,A529,M530,L531,P532,G533,N535,A539,N540,I541,G542,S543,F544,N545,A546,G547,A549,S551,G552,N553,N554,L555,A556,G557,I558,S559,N560,S561,G562,D563,D564,S565,S566,G567,A568,V569,N570,S571,G572,S573,Q574,N575,S576,G577,A578,F579,N580,A581,G582,V583,G584,L585,S586,G587,F588,F589,R590 | 0.84 | Cyan |
|  | N182,L183,G184,V185,G186,N187,I188,G189,G194,S195,G196,N197,G204,S205,G206 | 0.80 | Orange |
| Acr2  WP_003900838.1 | R129,I130,A131,I132,T133,K134 | 0.97 | Orange |
|  | D75,G76,R77,T78,L79,E81 | 0.89 | Cyan |
|  | D70,A71,G72,D73,K74 | 0.88 | Red |
|  | Q126,A127,Q128 | 0.85 |  |
| RipA  WP_003407523.1 | A81,Q83,H84,R85,F86,D87,T88,F89,A90,A91,A92,T93,Y94,M95,N96,G97,P98,S99,V100,S101,Y102,L103,S104,A105,S106,S107,P108,D109,E110,I111,I112,A113,T114,V115,T116,A117,A118,K119,T120,L121,S122,A123,S124,S125,Q126,A127,V128,M129 | 0.92 | Yellow |
|  | D222,P223,G224,S225,G226,P227,A228 | 0.88 | Cyan |
|  | L204,W207,S208,S209,E210,G211,G212,Q213,G214,A215,P216,P217 | 0.87 | Purple |
|  | V1,C2,F218,R219,M220,W221 | 0.86 | Green |
|  | N258,V260,L261,G262,I263,S264,A265 | 0.85 | Orange |
