# Supplementary material for: Design of a multi-epitope vaccine against Mycobacterium tuberculosis using reverse vaccinology and immunoreactive peptides
Source: Genomics Inform. 2026 Jul 8;24:13. doi: 10.1186/s44342-026-00075-6 (PMC13348615; doi:10.1186/s44342-026-00075-6)
Supplement: Supplementary file 3 — Supplementary Material 3: Data 3. Detailed information of 718 epitopes of M. tuberculosis that are involved in experimental immunological assays. [file 44342_2026_75_MOESM3_ESM.docx]

# Supplementary Data 3. Detailed information of 718 epitopes of *M. tuberculosis* that are involved in experimental immunological assays

| **No.** | **Header** | **Peptide** | **T cell assays** | **B cell assays** | **MHC ligand assays** |
| --- | --- | --- | --- | --- | --- |
| **1** | **1** | VLTDWAAPV |  |  | 1 MHC ligand assay |
| **2** | **2** | PVVVAANRSAFVQLV | Activation |  |  |
| **3** | **3** | NLGTGNSGWGNSDPS |  | Qualitative binding |  |
| **4** | **5** | ALAPWQQAV |  |  | 1 MHC ligand assay |
| **5** | **7** | AAAPYAGW | - |  |  |
| **6** | **8** | VVAANRSAF |  |  | 3 MHC ligand assay |
| **7** | **9** | NQGGWMLSRASAMEL | - |  |  |
| **8** | **10** | RPRDRCARI |  |  | 1 MHC ligand assay |
| 9 | 11 | KTQGPGAWPK | IFNg release |  | 1 MHC ligand assay |
| 10 | 12 | NARTTLIAAAIAGTL | IFNg release |  |  |
| 11 | 13 | RTTLIAAAIAGTLVT | IFNg release |  |  |
| **12** | **15** | AWGAYGE | - |  |  |
| **13** | **16** | TLLDYAAGV |  |  | 1 MHC ligand assay |
| **14** | **17** | YQERFVLAL |  |  | 2 MHC ligand assay |
| **15** | **18** | APQLVSTAA |  |  | 2 MHC ligand assay |
| **16** | **19** | VSTAIAALF |  |  | 2 MHC ligand assay |
| **17** | **20** | AEAASATPL |  |  | 3 MHC ligand assay |
| **18** | **21** | EVSTAIAAL |  |  | 2 MHC ligand assay |
| **19** | **22** | AISAQVAAY |  |  | 3 MHC ligand assay |
| **20** | **23** | WPRGAGAAV |  |  | 2 MHC ligand assay |
| **21** | **24** | FCGRIHTRYSSAYEL |  | qualitative binding |  |
| **22** | **25** | AVHDTLFYCV | Cytotoxicity |  | 1 MHC ligand assay |
| **23** | **26** | CVANMPASVPK |  |  | 1 MHC ligand assay |
| **24** | **27** | VSNSLVAHMK |  |  | 1 MHC ligand assay |
| **25** | **28** | RIANGMGATV | Cytotoxicity |  | 1 MHC ligand assay |
| **26** | **29** | ILFTFLHLA | Cytotoxicity |  | 1 MHC ligand assay |
| **27** | **30** | GLSTHEGALL | Cytotoxicity |  | 2 MHC ligand assay |
| **28** | **31** | VLMGGVPGVE | IFNg release  Cytotoxicity  Qualitative binding |  | 2 MHC ligand assay |
| **29** | **32** | TVLDINIDK |  |  | 1 MHC ligand assay |
| **30** | **33** | LLSERVATDL | Cytotoxicity |  | 2 MHC ligand assay |
| **31** | **34** | VPKTSTYAL |  |  | 2 MHC ligand assay |
| **32** | **36** | LLDSGTTSI |  | Qualitative binding | 2 MHC ligand assay |
| **33** | **37** | YLRRRQKAL |  |  | 2 MHC ligand assay |
| **34** | **38** | TTALFGH | - |  |  |
| **35** | **39** | YASVEAANASPLQVA | IFNg release |  |  |
| **36** | **40** | FVQALTTAAASYASV | IFNg release |  |  |
| **37** | **41** | FHSRFVQAL |  |  | 16 MHC ligand assays |
| **38** | **42** | GTRQTLQGASVTVTGQGNSLKVGNAD  VVCGGVSTANATVYMIDSVLMPPA |  | Qualitative binding |  |
| **39** | **43** | TGPASVQGMSQDPVAVAASNNPELTTL  TAALSG |  | Qualitative binding |  |
| **40** | **44** | GDLVGPGCAEYAAANPTGPASVQGM | IFNg release  Proliferation |  |  |
| **41** | **46** | ALSGQLNPQVNLVDTLNSGQYTVFA | IFNg release  Proliferation |  |  |
| **42** | **47** | GNADVVCGGVSTANATVYMIDSVLM | IFNg release  Proliferation |  |  |
| **43** | **48** | PTGPASVQGMSQDPVAVAASNNPEL | IFNg release  Proliferation |  |  |
| **44** | **49** | GASVTVTGQGNSLKVGNADVVCGGV | IFNg release  Proliferation |  |  |
| **45** | **50** | SSLLTSILTYHVVAGQTSPANVVGT | IFNg release  Proliferation |  |  |
| **46** | **51** | QTSPANVVGTRQTLQGASVTVTGQG | IFNg release  Proliferation |  |  |
| **47** | **52** | AVAASNNPELTTLTAALSGQLNPQV | IFNg release  Proliferation |  |  |
| **48** | **54** | LNPQVNLVDTLNSGQYTVFA | IFNg release  Proliferation | Qualitative binding |  |
| **49** | **56** | FSKLPASTIDELKTNSSLLTSILTY | IFNg release  Proliferation |  |  |
| **50** | **57** | YAAANPTGPASVQGMSQDPV | IFNg release  Proliferation | Qualitative binding |  |
| **51** | **58** | VCGGVSTANATVYMIDSVLM | IFNg release  Proliferation | Qualitative binding |  |
| **52** | **59** | PTNAAFSKLPASTIDELKTN | IFNg release  Proliferation | Qualitative binding |  |
| **53** | **60** | GVSTANATVYMIDSVLMPPA | IFNg release  Proliferation | Qualitative binding |  |
| **54** | **61** | NSLKVGNADVVCGGVSTANA | IFNg release  Proliferation | Qualitative binding |  |
| **55** | **64** | IDELKTNSSLLTSILTYHVV | IFNg release |  |  |
| **56** | **66** | MKVKNTIAATSFAAAGLAAL | IFNg release  Proliferation | Qualitative binding |  |
| **57** | **68** | CGGVSTANATVYMID |  | Qualitative binding |  |
| **58** | **69** | ANATVYMIDSVLMPP | IFNg release |  |  |
| **59** | **71** | AAAGDLVGPGCAEYA |  | Qualitative binding |  |
| **60** | **72** | YHVVAGQTSPANVVG |  | Qualitative binding |  |
| **61** | **73** | TNSSLLTSILTYHVVA |  | Proliferation |  |
| **62** | **74** | SPPAAAGDLVGPGCA |  | Qualitative binding |  |
| **63** | **75** | ASVTVTGQGNSLKVGN |  | Proliferation |  |
| **64** | **76** | VTVTGQGNSLKVGNA |  | Qualitative binding |  |
| **65** | **78** | GLAALAVAVSPPAAAGDLVGPGCAE | IFNg release  Proliferation |  |  |
| **66** | **79** | TVYMIDSVLMPPA | IFNg release  Proliferation |  |  |
| **67** | **80** | VNLVDTLNSGQYT | IFNg release |  |  |
| **68** | **81** | AAFSKLPASTIDELK | IFNg release |  |  |
| **69** | **84** | NAAFSKLPAST | IFNg release |  |  |
| **70** | **85** | IDSVLMPPA | proliferation | qualitative binding |  |
| **71** | **86** | YMIDSVLM |  | qualitative binding |  |
| **72** | **87** | GMSQDPVA |  | qualitative binding |  |
| **73** | **88** | NPQVNLVD |  | qualitative binding |  |
| **74** | **89** | PQVNLVDT |  | qualitative binding |  |
| **75** | **90** | NPTGPASV |  | qualitative binding |  |
| **76** | **91** | ANVVGTRQ |  | qualitative binding |  |
| **77** | **92** | AEYAAANP |  | qualitative binding |  |
| **78** | **93** | STANATVY |  | qualitative binding |  |
| **79** | **94** | NVVGTRQ |  | qualitative binding |  |
| **80** | **95** | KTNSSLLT |  | qualitative binding |  |
| **81** | **96** | VPWQPAFVF | IFNg release |  |  |
| **82** | **97** | LLVIPVALSASIIRL | IFNg release |  |  |
| **83** | **98** | YMLDMTFPV |  |  | 9 MHC ligand assay |
| **84** | **99** | VPRSAIPPL |  |  | 2 MHC ligand assay |
| **85** | **100** | PMLVAANRNAFVQLV | Activation |  |  |
| **86** | **101** | VLPPEINSL |  |  | 2 MHC ligand assay |
| **87** | **102** | AAAAPYAGW |  |  | 1 MHC ligand assay |
| **88** | **103** | LPAPPRPTV |  |  | 2 MHC ligand assay |
| **89** | **105** | KRIGASLAH |  |  | 1 MHC ligand assay |
| **90** | **106** | RPLIGNGAN | - |  |  |
| **91** | **107** | DLKRIGASL |  |  | 18 MHC ligand assays |
| **92** | **108** | VSTAVAALF |  |  | 2 MHC ligand assays |
| **93** | **109** | GTGGNASATGT |  |  | 1 MHC ligand assay |
| **94** | **110** | KPRNKGLRF |  |  | 1 MHC ligand assay |
| **95** | **111** | RLFAGLMSY |  |  | 2 MHC ligand assays |
| **96** | **112** | LGRWKWHDPWVHASLLAQNN | IFNg release |  |  |
| **97** | **113** | TRVWVWSPTNPGASDPAAMI | IFNg release |  |  |
| **98** | **114** | PAGGAYSMYTNWEQD |  | qualitative binding |  |
| **99** | **115** | LPDWLAANRGLAPGG |  | qualitative binding |  |
| **100** | **116** | VAFLAGGPHAVYLLD | IFNg release |  |  |
| **101** | **117** | PVAFLAGGPHAVYLL | IFNg release |  |  |
| **102** | **119** | LMVPSPSMGRDIPVAFLAGG | - |  |  |
| **103** | **120** | SPSMGRDIPVAFLA | proliferation |  |  |
| **104** | **122** | GGPHAVYLL | IFNg release  Cytotoxicity  Qualitative binding |  | 1 MHC ligand assay |
| **105** | **123** | FLAGGPHAV |  |  | 2 MHC ligand assay |
| **106** | **124** | TLAGKGISVV | IFNg release  Cytotoxicity  Qualitative binding |  | 1 MHC ligand assay |
| **107** | **125** | LAGKGISVV | IFNg release  Cytotoxicity |  |  |
| **108** | **127** | KAGCQTYKWETFLTSELPGWLQANRHV | IFNg release |  |  |
| **109** | **128** | GLSVVMPVGGQSSFYSDWYQPAC | IFNg release |  |  |
| **110** | **129** | FSRPGLPVEYLQVPSPSMGRDIK | IFNg release |  |  |
| **111** | **130** | LTSELPGWLQANRHVKPTGSAVV | IFNg release |  |  |
| **112** | **131** | DWYQPACGKAGCQTYKWETF | IFNg release  IL-2 release  Proliferation | Qualitative binding |  |
| **113** | **132** | THSWEYWGAQLNAMKPDLQR | IFNg release  IL-2 release  Proliferation |  |  |
| **114** | **133** | NGVFDFPDSGTHSWEYWGAQ | IFNg release  IL-2 release  Proliferation |  |  |
| **115** | **134** | GCQTYKWETFLTSELPGWLQ | IFNg release  IL-2 release  Proliferation |  |  |
| **116** | **135** | MKPDLQRALGATPNTGPAPQGA | IL-2 release  Proliferation |  |  |
| **117** | **136** | GLRAQDDFSGWDINTPAFEW | IL-2 release  Proliferation | Qualitative binding |  |
| **118** | **137** | WDINTPAFEWYDQSGLSVVM | IFNg release  IL-2 release  Proliferation |  |  |
| **119** | **138** | IANNTRVWVYCGNGKPSDLG | IFNg release  IL-2 release  Proliferation |  |  |
| **120** | **139** | QRNDPLLNVGKLIANNTRVW | IFNg release |  |  |
| **121** | **140** | NDPLLNVGKLIANNTRVWVY | IFNg release  IL-2 release  Proliferation |  |  |
| **122** | **141** | PVGGQSSFYSDWYQPACGKA | IFNg release  IL-2 release  Proliferation | Qualitative binding |  |
| **123** | **142** | GNNLPAKFLEGFVRTSNIKF | IFNg release  IL-2 release  Proliferation |  |  |
| **124** | **143** | AGGYKASDMWGPKEDPAWQR | IFNg release  IL-2 release  Proliferation |  |  |
| **125** | **144** | TFLTSELPGWLQANRHVKPT | IFNg release  IL-2 release  pathogen burden after challenge  TNFa release |  |  |
| **126** | **145** | GPKEDPAWQRNDPLLNVGKL | IFNg release  IL-2 release  proliferation |  |  |
| **127** | **148** | PDSGTHSWEYWGAQLNAMK | IFNg release |  |  |
| **128** | **149** | QDAYNAGGGHNGVFDFPDSG | IFNg release  IL-2 release  proliferation |  |  |
| **129** | **150** | KPDLQRALGATPNTGPAPQGA | IFNg release  IL-2 release |  |  |
| **130** | **152** | LAIYHPQQFVYAGAMSGLLD | IFNg release |  |  |
| **131** | **153** | EWYDQSGLSVVMPVGGQSSF | IFNg release  pathogen burden after challenge |  |  |
| **132** | **154** | VPSPSMGRDIKVQFQSGGAN | qualitative binding |  |  |
| **133** | **155** | PTLIGLAMGDAGGYKASDMW | IFNg release  IL-2 release  Proliferation  Cytotoxicity |  |  |
| **134** | **156** | LGGNNLPAKFLEGFVRTSNI | IFNg release |  |  |
| **135** | **157** | SALTLAIYHPQQFVYAGAMS | IFNg release  IL-2 release  proliferation |  |  |
| **136** | **158** | GFVRTSNIKFQDAYNAGGGH | IFNg release  IL-2 release  proliferation |  |  |
| **137** | **159** | LQVPSPSMGRDIKVQFQSGG | IFNg release  IL-2 release  proliferation | Qualitative binding | 1 MHC ligand assay |
| **138** | **160** | DIKVQFQSGGANSPALYLLD | IFNg release  IL-2 release  proliferation |  |  |
| **139** | **161** | ANSPALYLLDGLRAQDDFSG | IL-2 release  proliferation |  |  |
| **140** | **162** | DPAWQRNDPLLNVGKLIAN | IFNg release |  |  |
| **141** | **163** | GNGKPSDLGGNNLPAKFLEG | IL-2 release  proliferation |  |  |
| **142** | **164** | QQFVYAGAMSGLLDPSQAMG | IFNg release  IL-2 release  Proliferation  Cytotoxicity |  |  |
| **143** | **166** | YDQSGLSVVMPVGGQSSFYS | IFNg release  IL-2 release  Proliferation  Cytotoxicity |  |  |
| **144** | **168** | ALYLLDGLRAQDDFSGWDI | IFNg release  IL-2 release  proliferation |  |  |
| **145** | **169** | GLLDPSQAMGPTLIGLAMGD | IFNg release  IL-2 release  proliferation |  |  |
| **146** | **170** | ANRHVKPTGSAVVGLSMAAS | IFNg release  IL-2 release  proliferation |  |  |
| **147** | **171** | YLQVPSPSMGRDIKVQFQ | IFNg release  IL-2 release  proliferation |  |  |
| **148** | **172** | LQANRHVKPTGSAVVGLSM | IFNg release |  |  |
| **149** | **173** | GKAGCQTYKWETFLTSE | Qualitative binding |  |  |
| **150** | **175** | AVVGLSMAASSALTLAIYHP | IFNg release  IL-2 release  proliferation |  |  |
| **151** | **176** | VFDFPDSGTHSWEYW | IFNg release |  |  |
| **152** | **177** | ACGKAGCQTYKWETF | IFNg release |  |  |
| **153** | **178** | FYSDWYQPACGKAGC | IFNg release |  |  |
| **154** | **179** | FSGWDINTPAFEWYD | IFNg release |  |  |
| **155** | **180** | AGCQTYKWETFLTSE | IFNg release |  | 3 MHC ligand assay |
| **156** | **181** | GKLIANNTRVWVYCG | IFNg release |  |  |
| **157** | **183** | KEDPAWQRNDPLLNV | IFNg release |  |  |
| **158** | **184** | NNTRVWVYCGNGKPS | IFNg release |  |  |
| **159** | **186** | DSGTHSWEYWGAQLN | IFNg release |  |  |
| **160** | **188** | SWEYWGAQLNAMKPD | IFNg release | Qualitative binding |  |
| **161** | **189** | GGQSSFYSDWYQPAC | IFNg release |  |  |
| **162** | **190** | SELPGWLQANRHVKP | IFNg release |  |  |
| **163** | **191** | FLEGFVRTSNIKFQD | IFNg release |  |  |
| **164** | **192** | PGWLQANRHVKPTGS | IFNg release |  |  |
| **165** | **193** | LTLAIYHPQQFVYAG | IFNg release |  |  |
| **166** | **194** | KWETFLTSELPGWLQ | IFNg release |  |  |
| **167** | **195** | RAQDDFSGWDINTPA | IFNg release |  |  |
| **168** | **196** | QTYKWETFLTSELPG | IFNg release |  |  |
| **169** | **198** | PAFEWYDQSGLSVVM | IFNg release |  |  |
| **170** | **199** | VMPVGGQSSFYSDWY | IFNg release | Qualitative binding | 3 MHC ligand assay |
| **171** | **200** | FEWYDQSGLSVVMPV | IFNg release |  |  |
| **172** | **201** | GGHNGVFDFPDSGTH | IFNg release |  |  |
| **173** | **202** | WLQANRHVKPTGSAV | IFNg release |  |  |
| **174** | **203** | WQRNDPLLNVGKLIA | IFNg release |  |  |
| **175** | **204** | AYNAGGGHNGVFDFP | IFNg release |  |  |
| **176** | **205** | YHPQQFVYAGAMSGL | IFNg release |  |  |
| **177** | **206** | PVEYLQVPSPSMGRD | IFNg release |  |  |
| **178** | **207** | AIYHPQQFVYAGAMS | IFNg release |  |  |
| **179** | **209** | VEYLQVPSPSMGRDI | IFNg release |  |  |
| **180** | **210** | VVMPVGGQSSFYSDW | IFNg release | Qualitative binding |  |
| **181** | **211** | ETFLTSELPGWLQAN | IFNg release |  |  |
| **182** | **213** | AFSRPGLPVEYLQVP |  |  | 3 MHC ligand assay |
| **183** | **214** | PLLNVGKLIANNTRV | IFNg release |  |  |
| **184** | **215** | LPVEYLQVPSPSMGR | IFNg release  Proliferation |  | 3 MHC ligand assay |
| **185** | **217** | AGGGHNGVFDFPDSG |  | Qualitative binding |  |
| **186** | **218** | LLDGLRAQDDFSGWD | IFNg release |  |  |
| **187** | **220** | PSPSMGRDIKVQFQS | IFNg release  Activation |  |  |
| **188** | **221** | ATAGAFSRPGLPVEY | IFNg release  proliferation |  |  |
| **189** | **223** | KVQFQSGGANSPALY | IFNg release |  |  |
| **190** | **226** | QFVYAGAMSGLLDPS | proliferation |  |  |
| **191** | **227** | FVYAGAMSGLLDPSQ | IFNg release |  |  |
| **192** | **228** | FQSGGANSPALYLLD | IFNg release  IL-2 release  Degranulation  TNF-a release |  |  |
| **193** | **229** | GAQLNAMKPDLQRAL | IFNg release |  |  |
| **194** | **230** | SPALYLLDGLRAQDD | IFNg release |  |  |
| **195** | **231** | PSDLGGNNLPAKFLE |  | qualitative binding |  |
| **196** | **233** | AMSGLLDPSQAMGPT | IFNg release |  |  |
| **197** | **235** | MGRDIKVQFQSGGAN | IFNg release  Activation |  |  |
| **198** | **236** | PSMGRDIKVQFQSGG | IFNg release  IL-2 release  Activation  TNF-a release  proliferation |  |  |
| **199** | **237** | LSVVMPVGGQSSFYS | IFNg release  IL-2 release  TNF-a release  Degranulation |  |  |
| **200** | **238** | SPSMGRDIKVQFQS | proliferation |  |  |
| **201** | **239** | ALGATPNTGPAPQGA | IFNg release |  |  |
| **202** | **240** | AMGPTLIGLAMGDAG | IFNg release |  |  |
| **203** | **241** | SMAASSALTLAIYHP | IFNg release |  |  |
| **204** | **242** | QSGLSVVMPVGGQSS | IFNg release | Qualitative binding |  |
| **205** | **244** | RPATKEQPF |  |  | 1 MHC ligand assay |
| **206** | **245** | DMWEHAFYLQ | cytotoxicity |  | 2 MHC ligand assay |
| **207** | **247** | QINELHHSK |  |  | 18 MHC ligand assays |
| **208** | **248** | GNFERISGDLKTQIDQVESTAGSLQ | proliferation |  |  |
| **209** | **249** | MAEMKTDAATLAQEAGNFERISGDL | proliferation |  |  |
| **210** | **250** | AANKQKQELDEISTNIRQAGVQYSR | proliferation |  |  |
| **211** | **251** | IRQAGVQYSRADEEQQQALSSQMGF | proliferation |  |  |
| **212** | **252** | RFQEAANKQKQELDEISTNIRQA |  | qualitative binding |  |
| **213** | **253** | LAQEAGNFERISGDLKTQID | IFNg release  Proliferation  Il-4 release |  |  |
| **214** | **254** | QKQELDEISTNIRQAGVQYS | IFNg release |  |  |
| **215** | **255** | EISTNIRQAGVQYSRADEEQ | IFNg release  granzyme A release  perforin release  proliferation  cytotoxicity  IL-4 release  granulysin release  granzyme B release |  |  |
| **216** | **257** | VQYSRADEEQQQALSSQMGF | IFNg release  granzyme A release  perforin release  proliferation  cytotoxicity  IL-4 release  granulysin release  granzyme B release |  |  |
| **217** | **258** | LDEISTNIRQAGVQYSRAD | IFNg release |  |  |
| **218** | **260** | QVESTAGSLQGQWRGAAGTA | IFNg release  proliferation  IL-4 release |  |  |
| **219** | **261** | ISGDLKTQIDQVESTAGSLQ | IFNg release  proliferation  IL-4 release |  |  |
| **220** | **262** | AQAAVVRFQEAANKQKQELD | IFNg release  granzyme A release  perforin release  proliferation  cytotoxicity  granulysin release  granzyme B release |  |  |
| **221** | **263** | ELDEISTNIRQAGVQYSR | IFNg release |  |  |
| **222** | **264** | ATLAQEAGNFERISGDLK | IFNg release |  |  |
| **223** | **265** | DQVESTAGSLQGQWRGAA | IFNg release  IL-2 release  TNF-a release |  |  |
| **224** | **267** | VVRFQEAANKQKQELDEI | IFNg release  proliferation |  |  |
| **225** | **268** | NIRQAGVQYSRADEEQQQ | IFNg release  proliferation |  |  |
| **226** | **270** | NFERISGDLKTQIDQV | IFNg release |  |  |
| **227** | **271** | GDLKTQIDQVESTAGSL | IFNg release |  |  |
| **228** | **272** | QEAGNFERISGDLKTQ | IFNg release  Proliferation |  |  |
| **229** | **273** | IDQVESTAGSLQGQWR | IFNg release |  |  |
| **230** | **274** | AGTAAQAAVVRFQEAANK | IFNg release |  |  |
| **231** | **275** | SRADEEQQQALSSQMGF | IFNg release |  |  |
| **232** | **277** | AAVVRFQEAANKQKQEL | IFNg release  IL-2 release  TNF-a release |  |  |
| **233** | **282** | TNIRQAGVQYSRADE | IFNg release |  |  |
| **234** | **284** | RADEEQQQALSSQMGF | IFNg release  Proliferation |  |  |
| **235** | **285** | DEISTNIRQAGVQYS | IFNg release |  |  |
| **236** | **288** | STNIRQAGVQYSRAD | IFNg release  Cytotoxicity |  |  |
| **237** | **291** | KQELDEISTNIRQAG | IFNg release | Qualitative binding | 26 MHC ligand assay |
| **238** | **292** | ISTNIRQAGVQYSRA | IFNg release |  | 45 MHC ligand assay |
| **239** | **293** | ERISGDLKTQIDQVE | IFNg release |  |  |
| **240** | **294** | QELDEISTNIRQAGV | IFNg release |  |  |
| **241** | **296** | DAATLAQEAGNFERI | IFNg release |  |  |
| **242** | **298** | TAGSLQGQWRGAAGT | IFNg release |  |  |
| **243** | **299** | TDAATLAQEAGNFER | IFNg release  IL-2 release | Qualitative binding | 26 MHC ligand assay |
| **244** | **300** | VRFQEAANKQKQELD | IFNg release | Qualitative binding | 26 MHC ligand assay |
| **245** | **301** | STAGSLQGQWRGAAG | IFNg release |  |  |
| **246** | **302** | KTDAATLAQEAGNFE | IFNg release  IL-2 release  TNF-a release |  |  |
| **247** | **305** | RQAGVQYSRADEEQQ | IFNg release |  |  |
| **248** | **306** | FQEAANKQKQELDEI | IFNg release |  |  |
| **249** | **307** | GSLQGQWRGAAGTAA | IFNg release |  |  |
| **250** | **308** | AGSLQGQWRGAAGTA | IFNg release | Qualitative binding | 26 MHC ligand assay |
| **251** | **311** | SLQGQWRGAAGTAAQ | IFNg release |  |  |
| **252** | **312** | LKTQIDQVESTAGSL | IFNg release |  |  |
| **253** | **314** | LQGQWRGAAGTAAQA | IFNg release  IL-2 release  IL-10 release  Proliferation  TNFa release |  |  |
| **254** | **315** | KTQIDQVESTAGSLQ | IFNg release | Qualitative binding | 26 MHC ligand assay |
| **255** | **316** | ADEEQQQALSSQMGF | IFNg release | Qualitative binding | 26 MHC ligand assay |
| **256** | **317** | AGVQYSRADEEQQQA | IFNg release |  |  |
| **257** | **318** | AVVRFQEAANKQKQE | IFNg release |  |  |
| **258** | **321** | QAAVVRFQEAANKQK | IFNg release  qualitative binding  activation  IL-17 release |  | 40 MHC ligand assays |
| **259** | **325** | AAQAAVVRFQEAANK | IFNg release |  |  |
| **260** | **326** | TAAQAAVVRFQEAAN | IFNg release  Activation |  |  |
| **261** | **328** | GTAAQAAVVRFQEAA | IFNg release |  |  |
| **262** | **336** | TQIDQVESTAGSL | IFNg release |  |  |
| **263** | **337** | EEQQQALSSQMGF | IFNg release |  |  |
| **264** | **339** | AEMKTDAATLA | IFNg release |  | 2 MHC ligand assay |
| **265** | **340** | QWRGAAGTAA | IFNg release |  | 3 MHC ligand assay |
| **266** | **342** | GQWRGAAGTAAQAAVVRFQE | IFNg release  Proliferation  IL-4 release |  |  |
| **267** | **343** | QYSRADEEQQ | IFNg release |  | 3 MHC ligand assay |
| **268** | **344** | QQALSSQMGF | IFNg release |  | 3 MHC ligand assay |
| **269** | **354** | EMKTDAATL | IFNg release  Proliferation  TNF-a release |  | 11 MHC ligand assays |
| **270** | **357** | VESTAGSL | IFNg release  Cytotoxicity  Qualitative binding |  |  |
| **271** | **359** | SGSEAYQGVQQKWDATATELNNALQ | IFNg release  Proliferation |  |  |
| **272** | **360** | QQWNFAGIEAAASAIQGNVTSIHSL | IFNg release | Qualitative binding |  |
| **273** | **361** | SAIQGNVTSIHSLLDEGKQSLTKLA | IFNg release  Proliferation |  |  |
| **274** | **362** | MTEQQWNFAGIEAAASAIQGNVTSI | IFNg release  Proliferation |  |  |
| **275** | **363** | AWGGSGSEAYQGVQQKWDATATEL | IFNg release  Proliferation | Qualitative binding |  |
| **276** | **364** | EGKQSLTKLAAAWGGSGSEAYQGVQ | IFNg release  Proliferation |  |  |
| **277** | **365** | VTSIHSLLDEGKQSLTKLAAAWGG | IFNg release  Proliferation | Qualitative binding |  |
| **278** | **366** | QGVQQKWDATATELNNALQNLART | IFNg release  Proliferation | Qualitative binding |  |
| **279** | **367** | TATELNNALQNLARTISEAGQAMAS | IFNg release  Proliferation |  |  |
| **280** | **368** | LARTISEAGQAMASTEGNVTGMFA | IFNg release  Proliferation | Qualitative binding |  |
| **281** | **369** | EAAASAIQGNVTSIHSLLDEGKQS | IFNg release  Proliferation | Qualitative binding |  |
| **282** | **370** | GKQSLTKLAAAWGGSGSEAYQGVQ | IFNg release  Proliferation | Qualitative binding |  |
| **283** | **371** | WNFAGIEAAASAIQGNVTSIHSL | IFNg release |  |  |
| **284** | **372** | AAAWGGSGSEAYQGVQQKWDATA |  | Qualitative binding |  |
| **285** | **373** | ATELNNALQNLARTISEAGQAMAS | IFNg release  Proliferation | Qualitative binding |  |
| **286** | **374** | LQNLARTISEAGQAMASTEGNVT | IFNg release |  |  |
| **287** | **375** | AAWGGSGSEAYQGVQQKWDATA |  | Qualitative binding |  |
| **288** | **376** | YQGVQQKWDATATELNNALQ | IFNg release  granzyme A release  perforin release  proliferation  cytotoxicity  IL-4 release  granulysin release  granzyme B release  pathogen burden after challenge | Qualitative binding |  |
| **289** | **378** | GIEAAASAIQGNVTSIHSLLD | IFNg release  Proliferation |  |  |
| **290** | **379** | NVTSIHSLLDEGKQSLTKLA | IFNg release  Proliferation |  |  |
| **291** | **381** | ISEAGQAMASTEGNVTGMFA | IFNg release  Proliferation  IL-4 release |  |  |
| **292** | **382** | NLARTISEAGQAMASTEGNV | IFNg release  granzyme A release  perforin release  proliferation  granzyme B release  IL-4 release  granulysin release |  |  |
| **293** | **383** | IEAAASAIQGNVTSIHSLLD | IFNg release  proliferation  IL-4 release |  |  |
| **294** | **386** | LNNALQNLARTISEAGQAM | IFNg release |  |  |
| **295** | **390** | SIHSLLDEGKQSLTKLAA | IFNg release |  |  |
| **296** | **391** | AIQGNVTSIHSLLDEGK | IFNg release |  |  |
| **297** | **392** | EAYQGVQQKWDATATEL | IFNg release |  |  |
| **298** | **393** | NALQNLARTISEAGQAMA | IFNg release |  |  |
| **299** | **397** | VQQKWDATATELNNAL | IFNg release |  |  |
| **300** | **399** | KWDATATELNNALQNL | IFNg release |  |  |
| **301** | **400** | IHSLLDEGKQSLTKLA | IFNg release |  |  |
| **302** | **401** | ATATELNNALQNLARTI | IFNg release |  |  |
| **303** | **402** | LDEGKQSLTKLAAAWG | IFNg release  Proliferation  IL-4 release  IL-10 release | Qualitative binding |  |
| **304** | **404** | QGNVTSIHSLLDEGK | IFNg release  Qualitative binding |  |  |
| **305** | **405** | GNVTSIHSLLDEGKQ | IFNg release |  |  |
| **306** | **406** | GSGSEAYQGVQQKWD | IFNg release  proliferation |  |  |
| **307** | **407** | GVQQKWDATATELNN | IFNg release  proliferation |  |  |
| **308** | **409** | IQGNVTSIHSLLDEG | IFNg release  Qualitative binding |  | 35 MHC ligand assays |
| **309** | **415** | WGGSGSEAYQGVQQK | IFNg release |  |  |
| **310** | **417** | QQKWDATATELNNAL | IFNg release  proliferation |  |  |
| **311** | **418** | QKWDATATELNNALQ | IFNg release |  | 26 MHC ligand assays |
| **312** | **419** | TISEAGQAMASTEGNV | IFNg release |  |  |
| **313** | **420** | TSIHSLLDEGKQSLT | IFNg release |  |  |
| **314** | **421** | KQSLTKLAAAWGGSGS | IFNg release |  |  |
| **315** | **423** | HSLLDEGKQSLTKLA | IFNg release |  | 26 MHC ligand assays |
| **316** | **424** | ARTISEAGQAMASTEG | IFNg release |  |  |
| **317** | **425** | TEQQWNFAGIEAAAS | IFNg release |  |  |
| **318** | **426** | QAMASTEGNVTGMFA | IFNg release  Proliferation |  | 26 MHC ligand assays |
| **319** | **427** | AAASAIQGNVTSIHSL | IFNg release  Proliferation  IL-4 release  IL-10 release | qualitative binding |  |
| **320** | **431** | TKLAAAWGGSGSEAY | IFNg release  Proliferation |  |  |
| **321** | **434** | ELNNALQNLARTISE | IFNg release |  | 35 MHC ligand assays |
| **322** | **436** | QWNFAGIEAAASAIQ | IFNg release  Proliferation |  |  |
| **323** | **438** | NNALQNLARTISEAG | IFNg release  Il-2 release |  | 26 MHC ligand assays |
| **324** | **440** | EQQWNFAGIEAAASA | IFNg release  qualitative binding  proliferation  IL-17 release |  | 38 MHC ligand assays |
| **325** | **441** | TELNNALQNLARTIS | IFNg release |  |  |
| **326** | **443** | QNLARTISEAGQAMA | IFNg release |  | 34 MHC ligand assays |
| **327** | **444** | ASAIQGNVTSIHSLL | IFNg release  Proliferation |  |  |
| **328** | **446** | AGQAMASTEGNVTGM | IFNg release |  |  |
| **329** | **448** | RTISEAGQAMASTEG | IFNg release |  |  |
| **330** | **452** | EAGQAMASTEGNVTG | IFNg release |  |  |
| **331** | **455** | AASAIQGNVTSIHSL | IFNg release |  |  |
| **332** | **456** | AGIEAAASAIQGNVTS | IFNg release |  |  |
| **333** | **458** | ALQNLARTISEAGQA | IFNg release |  |  |
| **334** | **459** | SEAGQAMASTEGNVT | IFNg release  proliferation |  |  |
| **335** | **460** | NFAGIEAAASAIQGN | IFNg release |  |  |
| **336** | **461** | LTKLAAAWGGSGSEA | IFNg release |  | 26 MHC ligand assay |
| **337** | **465** | FAGIEAAASAIQGNV | IFNg release | Qualitative binding |  |
| **338** | **476** | SEAYQGVQQKW | IFNg release |  |  |
| **339** | **477** | STEGNVTGMFA | IFNg release  proliferation  IL-4 release  IL-10 release | Qualitative binding | 1 MHC ligand assay |
| **340** | **485** | ASTEGNVTGM |  |  | 1 MHC ligand assay |
| **341** | **488** | AYQGVQQKW | IFNg release  qualitative binding |  | 13 MHC ligand assay |
| **342** | **501** | DEGKQSLTK |  | Qualitative binding | 7 MHC ligand assays |
| **343** | **509** | KLAAAWGGS |  |  | 10 MHC ligand assays |
| **344** | **510** | AMASTEGNV | IFNg release  qualitative binding |  | 13 MHC ligand assay |
| **345** | **517** | LAAAWGGSG |  |  | 12 MHC ligand assay |
| **346** | **521** | SLTKLAAAW |  |  | 10 MHC ligand assays |
| **347** | **523** | SLLDEGKQSL |  |  | 2 MHC ligand assay |
| **348** | **526** | LLDEGKQSL | IFNg release  qualitative binding  GM-CSF release |  | 16 MHC ligand assays |
| **349** | **533** | MHRIFLITVALALLTASPASAIT | IFNg release |  |  |
| **350** | **534** | GSIRSLARAVVHAANLGVGV | IFNg release |  |  |
| **351** | **535** | AQIIHRITATARHPG | IFNg release |  |  |
| **352** | **536** | APYNVRRLPPPVVEP | IFNg release | Qualitative binding |  |
| **353** | **537** | DDLVGAGVIDAVA | IFNg release | Qualitative binding |  |
| **354** | **538** | APAENIVAL |  |  | 2 MHC ligand assay |
| **355** | **539** | RARRHLAAL |  |  | 9 MHC ligand assay |
| **356** | **540** | NGVFNFPPNGTHSWPYWNEQ | IFNg release  IL-2 release |  |  |
| **357** | **541** | THSWPYWNEQLVAMKADIQH | IFNg release  IL-2 release |  |  |
| **358** | **542** | QNYTYKWETFLTREMPAWLQ | IFNg release  IL-2 release |  |  |
| **359** | **543** | DWYQPSQSNGQNYTYKWETF | IFNg release  IL-2 release |  |  |
| **360** | **544** | WDINTPAFEEYYQSGLSVIM | IFNg release  IL-2 release |  |  |
| **361** | **545** | GFLNPSEGWWPTLIGLAMND | IFNg release  IL-2 release |  |  |
| **362** | **546** | NDPMVQIPRLVANNTRIWVY | IFNg release  IL-2 release |  |  |
| **363** | **547** | QQFPYAASLSGFLNPSEGWW | IFNg release  IL-2 release |  |  |
| **364** | **548** | RDTYAADGGRNGVFNFPPNG | IFNg release  IL-2 release |  |  |
| **365** | **549** | SGGYNANSMWGPSSDPAWKR | IFNg release  IL-2 release |  |  |
| **366** | **550** | LTREMPAWLQANKGVSPTGN | IFNg release  IL-2 release |  |  |
| **367** | **551** | PVGGQSSFYTDWYQPSQSNG | IFNg release  IL-2 release |  |  |
| **368** | **553** | FSRPGLPVEYLQVPSASMGR | IFNg release  IL-2 release |  |  |
| **369** | **554** | YLLDGLRAQDDYNGWDIN | IFNg release  IL-2 release  Proliferation |  |  |
| **370** | **555** | YYQSGLSVIMPVGGQSSFYT | IFNg release  IL-2 release |  |  |
| **371** | **556** | DIKVQFQGGGPHAVYLLD | IFNg release  IL-2 release |  |  |
| **372** | **557** | ANKGVSPTGNAAVGLSMSGG | IFNg release  IL-2 release |  |  |
| **373** | **558** | LDGLRAQDDYNGWDI |  |  | 3 MHC ligand assay |
| **374** | **559** | QDDYNGWDINTPAFE |  |  | 3 MHC ligand assay |
| **375** | **560** | GQNYTYKWETFLTRE | IFNg release |  |  |
| **376** | **561** | AVYLLDGLRAQDDYNG | - |  |  |
| **377** | **562** | VYLLDGLRAQDDYNG | IFNg release  Proliferation |  | 3 MHC ligand assays |
| **378** | **563** | EGWWPTLIGLAMNDS | IFNg release  Proliferation  IL-4 release  pathogen burden after challenge  type IV hypersensitivity (DTH) |  |  |
| **379** | **564** | DPMVQIPRLVANNTR | IFNg release |  |  |
| **380** | **567** | QVPSASMGRDIKVQF | IFNg release  Activation |  |  |
| **381** | **570** | LLDGLRAQDDYNGW |  | qualitative binding |  |
| **382** | **571** | SASMGRDIKVQFQG | proliferation |  |  |
| **383** | **573** | DGLRAQDDYNGW |  | qualitative binding  dissociation constant KD |  |
| **384** | **574** | MTFFEQVRRLR | Cytotoxicity  pathogen burden after challenge |  |  |
| **385** | **582** | RGEHRDEHTQDAGDK |  | qualitative binding |  |
| **386** | **583** | IPVVSVTKSVGFQLRGQSGPTTVK | IFNg release | qualitative binding |  |
| **387** | **584** | AGIERTFVAYLKMAGKTAQDT | IFNg release | qualitative binding |  |
| **388** | **585** | FLTTGCIRWST | - |  |  |
| **389** | **586** | MVNKSRMMPAV | IFNg release | qualitative binding |  |
| **390** | **587** | VMADRTRHL | proliferation |  | 1 MHC ligand assay |
| **391** | **588** | QSFEEVSAR |  |  | 8 MHC ligand assays |
| **392** | **589** | KLVGIELPK |  |  | 21 MHC ligand assays |
| **393** | **590** | RVEESRARL |  |  | 18 MHC ligand assays |
| **394** | **591** | APAKKAAPA |  |  | 21 MHC ligand assays |
| **395** | **593** | AKLVGIELP | - |  |  |
| **396** | **595** | APAKKAAAK |  |  | 22 MHC ligand assay |
| **397** | **597** | GPASLPTAL |  |  | 22 MHC ligand assay |
| **398** | **598** | VVHPAVVQANRVRTWLLAVSNVFGQ | IFNg release | qualitative binding |  |
| **399** | **599** | GQSVTGYNNSVSVTS | IFNg release | qualitative binding |  |
| **400** | **600** | GIVALIALGILEHFD | IFNg release |  |  |
| **401** | **601** | YTKKLWQAIRAQDVC | IFNg release |  |  |
| **402** | **602** | TKKLWQAIRAQDVCG | IFNg release |  |  |
| **403** | **603** | AYTKKLWQAIRAQDV | IFNg release |  |  |
| **404** | **604** | TTSNVSVAK | IFNg release | proliferation | 1 MHC ligand assays |
| **405** | **605** | LAPPPADPA |  |  | 1 MHC ligand assays |
| **406** | **606** | FLTQNFGPV |  |  | 1 MHC ligand assays |
| **407** | **607** | QPAPQGLPI |  |  | 1 MHC ligand assays |
| **408** | **608** | MAAVRTTAL |  |  | 7 MHC ligand assays |
| **409** | **609** | VPAGTTISV |  |  | 2 MHC ligand assays |
| **410** | **610** | GGLVRTVHLPAPNVA | IFNg release  IL-2 release  TNFa release |  |  |
| **411** | **611** | AGVQVHDADTIVLRR | IFNg release  IL-2 release  TNFa release |  |  |
| **412** | **612** | TVHLPAPNVAGLLSA | IFNg release |  |  |
| **413** | **613** | LPVANVVVTPAHEAV | IFNg release  IL-2 release  TNFa release |  |  |
| **414** | **614** | TAMRVTTMK | IFNg release |  | 1 MHC ligand assays |
| **415** | **615** | DRDDLY | - |  |  |
| **416** | **616** | ATLFGSHAR | 2 MHC ligand assays |  |  |
| **417** | **617** | AGANGVAVGAP | 1 MHC ligand assays |  |  |
| **418** | **619** | HVTLYLGNGQM | - |  |  |
| **419** | **620** | MSFVTTQPEALAAAAGSLQGI | IFNg release |  |  |
| **420** | **621** | AAIHEMFVNTLQMSS | IFNg release |  |  |
| **421** | **622** | TGVVPAAADEVSALT | IFNg release |  |  |
| **422** | **624** | PTTGVVPAAADEVS | - |  |  |
| **423** | **625** | AQIYQAVSAQAAAIH | IFNg release  IL-2 release  TNFa release |  |  |
| **424** | **626** | ILIGNGGAGG | - |  |  |
| **425** | **627** | ASYYEVKFSDPSKPNGQIWT | proliferation |  |  |
| **426** | **628** | TDSKAAARLGSDMGEFYMPY | proliferation |  |  |
| **427** | **629** | PDAGPPQRWFVVWLGTANNP | - |  |  |
| **428** | **630** | VRIDNPVGGFSFALPAGWVE | proliferation |  |  |
| **429** | **631** | SLDANGVSGSASYYEVKFSD | proliferation |  |  |
| **430** | **632** | NDTRIVLGRLDQKLYASAEA | proliferation |  |  |
| **431** | **633** | PGTRINQETVSLDANGVSGS | IFNg release  IL-2 release  TNFa release  IL-10 release  IL-2 release  CCL4/MIP-1b release |  |  |
| **432** | **634** | GVIGSPAANAPDAGPPQRWF | proliferation |  |  |
| **433** | **635** | PFPGQPPPVANDTRIVLGRL | proliferation |  |  |
| **434** | **636** | PSKPNGQIWTGVIGSPAANA | proliferation |  |  |
| **435** | **637** | VVWLGTANNPVDKGAAKALA | proliferation |  |  |
| **436** | **638** | PVIAPNAPQPVRIDNPVGGF | proliferation |  |  |
| **437** | **639** | DQKLYASAEATDSKAAARLG | proliferation |  |  |
| **438** | **640** | SDMGEFYMPYPGTRINQETV | proliferation |  |  |
| **439** | **641** | MHQVDPNLTRRKGRLAALAI | proliferation |  |  |
| **440** | **642** | VDKGAAKALAESIRPLVAPP | proliferation |  |  |
| **441** | **643** | PPPAAANTPNAQPGDPNAAP | proliferation |  |  |
| **442** | **644** | VAVPATANADPEPAPPVPTT | proliferation |  |  |
| **443** | **645** | AAMASASLVTVAVPATANAD | IFNg release  IL-2 release  TNFa release  IL-10 release  IL-2 release  CCL4/MIP-1b release |  |  |
| **444** | **646** | AGPPQRWFVVWLGTA |  | qualitative binding |  |
| **445** | **647** | KFSDPSKPNGQIWTGVIG | proliferation |  |  |
| **446** | **648** | APPPPVIAPNAPQPV |  | qualitative binding |  |
| **447** | **649** | MPYPGTRIN | cytotoxicity |  |  |
| **448** | **650** | GWVESDAAH | cytotoxicity |  |  |
| **449** | **651** | WLGTANNPV | cytotoxicity |  |  |
| **450** | **652** | RLDQKLYAS | cytotoxicity |  |  |
| **451** | **653** | RKGRLAALAIA | cytotoxicity |  |  |
| **452** | **654** | QPVRIDNPV | cytotoxicity |  |  |
| **453** | **655** | TTGDPPFPG | cytotoxicity |  |  |
| **454** | **656** | VLGRLDQKL | cytotoxicity |  | 2 MHC ligand assay |
| **455** | **657** | APQPVRIDN | cytotoxicity |  |  |
| **456** | **658** | APAPAPAGEVAPTPTTPTPQ | cytotoxicity |  |  |
| **457** | **659** | KGRLAALAI | cytotoxicity |  |  |
| **458** | **661** | RTVSLPVGA | IFNg release  proliferation |  | 1 MHC ligand assays |
| **459** | **663** | AFPSFAGL | IFNg release  Cytotoxicity  IL-2 release  qualitative binding |  | 2 MHC ligand assay |
| **460** | **664** | SLFPEFSEL | IFNg release |  | 2 MHC ligand assay |
| **461** | **665** | GILTVSVAV | IFNg release  Cytotoxicity  qualitative binding  TNFa release |  |  |
| **462** | **666** | LFPEFSEL | cytotoxicity |  | 2 MHC ligand assay |
| **463** | **667** | SAFGGGNAF |  |  | 4 MHC ligand assay |
| **464** | **668** | ARVIMRSAIG | IFNg release |  |  |
| **465** | **670** | MASASLVTV | cytotoxicity |  |  |
| **466** | **671** | RPLVAPPPA | cytotoxicity |  |  |
| **467** | **672** | PNAPPPPVI | cytotoxicity |  |  |
| **468** | **673** | NAPPPPVIA | cytotoxicity |  |  |
| **469** | **674** | AMASASLVT | cytotoxicity |  |  |
| **470** | **675** | YMPYPGTRI | cytotoxicity |  |  |
| **471** | **676** | SPAANAPDA | cytotoxicity |  |  |
| **472** | **677** | SASLVTVAV | cytotoxicity |  |  |
| **473** | **678** | LVAPPPAPA | cytotoxicity |  |  |
| **474** | **680** | APPAPATPV | cytotoxicity |  |  |
| **475** | **681** | SSMTRIAK |  |  | 1 MHC assay |
| **476** | **682** | MTDVSRKIRAWGRRLMIGTAAAVVLPGLV | - |  |  |
| **477** | **683** | LSMAGSSAMILAAYHPQQFIYAGSLSAL | proliferation |  |  |
| **478** | **685** | DWYSPACGKAGCQTYKWETF | proliferation | qualitative binding | 1 MHC ligand assay |
| **479** | **686** | VFNFPPNGTHSWEYWGAQLN | IFNg release |  |  |
| **480** | **687** | NAVFNFPPNGTHSWEYWGAQ | proliferation | qualitative binding | 1 MHC ligand assay |
| **481** | **688** | GLRAQDDYNGWDINTPAFEW | proliferation | qualitative binding | 1 MHC ligand assay |
| **482** | **689** | PTQQIPKLVANNTRLWVYCG | IFNg release |  |  |
| **483** | **690** | FQDAYNAAGGHNAVFNFPPNG | IFNg release  IL-2 release |  |  |
| **484** | **691** | GCQTYKWETFLTSELPQWLS | proliferation | qualitative binding | 1 MHC ligand assay |
| **485** | **692** | WDINTPAFEWYYQSGLSIVM | IFNg release  Proliferation | qualitative binding | 1 MHC ligand assays |
| **486** | **693** | NIPAEFLENFVRSSNLKFQD | IFNg release |  |  |
| **487** | **694** | AEFLENFVRSSNLKFQDAYN | IFNg release |  |  |
| **488** | **695** | THSWEYWGAQLNAMKGDLQS | IFNg release  Proliferation | qualitative binding | 1 MHC ligand assays |
| **489** | **696** | NDPTQQIPKLVANNTRLWVY | proliferation | qualitative binding | 1 MHC ligand assay |
| **490** | **697** | GYKAADMWGPSSDPAWERND | IFNg release |  |  |
| **491** | **698** | NNTRLWVYCGNGTPNELGGA | IFNg release |  |  |
| **492** | **699** | GPSSDPAWERNDPTQQIPKL | proliferation | qualitative binding | 1 MHC ligand assay |
| **493** | **700** | PVGGQSSFYSDWYSPACGKA | proliferation | qualitative binding | 1 MHC ligand assay |
| **494** | **701** | VANNTRLWVYCGNGTPNELG | proliferation | qualitative binding | 1 MHC ligand assay |
| **495** | **703** | GANIPAEFLENFVRSSNLKF | proliferation | qualitative binding | 1 MHC ligand assay |
| **496** | **704** | AGGYKAADMWGPSSDPAWER | proliferation | qualitative binding | 1 MHC ligand assay |
| **497** | **705** | NFVRSSNLKFQDAYNAAGGH | proliferation | qualitative binding | 1 MHC ligand assay |
| **498** | **706** | QDAYNAAGGHNAVFNFPPNG | IFNg release  Proliferation  Il-2 release | qualitative binding | 1 MHC ligand assays |
| **499** | **710** | DIKVQFQSGGNNSPAVYLLD | IFNg release  Proliferation | qualitative binding | 1 MHC ligand assays |
| **500** | **711** | NNSPAVYLLDGLRAQDDYNG | IFNg release  Proliferation | qualitative binding | 1 MHC ligand assays |
| **501** | **712** | SWEYWGAQLNAMKGDLQSSL | IFNg release |  |  |
| **502** | **713** | DDYNGWDINTPAFEWYYQ | IFNg release  Proliferation  Il-2 release |  |  |
| **503** | **714** | WYSPACGKAGCQTYKWET | IFNg release  Proliferation  IL-2 release |  |  |
| **504** | **716** | CGNGTPNELGGANIPAEFLE | proliferation | qualitative binding | 1 MHC ligand assay |
| **505** | **717** | SSDPAWERNDPTQQIPKLVA | IFNg release |  |  |
| **506** | **718** | EYWGAQLNAMKGDLQSSLGA | IFNg release |  |  |
| **507** | **719** | LTSELPQWLSANRAVKPTGS | IFNg release  Proliferation | qualitative binding | 1 MHC ligand assays |
| **508** | **720** | PSLIGLAMGDAGGYKAADMW | IFNg release  Cytotoxicity | qualitative binding | 1 MHC ligand assays |
| **509** | **721** | NGTPNELGGANIPAEFLENF | IFNg release |  |  |
| **510** | **722** | LIGLAMGDAGGYKAADMWGP | IFNg release |  |  |
| **511** | **723** | SAMILAAYHPQQFIYAGSLS | IFNg release  Proliferation | qualitative binding | 1 MHC ligand assays |
| **512** | **724** | RNDPTQQIPKLVANNTRL | IFNg release  Proliferation  IL-2 release | qualitative binding | 1 MHC ligand assays |
| **513** | **725** | HSWEYWGAQLNAMKGDLQ | IFNg release  Proliferation  IL-2 release | qualitative binding | 1 MHC ligand assays |
| **514** | **726** | YWGAQLNAMKGDLQSSLGAG | Proliferation |  | 1 MHC ligand assay |
| **515** | **727** | AAIGLSMAGSSAMILAAYHPQ | Proliferation | qualitative binding |  |
| **516** | **728** | QQFIYAGSLSALLDPSQGMG | IFNg release  Proliferation | qualitative binding | 1 MHC ligand assays |
| **517** | **729** | WVYCGNGTPNELGGANIP | IFNg release  Proliferation  IL-2 release |  |  |
| **518** | **731** | YYQSGLSIVMPVGGQSSFYS | IFNg release  Proliferation | qualitative binding | 1 MHC ligand assays |
| **519** | **733** | KLVANNTRLWVYCGNGTP | IFNg release  Proliferation  IL-2 release |  |  |
| **520** | **736** | WGRRLMIGTAAAVVLPGLVG | IFNg release  Proliferation | qualitative binding | 1 MHC ligand assays |
| **521** | **737** | ALLDPSQGMGPSLIGLAMGD | Proliferation | qualitative binding | 1 MHC ligand assays |
| **522** | **738** | TPAFEWYYQSGLSIVMPV | IFNg release  Proliferation  IL-2 release |  |  |
| **523** | **741** | NELGGANIPAEFLENFVR | IFNg release  Proliferation  IL-2 release |  |  |
| **524** | **742** | GGQSSFYSDWYSPACGKA | IFNg release  Proliferation  IL-2 release |  |  |
| **525** | **743** | GGYKAADMWGPSSDPAWE | IFNg release  Proliferation  IL-2 release |  |  |
| **526** | **746** | QFIYAGSLSALLDPSQGMG | Proliferation |  | 1 MHC ligand assays |
| **527** | **747** | ANRAVKPTGSAAIGLSMAGS | IFNg release  Proliferation | qualitative binding | 1 MHC ligand assays |
| **528** | **749** | GRDIKVQFQSGGNNSPAV | IFNg release  Proliferation  IL-2 release |  |  |
| **529** | **752** | SMAGSSAMILAAYHPQQF |  | Qualitative binding |  |
| **530** | **753** | ILAAYHPQQFIYAGSLSA | IFNg release  Proliferation |  |  |
| **531** | **754** | FLTSELPQWLSANRAVKP | IFNg release  Proliferation  IL-2 release |  |  |
| **532** | **755** | SGGNNSPAVYLLDGLRAQ | IFNg release  Proliferation  IL-2 release |  |  |
| **533** | **757** | AAYHPQQFIYAGSLSALL | IFNg release  Proliferation  IL-2 release |  |  |
| **534** | **758** | FIYAGSLSALLDPSQGMG | IFNg release  Proliferation |  |  |
| **535** | **759** | SGLSIVMPVGGQSSFYSD | IFNg release  Proliferation  IL-2 release |  |  |
| **536** | **761** | FPPNGTHSWEYWGAQ | IFNg release  Proliferation |  |  |
| **537** | **763** | NFPPNGTHSWEYWGA |  |  | 3 MHC ligand assay |
| **538** | **766** | PAFEWYYQSGLSIVMP | IL-2 release |  |  |
| **539** | **767** | YNGWDINTPAFEWYY |  | Qualitative binding | 3 MHC ligand assay |
| **540** | **768** | YAGSLSALLDPSQGMGPS | IFNg release  Proliferation  IL-2 release |  |  |
| **541** | **769** | AVFNFPPNGTHSWEY |  |  | 3 MHC ligand assay |
| **542** | **773** | NTRLWVYCGNGTPNE |  |  | 3 MHC ligand assay |
| **543** | **774** | PNGTHSWEYWGAQLN | IFNg release  Proliferation  IL-2 release |  | 3 MHC ligand assay |
| **544** | **782** | LSANRAVKPTGSAAIGLS | IFNg release  Proliferation  IL-2 release |  |  |
| **545** | **783** | WGPSSDPAWERNDPT |  | qualitative binding | 3 MHC ligand assay |
| **546** | **784** | ADMWGPSSDPAWERN | IFNg release | Qualitative binding | 3 MHC ligand assay |
| **547** | **785** | QSSFYSDWYSPACGK |  | qualitative binding | 3 MHC ligand assay |
| **548** | **786** | PAWERNDPTQQIPKL | IFNg release  Proliferation |  | 3 MHC ligand assay |
| **549** | **789** | QIPKLVANNTRLWVY | IFNg release  Proliferation |  |  |
| **550** | **791** | NTPAFEWYYQSGLSI |  |  | 3 MHC ligand assay |
| **551** | **792** | KFQDAYNAAGGHNAVF |  | qualitative binding |  |
| **552** | **793** | PAEFLENFVRSSNLK | IFNg release | Qualitative binding | 3 MHC ligand assay |
| **553** | **794** | AFEWYYQSGLSIVMP |  |  | 3 MHC ligand assay |
| **554** | **798** | SSFYSDWYSPACGKA | IFNg release  Proliferation |  |  |
| **555** | **800** | AADMWGPSSDPAWER | IFNg release  Proliferation |  |  |
| **556** | **801** | AMILAAYHPQQFIYA |  |  | 3 MHC ligand assay |
| **557** | **804** | WGAQLNAMKGDLQSSL |  | qualitative binding |  |
| **558** | **805** | RKIRAWGRRLMIGTA | IFNg release  Proliferation |  |  |
| **559** | **809** | KWETFLTSELPQWLS | IFNg release |  | 3 MHC ligand assay |
| **560** | **814** | LPQWLSANRAVKPTG | IFNg release |  | 3 MHC ligand assay |
| **561** | **816** | AAGGHNAVFNFPPNG | IFNg release  proliferation |  | 3 MHC ligand assay |
| **562** | **819** | YHPQQFIYAGSLSAL |  |  | 3 MHC ligand assay |
| **563** | **821** | TGSAAIGLSMAGSSAMIL | IFNg release  Proliferation  IL-2 release |  |  |
| **564** | **822** | LAAYHPQQFIYAGSL |  |  | 3 MHC ligand assay |
| **565** | **823** | PQWLSANRAVKPTGS | IFNg release  proliferation |  |  |
| **566** | **824** | VGGQSSFYSDWYSPA |  |  | 3 MHC ligand assay |
| **567** | **826** | GSSAMILAAYHPQQF |  | Qualitative binding | 3 MHC ligand assay |
| **568** | **827** | FQSGGNNSPAVYLLD | IFNg release  proliferation |  |  |
| **569** | **831** | TSELPQWLSANRAVK |  | qualitative binding | 3 MHC ligand assay |
| **570** | **835** | WETFLTSELPQWLSA | IFNg release |  |  |
| **571** | **836** | HPQQFIYAGSLSALL | IFNg release |  |  |
| **572** | **837** | LAMGDAGGYKAADMW | IFNg release  proliferation |  |  |
| **573** | **839** | SMGRDIKVQFQSGGN | IFNg release |  |  |
| **574** | **841** | QSGGNNSPAVYLLDG |  | qualitative binding | 3 MHC ligand assay |
| **575** | **850** | GTHSWEYWGAQLN | - |  |  |
| **576** | **852** | LSIVMPVGGQSSFYS | IFNg release  proliferation | Qualitative binding | 1 MHC ligand assay |
| **577** | **853** | WLSANRAVKPTGSAA |  | qualitative binding | 1 MHC ligand assay |
| **578** | **854** | SQGMGPSLIGLAMGD | IFNg release  proliferation |  | 3 MHC ligand assay |
| **579** | **858** | PAVSQFNARTADGINYRVLWQAAGP | IFNg release |  |  |
| **580** | **859** | YRVLWQAAGPDTISGATIPQGEQST | IFNg release |  |  |
| **581** | **860** | YPITGKLGSELTMTDTVGQVVLGWK | IFNg release |  |  |
| **582** | **861** | ATIPQGEQSTGKIYFDVTGPSPTIV | IFNg release |  |  |
| **583** | **862** | DVTGPSPTIVAMNNGMEDLLIWEP | IFNg release |  |  |
| **584** | **863** | TVGQVVLGWKVSDLKSSTAVIPGYP | IFNg release |  |  |
| **585** | **864** | WEATATVNAIRGSVTPAVSQFNART | IFNg release |  |  |
| **586** | **865** | SSTAVIPGYPVAGQVWEATATVNAI | IFNg release |  |  |
| **587** | **866** | MAAIATFAAPVALAAYPITGKLGSE | IFNg release |  |  |
| **588** | **867** | IRGSVTPAVSQFNARTADGI | IFNg release |  |  |
| **589** | **868** | VSDLKSSTAVIPGYPVAGQV | IFNg release |  |  |
| **590** | **869** | GINYRVLWQAAGPDT |  | qualitative binding | 3 MHC ligand assay |
| **591** | **870** | NARTADGINYRVLWQ |  |  | 3 MHC ligand assay |
| **592** | **871** | SQFNARTADGINYRV |  |  | 3 MHC ligand assay |
| **593** | **873** | VAMNNGMEDLLIWEP |  |  | 3 MHC ligand assay |
| **594** | **874** | TADGINYRVLWQAAG |  |  | 3 MHC ligand assay |
| **595** | **875** | EQSTGKIYFDVTGPS |  |  | 3 MHC ligand assay |
| **596** | **877** | TGKIYFDVTGPSPTI |  |  | 3 MHC ligand assay |
| **597** | **878** | PGYPVAGQVWEATAT |  | qualitative binding | 3 MHC ligand assay |
| **598** | **879** | AVIPGYPVAGQVWEA |  |  | 3 MHC ligand assay |
| **599** | **880** | IYFDVTGPSPTIVAM | IFNg release |  | 3 MHC ligand assay |
| **600** | **882** | MTDTVGQVVLGWKVS |  |  | 3 MHC ligand assay |
| **601** | **883** | DLKSSTAVIPGYPVA |  |  | 3 MHC ligand assay |
| **602** | **884** | VNAIRGSVTPAVSQF |  |  | 3 MHC ligand assay |
| **603** | **885** | KVSDLKSSTAVIPGY |  |  | 3 MHC ligand assay |
| **604** | **886** | PVALAAYPITGKLGS |  |  | 3 MHC ligand assay |
| **605** | **887** | VALAAYPITGKLGSE | IFNg release |  |  |
| **606** | **888** | PVAGQVWEATATVNA |  | qualitative binding | 3 MHC ligand assay |
| **607** | **890** | LGWKVSDLKSSTAVI |  |  | 3 MHC ligand assay |
| **608** | **891** | FAAPVALAAYPITGK |  |  | 3 MHC ligand assay |
| **609** | **892** | TFAAPVALAAYPITG | IFNg release |  |  |
| **610** | **894** | IATFAAPVALAAYPI |  |  | 3 MHC ligand assay |
| **611** | **895** | QVVLGWKVSDLKSST |  | qualitative binding | 3 MHC ligand assay |
| **612** | **897** | AMNNGMEDL | IFNg release |  |  |
| **613** | **898** | AIRGSVTPAV | IFNg release  Cytotoxicity |  | 1 MHC ligand assay |
| **614** | **899** | VLGWKVSDL | IFNg release  Cytotoxicity |  | 1 MHC ligand assay |
| **615** | **900** | AAYPITGKL | IFNg release  Cytotoxicity |  | 1 MHC ligand assay |
| **616** | **901** | QVWEATATV | IFNg release  Cytotoxicity |  | 1 MHC ligand assay |
| **617** | **902** | TMTDTVGQV | IFNg release  Cytotoxicity |  | 1 MHC ligand assay |
| **618** | **903** | TMIKTAVAVV | IFNg release  Cytotoxicity |  | 1 MHC ligand assay |
| **619** | **904** | MKLTTMIKT | - |  |  |
| **620** | **905** | ATFAAPVAL | IFNg release  Cytotoxicity |  | 1 MHC ligand assay |
| **621** | **906** | AIATFAAPV | IFNg release  Cytotoxicity |  | 1 MHC ligand assay |
| **622** | **907** | KSLENYIAQTRDKFLSAATSSTPREAPYEL | Proliferation |  |  |
| **623** | **908** | NYQNFAVTNDGVIFFFNPGELLPEA | type IV hypersensitivity (DTH) |  |  |
| **624** | **909** | AVTNDGVIFFFNPGELLPEAAGPTQ | type IV hypersensitivity (DTH) |  |  |
| **625** | **910** | GVIFFFNPGELLPEAAGPTQVLVPR | type IV hypersensitivity (DTH) |  |  |
| **626** | **911** | HPTTTYKAFDWDQAYRKPIT | IFNg release  Proliferation | Qualitative binding |  |
| **627** | **912** | WDQAYRKPITYDTLWQADTD | IFNg release  Proliferation | Qualitative binding |  |
| **628** | **913** | APKTYCEELKGTDTGQACQI | IFNg release  Proliferation | Qualitative binding |  |
| **629** | **914** | NDGVIFFFNPGELLPEAAGP | IFNg release  Proliferation  cytotoxicity | Qualitative binding |  |
| **630** | **915** | GTDTGQACQIQMSDPAYNIN | IFNg release  Proliferation  cytotoxicity | Qualitative binding |  |
| **631** | **916** | LKVYQNAGGTHPTTTYKAFD | IFNg release  Proliferation | Qualitative binding |  |
| **632** | **917** | CQIQMSDPAYNINISLPSYY | IFNg release  Proliferation |  |  |
| **633** | **918** | MRIKIFMLVTAVVLLCCSGV | IFNg release  Proliferation |  |  |
| **634** | **919** | GLDPVNYQNFAVTNDGVIFF | IFNg release  Proliferation |  |  |
| **635** | **920** | YDTLWQADTDPLPVVFPIVQ | IFNg release  Proliferation | Qualitative binding |  |
| **636** | **921** | PVNYQNFAVTNDGVIFFFNP | IFNg release  Proliferation | Qualitative binding |  |
| **637** | **922** | QMSDPAYNINISLPSYYPDQ | IFNg release  Proliferation  Cytotoxicity | Qualitative binding |  |
| **638** | **923** | PDQKSLENYIAQTRDKFLSA | IFNg release  Proliferation |  |  |
| **639** | **924** | IQMSDPAYNINISLPSYYPD | antibody help | Qualitative binding |  |
| **640** | **925** | IKIFMLVTAVVLLCCSGVAT | IFNg release |  |  |
| **641** | **926** | IPPRGTQAVVLKVYQNAGGT | IFNg release  Proliferation  Cytotoxicity | Qualitative binding |  |
| **642** | **927** | ISLPSYYPDQKSLENYIAQT | IFNg release  Proliferation  Cytotoxicity | Qualitative binding |  |
| **643** | **928** | DTDPLPVVFPIVQGELSKQT | IFNg release  Proliferation |  |  |
| **644** | **929** | AIPPRGTQAVVLKVYQNAGG | IFNg release |  |  |
| **645** | **930** | AVVLLCCSGVATAAPKTYCE | IFNg release  Proliferation |  |  |
| **646** | **931** | VSIAPNAGLDPVNYQNFAVT | IFNg release  Proliferation | Qualitative binding |  |
| **647** | **932** | GELSKQTGQQVSIAPNAGLD | IFNg release  Proliferation  Cytotoxicity | Qualitative binding |  |
| **648** | **933** | PLPVVFPIVQGELSKQTGQQ | IFNg release  Proliferation  Cytotoxicity | Qualitative binding |  |
| **649** | **934** | YNINISLPSYYPDQKSLENY | IFNg release |  |  |
| **650** | **935** | PKTYCEELKGTDTGQACQ |  | qualitative binding |  |
| **651** | **936** | STPREAPYELNITSATYQSA | IFNg release  Proliferation | Qualitative binding |  |
| **652** | **937** | GELLPEAAGPTQVLVPRSAI | IFNg release  Proliferation  Cytotoxicity | Qualitative binding |  |
| **653** | **938** | NITSATYQSAIPPRGTQAVV | IFNg release  Proliferation | Qualitative binding |  |
| **654** | **940** | RDKFLSAATSSTPREAPYEL | IFNg release  Proliferation  Cytotoxicity | Qualitative binding |  |
| **655** | **941** | TNDGVIFFFNPGELL | qualitative binding |  |  |
| **656** | **943** | QNAGGTHPTTTYKAF | qualitative binding |  |  |
| **657** | **944** | SAATSSTPREAPYELN | qualitative binding |  |  |
| **658** | **945** | ENYIAQTRDKFLSAA | qualitative binding |  |  |
| **659** | **946** | TAVVLLCCSGVATAA | qualitative binding |  |  |
| **660** | **947** | TQVLVPRSAIDSMLA | IFNg release  Proliferation | Qualitative binding |  |
| **661** | **948** | LLCCSGVATA | cytotoxicity |  | 1 MHC ligand assay |
| **662** | **949** | TTTYKAFDW |  |  | 1 MHC ligand assay |
| **663** | **950** | VTNDGVIFF |  |  | 4 MHC ligand assay |
| **664** | **951** | KIFMLVTAVV | cytotoxicity |  | 1 MHC ligand assay |
| **665** | **953** | TPREAPYEL |  |  | 2 MHC ligand assay |
| **666** | **954** | FAVTNDGVI | IFNg releas  pathogen burden after challenge  TNFa release  Cytotoxicity  IL-4 release |  |  |
| **667** | **955** | REAPYELNI |  |  | 6 MHC ligand assay |
| **668** | **956** | SYYPDQKSL | Cytotoxicity |  | 1 MHC ligand assay |
| **669** | **957** | KTYCEELK |  |  | 1 MHC ligand assay |
| **670** | **959** | ELNITSATY |  |  | 4 MHC ligand assay |
| **671** | **960** | GTHPTTTYK | IFNg release  proliferation |  | 4 MHC ligand assay |
| **672** | **961** | GTQAVVLK |  |  | 1 MHC ligand assay |
| **673** | **962** | LRPTFDTRLMRLEDEMKEGRYE | proliferation |  |  |
| **674** | **963** | RDGQLTIKAERTEQKDFDGRS | proliferation |  |  |
| **675** | **964** | MATTLPVQRHPRSLFPEFSE | IFNg release  Proliferation  IL-4 release | Qualitative binding | 3 MHC ligand assay |
| **676** | **965** | RTEQKDFDGRSEFAYGSFVR | IFNg release  Proliferation  IL-4 release | Qualitative binding | 3 MHC ligand assay |
| **677** | **966** | LFAAFPSFAGLRPTFDTRLM | IFNg release  Proliferation  IL-4 release | Qualitative binding | 19 MHC ligand assay |
| **678** | **968** | YEVRAELPGVDPDKDVDIMV | IFNg release  Proliferation  IL-4 release | Qualitative binding | 3 MHC ligand assay |
| **679** | **969** | SEFAYGSFVRTVSLPVGADE | IFNg release  Proliferation  IL-4 release  pathogen burden after challenge  IL-17 release  TNFa release  Cytotoxicity  IL-4 release  decreased disease  IL-2 release  IL-12 release  IL-17a release  perforin release  IL-10 release |  |  |
| **680** | **971** | RLEDEMKEGRYEVRAELPGV | IFNg release  Proliferation  IL-4 release | Qualitative binding | 3 MHC ligand assay |
| **681** | **972** | TVSLPVGADEDDIKATYDKG | IFNg release  Proliferation  IL-4 release | Qualitative binding | 3 MHC ligand assay |
| **682** | **973** | DPDKDVDIMVRDGQLTIKAE | IFNg release  Proliferation  IL-4 release | Qualitative binding | 3 MHC ligand assay |
| **683** | **974** | PRSLFPEFSELFAAFPSFAG | IFNg release  Proliferation  IL-4 release | Qualitative binding | 3 MHC ligand assay |
| **684** | **975** | RSLFPEFSELFAAFPSFAGL | IFNg release |  |  |
| **685** | **976** | EFAYGSFVRTVSLPVGADE | IL-2 release |  | 5 MHC ligand assays |
| **686** | **977** | ILTVSVAVSEGKPTEKHIQI | IFNg release  Proliferation  IL-4 release | Qualitative binding | 3 MHC ligand assay |
| **687** | **978** | DDIKATYDKGILTVSVAVSE | IFNg release  Proliferation  IL-4 release | Qualitative binding | 12 MHC ligand assay |
| **688** | **979** | SVAVSEGKPTEKHIQIRSTN | Proliferation |  | 3 MHC ligand assays |
| **689** | **980** | FAYGSFVRTVSLPVGADE |  |  | 5 MHC ligand assays |
| **690** | **982** | PVQRHPRSLFPEFSE | IFNg release  Cytotoxicity |  | 26 MHC ligand assay |
| **691** | **984** | PSFAGLRPTFDTRLM | IFNg release  Cytotoxicity |  | 26 MHC ligand assay |
| **692** | **986** | FPSFAGLRPTFDTRL |  | qualitative binding |  |
| **693** | **988** | DFDGRSEFAYGSFVR | IFNg release  Cytotoxicity |  | 26 MHC ligand assay |
| **694** | **989** | KDFDGRSEFAYGSFV | IFNg release |  |  |
| **695** | **990** | FDGRSEFAYGSFVRT | IFNg release |  |  |
| **696** | **991** | MKEGRYEVRAELPGV | IFNg release  Cytotoxicity |  | 26 MHC ligand assay |
| **697** | **992** | DGRSEFAYGSFVRTV | IFNg release |  |  |
| **698** | **993** | AYGSFVRTVSLPVGA | IFNg release |  | 34 MHC ligand assay |
| **699** | **994** | YGSFVRTVSLPVGAD | IFNg release |  |  |
| **700** | **998** | TIKAERTEQKDFDGR | IFNg release  Cytotoxicity |  | 26 MHC ligand assay |
| **701** | **1000** | GRSEFAYGSFVRTVS | IFNg release |  | 31 MHC ligand assay |
| **702** | **1004** | FVRTVSLPVGADEDD | IFNg release |  |  |
| **703** | **1005** | RSEFAYGSFVRTVSL | IFNg release |  |  |
| **704** | **1006** | GSFVRTVSLPVGADE | IFNg release  cytotoxicity |  | 26 MHC ligand assay |
| **705** | **1007** | ELPGVDPDKDVDIMV | IFNg release  cytotoxicity |  | 26 MHC ligand assay |
| **706** | **1008** | PEFSELFAAFPSFAG | IFNg release  cytotoxicity |  | 26 MHC ligand assay |
| **707** | **1009** | VDIMVRDGQLTIKAE | IFNg release  cytotoxicity |  | 26 MHC ligand assay |
| **708** | **1010** | EGKPTEKHIQIRSTN | IFNg release |  | 26 MHC ligand assay |
| **709** | **1012** | DTRLMRLEDEMKEGR | IFNg release  cytotoxicity |  | 26 MHC ligand assay |
| **710** | **1014** | EDEMKEGRYEVRAEL |  | qualitative binding |  |
| **711** | **1015** | VAVSEGKPTEKHIQI | IFNg release  cytotoxicity |  | 26 MHC ligand assay |
| **712** | **1017** | GKPTEKHIQIRSTN | IFNg release  Cytotoxicity  Proliferation  IL-4 release | Qualitative binding |  |
| **713** | **1019** | VSVAVSEGKPTEKHI |  | qualitative binding |  |
| **714** | **1020** | TYDKGILTVSVAVSE | IFNg release  cytotoxicity |  | 26 MHC ligand assay |
| **715** | **1022** | VGADEDDIKATYDKG | IFNg release  cytotoxicity |  | 33 MHC ligand assay |
| **716** | **1031** | ATYDKGILTV | cytotoxicity |  | 1 MHC ligand assay |
| **717** | **1033** | RPTFDTRLM |  |  | 1 MHC ligand assay |
| **718** | **1035** | TLPVQRHPR |  | Qualitative binding |  |
