# Supplementary material for: Design of a multi-epitope vaccine against Mycobacterium tuberculosis using reverse vaccinology and immunoreactive peptides
Source: Genomics Inform. 2026 Jul 8;24:13. doi: 10.1186/s44342-026-00075-6 (PMC13348615; doi:10.1186/s44342-026-00075-6)
Supplement: Supplementary file 4 — Supplementary Material 4: Data 4. Identification of conserved, antigenic and non-allergen Epitopes IFN-γ releasing epitopes. Out of 460 IFN-γ releasing epitopes, 152 showed a conservancy score > 95%. Subsequently 14 antigenic and non-allergenic epitopes were identified. [file 44342_2026_75_MOESM4_ESM.docx]

# Supplementary Data 4. Identification of conserved, antigenic and non-allergen Epitopes IFN-γ releasing epitopes. Out of 460 IFN-γ releasing epitopes, 152 showed a conservancy score > 95%. Subsequently 14 antigenic and non-allergenic epitopes were identified.

- **Four hundred and sixty IFN-γ releasing epitopes**

| Header | Peptide | Relevant protein | Conservancy |
| --- | --- | --- | --- |
| 11 | KTQGPGAWPK | rpfD | 94.12% |
| 12 | NARTTLIAAAIAGTL | rpfE | 90.40% |
| 13 | RTTLIAAAIAGTLVT | rpfE | 90.40% |
| 31 | VLMGGVPGVE | ald | 96.83% |
| 39 | YASVEAANASPLQVA | RS03355 | 99.30% |
| 40 | FVQALTTAAASYASV | RS03355 | 100% |
| 44 | GDLVGPGCAEYAAANPTGPASVQGM | RS03490 | 85.71% |
| 46 | ALSGQLNPQVNLVDTLNSGQYTVFA | RS03490 | 89.29% |
| 47 | GNADVVCGGVSTANATVYMIDSVLM | RS03490 | 89.29% |
| 48 | PTGPASVQGMSQDPVAVAASNNPEL | RS03490 | 96.43% |
| 49 | GASVTVTGQGNSLKVGNADVVCGGV | RS03490 | 85.71% |
| 50 | SSLLTSILTYHVVAGQTSPANVVGT | RS03490 | 85.71% |
| 51 | QTSPANVVGTRQTLQGASVTVTGQG | RS03490 | 96.43% |
| 52 | AVAASNNPELTTLTAALSGQLNPQV | RS03490 | 92.86% |
| 54 | LNPQVNLVDTLNSGQYTVFA | RS03490 | 89.29% |
| 56 | FSKLPASTIDELKTNSSLLTSILTY | RS03490 | 92.86% |
| 57 | YAAANPTGPASVQGMSQDPV | RS03490 | 85.71% |
| 58 | VCGGVSTANATVYMIDSVLM | RS03490 | 92.86% |
| 59 | PTNAAFSKLPASTIDELKTN | RS03490 | 96.43% |
| 60 | GVSTANATVYMIDSVLMPPA | RS03490 | 92.86% |
| 61 | NSLKVGNADVVCGGVSTANA | RS03490 | 89.29% |
| 64 | IDELKTNSSLLTSILTYHVV | RS03490 | 89.29% |
| 66 | MKVKNTIAATSFAAAGLAAL | RS03490 | 82.14% |
| 69 | ANATVYMIDSVLMPP | RS03490 | 100.00% |
| 78 | GLAALAVAVSPPAAAGDLVGPGCAE | RS03490 | 60.71% |
| 79 | TVYMIDSVLMPPA | RS03490 | 100.00% |
| 80 | VNLVDTLNSGQYT | RS03490 | 96.43% |
| 81 | AAFSKLPASTIDELK | RS03490 | 96.43% |
| 84 | NAAFSKLPAST | RS03490 | 96.43% |
| 96 | VPWQPAFVF | RS03505 | 93.75% |
| 97 | LLVIPVALSASIIRL | RS04220 | 84.44% |
| 112 | LGRWKWHDPWVHASLLAQNN | fbpD | 94.64% |
| 113 | TRVWVWSPTNPGASDPAAMI | fbpD | 94.64% |
| 116 | VAFLAGGPHAVYLLD | fbpD | 94.64% |
| 117 | PVAFLAGGPHAVYLL | fbpD | 92.86% |
| 122 | GGPHAVYLL | fbpD | 94.64% |
| 124 | TLAGKGISVV | fbpD | 94.64% |
| 125 | LAGKGISVV | fbpD | 94.64% |
| 127 | KAGCQTYKWETFLTSELPGWLQANRHV | ag85A | 86.79% |
| 128 | GLSVVMPVGGQSSFYSDWYQPAC | ag85A | 94.34% |
| 129 | FSRPGLPVEYLQVPSPSMGRDIK | ag85A | 92.45% |
| 130 | LTSELPGWLQANRHVKPTGSAVV | ag85A | 88.68% |
| 131 | DWYQPACGKAGCQTYKWETF | ag85A | 98.11% |
| 132 | THSWEYWGAQLNAMKPDLQR | ag85A | 98.11% |
| 133 | NGVFDFPDSGTHSWEYWGAQ | ag85A | 98.11% |
| 134 | GCQTYKWETFLTSELPGWLQ | ag85A | 94.34% |
| 135 | MKPDLQRALGATPNTGPAPQGA | ag85A | 81.13% |
| 136 | GLRAQDDFSGWDINTPAFEW | ag85A | 86.79% |
| 137 | WDINTPAFEWYDQSGLSVVM | ag85A | 83.02% |
| 138 | IANNTRVWVYCGNGKPSDLG | ag85A | 90.57% |
| 139 | QRNDPLLNVGKLIANNTRVW | ag85A | 96.23% |
| 140 | NDPLLNVGKLIANNTRVWVY | ag85A | 92.45% |
| 141 | PVGGQSSFYSDWYQPACGKA | ag85A | 98.11% |
| 142 | GNNLPAKFLEGFVRTSNIKF | ag85A | 98.11% |
| 143 | AGGYKASDMWGPKEDPAWQR | ag85A | 88.68% |
| 144 | TFLTSELPGWLQANRHVKPT | ag85A | 88.68% |
| 145 | GPKEDPAWQRNDPLLNVGKL | ag85A | 96.23% |
| 148 | PDSGTHSWEYWGAQLNAMK | ag85A | 96.23% |
| 149 | QDAYNAGGGHNGVFDFPDSG | ag85A | 94.34% |
| 150 | KPDLQRALGATPNTGPAPQGA | ag85A | 83.02% |
| 152 | LAIYHPQQFVYAGAMSGLLD | ag85A | 98.11% |
| 155 | PTLIGLAMGDAGGYKASDMW | ag85A | 88.68% |
| 156 | LGGNNLPAKFLEGFVRTSNI | ag85A | 98.11% |
| 157 | SALTLAIYHPQQFVYAGAMS | ag85A | 98.11% |
| 158 | GFVRTSNIKFQDAYNAGGGH | ag85A | 94.34% |
| 159 | LQVPSPSMGRDIKVQFQSGG | ag85A | 92.45% |
| 160 | DIKVQFQSGGANSPALYLLD | ag85A | 96.23% |
| 161 | ANSPALYLLDGLRAQDDFSG | ag85A | 90.57% |
| 162 | DPAWQRNDPLLNVGKLIAN | ag85A | 98.11% |
| 163 | GNGKPSDLGGNNLPAKFLEG | ag85A | 96.23% |
| 164 | QQFVYAGAMSGLLDPSQAMG | ag85A | 96.23% |
| 166 | YDQSGLSVVMPVGGQSSFYS | ag85A | 86.79% |
| 168 | ALYLLDGLRAQDDFSGWDI | ag85A | 90.57% |
| 169 | GLLDPSQAMGPTLIGLAMGD | ag85A | 94.34% |
| 170 | ANRHVKPTGSAVVGLSMAAS | ag85A | 88.68% |
| 171 | YLQVPSPSMGRDIKVQFQ | ag85B | 92.45% |
| 172 | LQANRHVKPTGSAVVGLSM | ag85A | 90.57% |
| 175 | AVVGLSMAASSALTLAIYHP | ag85A | 98.11% |
| 176 | VFDFPDSGTHSWEYW | ag85A | 98.11% |
| 177 | ACGKAGCQTYKWETF | ag85A | 98.11% |
| 178 | FYSDWYQPACGKAGC | ag85A | 98.11% |
| 179 | FSGWDINTPAFEWYD | ag85A | 84.91% |
| 180 | AGCQTYKWETFLTSE | ag85A | 96.23% |
| 181 | GKLIANNTRVWVYCG | ag85A | 92.45% |
| 183 | KEDPAWQRNDPLLNV | ag85A | 98.11% |
| 184 | NNTRVWVYCGNGKPS | ag85A | 90.57% |
| 186 | DSGTHSWEYWGAQLN | ag85A | 98.11% |
| 188 | SWEYWGAQLNAMKPD | ag85A | 98.11% |
| 189 | GGQSSFYSDWYQPAC | ag85A | 98.11% |
| 190 | SELPGWLQANRHVKP | ag85A | 90.57% |
| 191 | FLEGFVRTSNIKFQD | ag85A | 98.11% |
| 192 | PGWLQANRHVKPTGS | ag85A | 90.57% |
| 193 | LTLAIYHPQQFVYAG | ag85A | 100.00% |
| 194 | KWETFLTSELPGWLQ | ag85A | 96.23% |
| 195 | RAQDDFSGWDINTPA | ag85A | 90.57% |
| 196 | QTYKWETFLTSELPG | ag85A | 96.23% |
| 198 | PAFEWYDQSGLSVVM | ag85A | 86.79% |
| 199 | VMPVGGQSSFYSDWY | ag85A | 96.23% |
| 200 | FEWYDQSGLSVVMPV | ag85A | 86.79% |
| 201 | GGHNGVFDFPDSGTH | ag85A | 96.23% |
| 202 | WLQANRHVKPTGSAV | ag85A | 90.57% |
| 203 | WQRNDPLLNVGKLIA | ag85A | 100.00% |
| 204 | AYNAGGGHNGVFDFP | ag85A | 96.23% |
| 205 | YHPQQFVYAGAMSGL | ag85A | 98.11% |
| 206 | PVEYLQVPSPSMGRD | ag85A | 92.45% |
| 207 | AIYHPQQFVYAGAMS | ag85A | 98.11% |
| 209 | VEYLQVPSPSMGRDI | ag85A | 92.45% |
| 210 | VVMPVGGQSSFYSDW | ag85A | 96.23% |
| 211 | ETFLTSELPGWLQAN | ag85A | 92.45% |
| 214 | PLLNVGKLIANNTRV | ag85A | 96.23% |
| 215 | LPVEYLQVPSPSMGR | ag85A | 92.45% |
| 218 | LLDGLRAQDDFSGWD | ag85A | 90.57% |
| 220 | PSPSMGRDIKVQFQS | ag85B | 94.34% |
| 221 | ATAGAFSRPGLPVEY | ag85A | 96.23% |
| 223 | KVQFQSGGANSPALY | ag85A | 96.23% |
| 227 | FVYAGAMSGLLDPSQ | ag85A | 96.23% |
| 228 | FQSGGANSPALYLLD | ag85A | 96.23% |
| 229 | GAQLNAMKPDLQRAL | ag85A | 96.23% |
| 230 | SPALYLLDGLRAQDD | ag85A | 92.45% |
| 233 | AMSGLLDPSQAMGPT | ag85A | 96.23% |
| 235 | MGRDIKVQFQSGGAN | ag85A | 98.11% |
| 236 | PSMGRDIKVQFQSGG | ag85A | 98.11% |
| 237 | LSVVMPVGGQSSFYS | ag85A | 96.23% |
| 239 | ALGATPNTGPAPQGA | ag85A | 83.02% |
| 240 | AMGPTLIGLAMGDAG | ag85A | 96.23% |
| 241 | SMAASSALTLAIYHP | ag85A | 98.11% |
| 242 | QSGLSVVMPVGGQSS | ag85A | 96.23% |
| 253 | LAQEAGNFERISGDLKTQID | esxB | 92.68% |
| 254 | QKQELDEISTNIRQAGVQYS | esxB | 82.93% |
| 255 | EISTNIRQAGVQYSRADEEQ | esxB | 95.12% |
| 257 | VQYSRADEEQQQALSSQMGF | esxB | 92.68% |
| 258 | LDEISTNIRQAGVQYSRAD | esxB | 95.12% |
| 260 | QVESTAGSLQGQWRGAAGTA | esxB | 82.93% |
| 261 | ISGDLKTQIDQVESTAGSLQ | esxB | 87.80% |
| 262 | AQAAVVRFQEAANKQKQELD | esxB | 46.34% |
| 263 | ELDEISTNIRQAGVQYSR | esxB | 87.80% |
| 264 | ATLAQEAGNFERISGDLK | esxB | 95.12% |
| 265 | DQVESTAGSLQGQWRGAA | esxB | 87.80% |
| 267 | VVRFQEAANKQKQELDEI | esxB | 51.22% |
| 268 | NIRQAGVQYSRADEEQQQ | esxB | 95.12% |
| 270 | NFERISGDLKTQIDQV | esxB | 90.24% |
| 271 | GDLKTQIDQVESTAGSL | esxB | 90.24% |
| 272 | QEAGNFERISGDLKTQ | esxB | 92.68% |
| 273 | IDQVESTAGSLQGQWR | esxB | 87.80% |
| 274 | AGTAAQAAVVRFQEAANK | esxB | 53.66% |
| 275 | SRADEEQQQALSSQMGF | esxB | 95.12% |
| 277 | AAVVRFQEAANKQKQEL | esxB | 51.22% |
| 282 | TNIRQAGVQYSRADE | esxB | 95.12% |
| 284 | RADEEQQQALSSQMGF | esxB | 95.12% |
| 285 | DEISTNIRQAGVQYS | esxB | 95.12% |
| 288 | STNIRQAGVQYSRAD | esxB | 95.12% |
| 291 | KQELDEISTNIRQAG | esxB | 87.80% |
| 292 | ISTNIRQAGVQYSRA | esxB | 95.12% |
| 293 | ERISGDLKTQIDQVE | esxB | 90.24% |
| 294 | QELDEISTNIRQAGV | esxB | 90.24% |
| 296 | DAATLAQEAGNFERI | esxB | 95.12% |
| 298 | TAGSLQGQWRGAAGT | esxB | 85.37% |
| 299 | TDAATLAQEAGNFER | esxB | 97.56% |
| 300 | VRFQEAANKQKQELD | esxB | 56.10% |
| 301 | STAGSLQGQWRGAAG | esxB | 90.24% |
| 302 | KTDAATLAQEAGNFE | esxB | 97.56% |
| 305 | RQAGVQYSRADEEQQ | esxB | 95.12% |
| 306 | FQEAANKQKQELDEI | esxB | 63.41% |
| 307 | GSLQGQWRGAAGTAA | esxB | 85.37% |
| 308 | AGSLQGQWRGAAGTA | esxB | 85.37% |
| 311 | SLQGQWRGAAGTAAQ | esxB | 82.93% |
| 312 | LKTQIDQVESTAGSL | esxB | 90.24% |
| 314 | LQGQWRGAAGTAAQA | esxB | 82.93% |
| 315 | KTQIDQVESTAGSLQ | esxB | 92.68% |
| 316 | ADEEQQQALSSQMGF | esxB | 95.12% |
| 317 | AGVQYSRADEEQQQA | esxB | 92.68% |
| 318 | AVVRFQEAANKQKQE | esxB | 51.22% |
| 321 | QAAVVRFQEAANKQK | esxB | 51.22% |
| 325 | AAQAAVVRFQEAANK | esxB | 56.10% |
| 326 | TAAQAAVVRFQEAAN | esxB | 53.66% |
| 328 | GTAAQAAVVRFQEAA | esxB | 53.66% |
| 336 | TQIDQVESTAGSL | esxB | 92.68% |
| 337 | EEQQQALSSQMGF | esxB | 95.12% |
| 339 | AEMKTDAATLA | esxB | 92.68% |
| 340 | QWRGAAGTAA | esxB | 92.68% |
| 342 | GQWRGAAGTAAQAAVVRFQE | esxB | 53.66% |
| 343 | QYSRADEEQQ | esxB | 97.56% |
| 344 | QQALSSQMGF | esxB | 95.12% |
| 354 | EMKTDAATL | esxB | 95.12% |
| 357 | VESTAGSL | esxB | 97.56% |
| 359 | SGSEAYQGVQQKWDATATELNNALQ | esxA | 68.75% |
| 360 | QQWNFAGIEAAASAIQGNVTSIHSL | esxA | 81.25% |
| 361 | SAIQGNVTSIHSLLDEGKQSLTKLA | esxA | 100.00% |
| 362 | MTEQQWNFAGIEAAASAIQGNVTSI | esxA | 81.25% |
| 363 | AWGGSGSEAYQGVQQKWDATATEL | esxA | 75.00% |
| 364 | EGKQSLTKLAAAWGGSGSEAYQGVQ | esxA | 81.25% |
| 365 | VTSIHSLLDEGKQSLTKLAAAWGG | esxA | 100.00% |
| 366 | QGVQQKWDATATELNNALQNLART | esxA | 68.75% |
| 367 | TATELNNALQNLARTISEAGQAMAS | esxA | 50.00% |
| 368 | LARTISEAGQAMASTEGNVTGMFA | esxA | 62.50% |
| 369 | EAAASAIQGNVTSIHSLLDEGKQS | esxA | 87.50% |
| 370 | GKQSLTKLAAAWGGSGSEAYQGVQ | esxA | 81.25% |
| 371 | WNFAGIEAAASAIQGNVTSIHSL | esxA | 81.25% |
| 373 | ATELNNALQNLARTISEAGQAMAS | esxA | 50.00% |
| 374 | LQNLARTISEAGQAMASTEGNVT | esxA | 62.50% |
| 376 | YQGVQQKWDATATELNNALQ | esxA | 75.00% |
| 378 | GIEAAASAIQGNVTSIHSLLD | esxA | 87.50% |
| 379 | NVTSIHSLLDEGKQSLTKLA | esxA | 100.00% |
| 381 | ISEAGQAMASTEGNVTGMFA | esxA | 68.75% |
| 382 | NLARTISEAGQAMASTEGNV | esxA | 62.50% |
| 383 | IEAAASAIQGNVTSIHSLLD | esxA | 87.50% |
| 386 | LNNALQNLARTISEAGQAM | esxA | 62.50% |
| 390 | SIHSLLDEGKQSLTKLAA | esxA | 100.00% |
| 391 | AIQGNVTSIHSLLDEGK | esxA | 100.00% |
| 392 | EAYQGVQQKWDATATEL | esxA | 81.25% |
| 393 | NALQNLARTISEAGQAMA | esxA | 62.50% |
| 397 | VQQKWDATATELNNAL | esxA | 81.25% |
| 399 | KWDATATELNNALQNL | esxA | 81.25% |
| 400 | IHSLLDEGKQSLTKLA | esxA | 100.00% |
| 401 | ATATELNNALQNLARTI | esxA | 68.75% |
| 402 | LDEGKQSLTKLAAAWG | esxA | 100.00% |
| 404 | QGNVTSIHSLLDEGK | esxA | 100.00% |
| 405 | GNVTSIHSLLDEGKQ | esxA | 100.00% |
| 406 | GSGSEAYQGVQQKWD | esxA | 81.25% |
| 407 | GVQQKWDATATELNN | esxA | 81.25% |
| 409 | IQGNVTSIHSLLDEG | esxA | 100.00% |
| 415 | WGGSGSEAYQGVQQK | esxA | 81.25% |
| 417 | QQKWDATATELNNAL | esxA | 87.50% |
| 418 | QKWDATATELNNALQ | esxA | 87.50% |
| 419 | TISEAGQAMASTEGNV | esxA | 68.75% |
| 420 | TSIHSLLDEGKQSLT | esxA | 100.00% |
| 421 | KQSLTKLAAAWGGSGS | esxA | 93.75% |
| 423 | HSLLDEGKQSLTKLA | esxA | 100.00% |
| 424 | ARTISEAGQAMASTEG | esxA | 62.50% |
| 425 | TEQQWNFAGIEAAAS | esxA | 81.25% |
| 426 | QAMASTEGNVTGMFA | esxA | 75.00% |
| 427 | AAASAIQGNVTSIHSL | esxA | 87.50% |
| 431 | TKLAAAWGGSGSEAY | esxA | 87.50% |
| 434 | ELNNALQNLARTISE | esxA | 68.75% |
| 436 | QWNFAGIEAAASAIQ | esxA | 81.25% |
| 438 | NNALQNLARTISEAG | esxA | 68.75% |
| 440 | EQQWNFAGIEAAASA | esxA | 81.25% |
| 441 | TELNNALQNLARTIS | esxA | 68.75% |
| 443 | QNLARTISEAGQAMA | esxA | 62.50% |
| 444 | ASAIQGNVTSIHSLL | esxA | 93.75% |
| 446 | AGQAMASTEGNVTGM | esxA | 75.00% |
| 448 | RTISEAGQAMASTEG | esxA | 62.50% |
| 452 | EAGQAMASTEGNVTG | esxA | 68.75% |
| 455 | AASAIQGNVTSIHSL | esxA | 93.75% |
| 456 | AGIEAAASAIQGNVTS | esxA | 87.50% |
| 458 | ALQNLARTISEAGQA | esxA | 68.75% |
| 459 | SEAGQAMASTEGNVT | esxA | 68.75% |
| 460 | NFAGIEAAASAIQGN | esxA | 81.25% |
| 461 | LTKLAAAWGGSGSEA | esxA | 87.50% |
| 465 | FAGIEAAASAIQGNV | esxA | 87.50% |
| 476 | SEAYQGVQQKW | esxA | 87.50% |
| 477 | STEGNVTGMFA | esxA | 87.50% |
| 488 | AYQGVQQKW | esxA | 87.50% |
| 510 | AMASTEGNV | esxA | 75.00% |
| 526 | LLDEGKQSL | esxA | 100.00% |
| 533 | MHRIFLITVALALLTASPASAIT | mycP1 | 58.28% |
| 534 | GSIRSLARAVVHAANLGVGV | mycP1 | 99.34% |
| 535 | AQIIHRITATARHPG | mycP1 | 98.01% |
| 536 | APYNVRRLPPPVVEP | mycP1 | 96.03% |
| 537 | DDLVGAGVIDAVA | mycP1 | 97.35% |
| 540 | NGVFNFPPNGTHSWPYWNEQ | ag85C | 84.91% |
| 541 | THSWPYWNEQLVAMKADIQH | ag85C | 84.91% |
| 542 | QNYTYKWETFLTREMPAWLQ | ag85C | 96.23% |
| 543 | DWYQPSQSNGQNYTYKWETF | ag85C | 94.34% |
| 544 | WDINTPAFEEYYQSGLSVIM | ag85C | 83.02% |
| 545 | GFLNPSEGWWPTLIGLAMND | ag85C | 96.23% |
| 546 | NDPMVQIPRLVANNTRIWVY | ag85C | 90.57% |
| 547 | QQFPYAASLSGFLNPSEGWW | ag85C | 94.34% |
| 548 | RDTYAADGGRNGVFNFPPNG | ag85C | 98.11% |
| 549 | SGGYNANSMWGPSSDPAWKR | ag85C | 98.11% |
| 550 | LTREMPAWLQANKGVSPTGN | ag85C | 75.47% |
| 551 | PVGGQSSFYTDWYQPSQSNG | ag85C | 96.23% |
| 553 | FSRPGLPVEYLQVPSASMGR | ag85C | 98.11% |
| 554 | YLLDGLRAQDDYNGWDIN | ag85C | 86.79% |
| 555 | YYQSGLSVIMPVGGQSSFYT | ag85C | 98.11% |
| 556 | DIKVQFQGGGPHAVYLLD | ag85C | 90.57% |
| 557 | ANKGVSPTGNAAVGLSMSGG | ag85C | 77.36% |
| 560 | GQNYTYKWETFLTRE | ag85C | 98.11% |
| 562 | VYLLDGLRAQDDYNG | ag85C | 98.11% |
| 563 | EGWWPTLIGLAMNDS | ag85C | 94.34% |
| 564 | DPMVQIPRLVANNTR | ag85C | 92.45% |
| 567 | QVPSASMGRDIKVQF | ag85C | 96.23% |
| 583 | IPVVSVTKSVGFQLRGQSGPTTVK | lpqL | 94.38% |
| 584 | AGIERTFVAYLKMAGKTAQDT | lpqL | 94.38% |
| 586 | MVNKSRMMPAV | lpqL | 96.63% |
| 598 | VVHPAVVQANRVRTWLLAVSNVFGQ | RS13395 | 95.65% |
| 599 | GQSVTGYNNSVSVTS | RS13395 | 96.27% |
| 600 | GIVALIALGILEHFD | ggtA | 96.92% |
| 601 | YTKKLWQAIRAQDVC | rpfA | 83.07% |
| 602 | TKKLWQAIRAQDVCG | rpfA | 83.07% |
| 603 | AYTKKLWQAIRAQDV | rpfA | 83.07% |
| 604 | TTSNVSVAK | rpfA | 99.61% |
| 610 | GGLVRTVHLPAPNVA | rpfB | 96.03% |
| 611 | AGVQVHDADTIVLRR | rpfB | 96.83% |
| 612 | TVHLPAPNVAGLLSA | rpfB | 97.62% |
| 613 | LPVANVVVTPAHEAV | rpfB | 89.68% |
| 614 | TAMRVTTMK | rpfB | 95.24% |
| 620 | MSFVTTQPEALAAAAGSLQGI | RS18950 | 39.13% |
| 621 | AAIHEMFVNTLQMSS | RS18950 | 43.48% |
| 622 | TGVVPAAADEVSALT | RS18950 | 34.78% |
| 625 | AQIYQAVSAQAAAIH | RS18950 | 41.30% |
| 633 | PGTRINQETVSLDANGVSGS | apa | 97.93% |
| 645 | AAMASASLVTVAVPATANAD | apa | 97.24% |
| 661 | RTVSLPVGA | hspX | 100.00% |
| 663 | AFPSFAGL | hspX | 100.00% |
| 664 | SLFPEFSEL | hspX | 100.00% |
| 665 | GILTVSVAV | hspX | 91.30% |
| 668 | ARVIMRSAIG | ripC | 98.10% |
| 686 | VFNFPPNGTHSWEYWGAQLN | ag85B | 61.59% |
| 689 | PTQQIPKLVANNTRLWVYCG | ag85B | 54.27% |
| 690 | FQDAYNAAGGHNAVFNFPPNG | ag85B | 60.37% |
| 692 | WDINTPAFEWYYQSGLSIVM | ag85B | 56.71% |
| 693 | NIPAEFLENFVRSSNLKFQD | ag85B | 60.37% |
| 694 | AEFLENFVRSSNLKFQDAYN | ag85B | 59.76% |
| 695 | THSWEYWGAQLNAMKGDLQS | ag85B | 57.93% |
| 697 | GYKAADMWGPSSDPAWERND | ag85B | 59.76% |
| 698 | NNTRLWVYCGNGTPNELGGA | ag85B | 54.88% |
| 706 | QDAYNAAGGHNAVFNFPPNG | ag85B | 60.37% |
| 710 | DIKVQFQSGGNNSPAVYLLD | ag85B | 60.98% |
| 711 | NNSPAVYLLDGLRAQDDYNG | ag85B | 62.80% |
| 712 | SWEYWGAQLNAMKGDLQSSL | ag85B | 57.93% |
| 713 | DDYNGWDINTPAFEWYYQ | ag85B | 57.93% |
| 714 | WYSPACGKAGCQTYKWET | ag85B | 57.32% |
| 717 | SSDPAWERNDPTQQIPKLVA | ag85B | 58.54% |
| 718 | EYWGAQLNAMKGDLQSSLGA | ag85B | 56.71% |
| 719 | LTSELPQWLSANRAVKPTGS | ag85B | 58.54% |
| 720 | PSLIGLAMGDAGGYKAADMW | ag85B | 60.98% |
| 721 | NGTPNELGGANIPAEFLENF | ag85B | 57.93% |
| 722 | LIGLAMGDAGGYKAADMWGP | ag85B | 62.20% |
| 723 | SAMILAAYHPQQFIYAGSLS | ag85B | 58.54% |
| 724 | RNDPTQQIPKLVANNTRL | ag85B | 56.71% |
| 725 | HSWEYWGAQLNAMKGDLQ | ag85B | 57.93% |
| 728 | QQFIYAGSLSALLDPSQGMG | ag85B | 56.10% |
| 729 | WVYCGNGTPNELGGANIP | ag85B | 56.10% |
| 731 | YYQSGLSIVMPVGGQSSFYS | ag85B | 60.98% |
| 733 | KLVANNTRLWVYCGNGTP | ag85B | 53.05% |
| 736 | WGRRLMIGTAAAVVLPGLVG | ag85B | 37.80% |
| 738 | TPAFEWYYQSGLSIVMPV | ag85B | 60.98% |
| 741 | NELGGANIPAEFLENFVR | ag85B | 57.93% |
| 742 | GGQSSFYSDWYSPACGKA | ag85B | 62.20% |
| 743 | GGYKAADMWGPSSDPAWE | ag85B | 59.76% |
| 747 | ANRAVKPTGSAAIGLSMAGS | ag85B | 59.76% |
| 749 | GRDIKVQFQSGGNNSPAV | ag85B | 60.98% |
| 753 | ILAAYHPQQFIYAGSLSA | ag85B | 57.32% |
| 754 | FLTSELPQWLSANRAVKP | ag85B | 55.49% |
| 755 | SGGNNSPAVYLLDGLRAQ | ag85B | 62.20% |
| 757 | AAYHPQQFIYAGSLSALL | ag85B | 57.32% |
| 758 | FIYAGSLSALLDPSQGMG | ag85B | 56.71% |
| 759 | SGLSIVMPVGGQSSFYSD | ag85B | 62.20% |
| 761 | FPPNGTHSWEYWGAQ | ag85B | 61.59% |
| 768 | YAGSLSALLDPSQGMGPS | ag85B | 57.32% |
| 774 | PNGTHSWEYWGAQLN | ag85B | 61.59% |
| 782 | LSANRAVKPTGSAAIGLS | ag85B | 59.15% |
| 784 | ADMWGPSSDPAWERN | ag85B | 61.59% |
| 786 | PAWERNDPTQQIPKL | ag85B | 60.98% |
| 789 | QIPKLVANNTRLWVY | ag85B | 54.27% |
| 793 | PAEFLENFVRSSNLK | ag85B | 61.59% |
| 798 | SSFYSDWYSPACGKA | ag85B | 62.20% |
| 800 | AADMWGPSSDPAWER | ag85B | 60.37% |
| 805 | RKIRAWGRRLMIGTA | ag85B | 39.02% |
| 809 | KWETFLTSELPQWLS | ag85B | 53.66% |
| 814 | LPQWLSANRAVKPTG | ag85B | 60.98% |
| 816 | AAGGHNAVFNFPPNG | ag85B | 61.59% |
| 821 | TGSAAIGLSMAGSSAMIL | ag85B | 60.37% |
| 823 | PQWLSANRAVKPTGS | ag85B | 60.98% |
| 827 | FQSGGNNSPAVYLLD | ag85B | 62.20% |
| 835 | WETFLTSELPQWLSA | ag85B | 53.66% |
| 836 | HPQQFIYAGSLSALL | ag85B | 57.32% |
| 837 | LAMGDAGGYKAADMW | ag85B | 62.20% |
| 839 | SMGRDIKVQFQSGGN | ag85B | 58.54% |
| 852 | LSIVMPVGGQSSFYS | ag85B | 62.20% |
| 854 | SQGMGPSLIGLAMGD | ag85B | 62.80% |
| 858 | PAVSQFNARTADGINYRVLWQAAGP | mpt63 | 76.67% |
| 859 | YRVLWQAAGPDTISGATIPQGEQST | mpt63 | 70.00% |
| 860 | YPITGKLGSELTMTDTVGQVVLGWK | mpt63 | 96.67% |
| 861 | ATIPQGEQSTGKIYFDVTGPSPTIV | mpt63 | 73.33% |
| 862 | DVTGPSPTIVAMNNGMEDLLIWEP | mpt63 | 83.33% |
| 863 | TVGQVVLGWKVSDLKSSTAVIPGYP | mpt63 | 90.00% |
| 864 | WEATATVNAIRGSVTPAVSQFNART | mpt63 | 76.67% |
| 865 | SSTAVIPGYPVAGQVWEATATVNAI | mpt63 | 83.33% |
| 866 | MAAIATFAAPVALAAYPITGKLGSE | mpt63 | 86.67% |
| 867 | IRGSVTPAVSQFNARTADGI | mpt63 | 80.00% |
| 868 | VSDLKSSTAVIPGYPVAGQV | mpt63 | 86.67% |
| 880 | IYFDVTGPSPTIVAM | mpt63 | 86.67% |
| 887 | VALAAYPITGKLGSE | mpt63 | 93.33% |
| 892 | TFAAPVALAAYPITG | mpt63 | 86.67% |
| 897 | AMNNGMEDL | mpt63 | 90.00% |
| 898 | AIRGSVTPAV | mpt63 | 83.33% |
| 899 | VLGWKVSDL | mpt63 | 96.67% |
| 900 | AAYPITGKL | mpt63 | 96.67% |
| 901 | QVWEATATV | mpt63 | 100.00% |
| 902 | TMTDTVGQV | mpt63 | 100.00% |
| 903 | TMIKTAVAVV | mpt63 | 96.67% |
| 905 | ATFAAPVAL | mpt63 | 90.00% |
| 906 | AIATFAAPV | mpt63 | 90.00% |
| 911 | HPTTTYKAFDWDQAYRKPIT | mpt64 | 100.00% |
| 912 | WDQAYRKPITYDTLWQADTD | mpt64 | 88.24% |
| 913 | APKTYCEELKGTDTGQACQI | mpt64 | 90.20% |
| 914 | NDGVIFFFNPGELLPEAAGP | mpt64 | 86.27% |
| 915 | GTDTGQACQIQMSDPAYNIN | mpt64 | 90.20% |
| 916 | LKVYQNAGGTHPTTTYKAFD | mpt64 | 96.08% |
| 917 | CQIQMSDPAYNINISLPSYY | mpt64 | 92.16% |
| 918 | MRIKIFMLVTAVVLLCCSGV | mpt64 | 92.16% |
| 919 | GLDPVNYQNFAVTNDGVIFF | mpt64 | 70.59% |
| 920 | YDTLWQADTDPLPVVFPIVQ | mpt64 | 76.47% |
| 921 | PVNYQNFAVTNDGVIFFFNP | mpt64 | 74.51% |
| 922 | QMSDPAYNINISLPSYYPDQ | mpt64 | 94.12% |
| 923 | PDQKSLENYIAQTRDKFLSA | mpt64 | 92.16% |
| 925 | IKIFMLVTAVVLLCCSGVAT | mpt64 | 94.12% |
| 926 | IPPRGTQAVVLKVYQNAGGT | mpt64 | 94.12% |
| 927 | ISLPSYYPDQKSLENYIAQT | mpt64 | 94.12% |
| 928 | DTDPLPVVFPIVQGELSKQT | mpt64 | 84.31% |
| 929 | AIPPRGTQAVVLKVYQNAGG | mpt64 | 96.08% |
| 930 | AVVLLCCSGVATAAPKTYCE | mpt64 | 94.12% |
| 931 | VSIAPNAGLDPVNYQNFAVT | mpt64 | 76.47% |
| 932 | GELSKQTGQQVSIAPNAGLD | mpt64 | 90.20% |
| 933 | PLPVVFPIVQGELSKQTGQQ | mpt64 | 88.24% |
| 934 | YNINISLPSYYPDQKSLENY | mpt64 | 94.12% |
| 936 | STPREAPYELNITSATYQSA | mpt64 | 86.27% |
| 937 | GELLPEAAGPTQVLVPRSAI | mpt64 | 90.20% |
| 938 | NITSATYQSAIPPRGTQAVV | mpt64 | 94.12% |
| 940 | RDKFLSAATSSTPREAPYEL | mpt64 | 86.27% |
| 947 | TQVLVPRSAIDSMLA | mpt64 | 88.24% |
| 954 | FAVTNDGVI | mpt64 | 84.31% |
| 960 | GTHPTTTYK | mpt64 | 96.08% |
| 964 | MATTLPVQRHPRSLFPEFSE | hspX | 78.26% |
| 965 | RTEQKDFDGRSEFAYGSFVR | hspX | 91.30% |
| 966 | LFAAFPSFAGLRPTFDTRLM | hspX | 100.00% |
| 968 | YEVRAELPGVDPDKDVDIMV | hspX | 78.26% |
| 969 | SEFAYGSFVRTVSLPVGADE | hspX | 95.65% |
| 971 | RLEDEMKEGRYEVRAELPGV | hspX | 69.57% |
| 972 | TVSLPVGADEDDIKATYDKG | hspX | 95.65% |
| 973 | DPDKDVDIMVRDGQLTIKAE | hspX | 91.30% |
| 974 | PRSLFPEFSELFAAFPSFAG | hspX | 100.00% |
| 975 | RSLFPEFSELFAAFPSFAGL | hspX | 100.00% |
| 977 | ILTVSVAVSEGKPTEKHIQI | hspX | 78.26% |
| 978 | DDIKATYDKGILTVSVAVSE | hspX | 86.96% |
| 982 | PVQRHPRSLFPEFSE | hspX | 86.96% |
| 984 | PSFAGLRPTFDTRLM | hspX | 100.00% |
| 988 | DFDGRSEFAYGSFVR | hspX | 91.30% |
| 989 | KDFDGRSEFAYGSFV | hspX | 91.30% |
| 990 | FDGRSEFAYGSFVRT | hspX | 95.65% |
| 991 | MKEGRYEVRAELPGV | hspX | 78.26% |
| 992 | DGRSEFAYGSFVRTV | hspX | 95.65% |
| 993 | AYGSFVRTVSLPVGA | hspX | 95.65% |
| 994 | YGSFVRTVSLPVGAD | hspX | 95.65% |
| 998 | TIKAERTEQKDFDGR | hspX | 95.65% |
| 1000 | GRSEFAYGSFVRTVS | hspX | 95.65% |
| 1004 | FVRTVSLPVGADEDD | hspX | 95.65% |
| 1005 | RSEFAYGSFVRTVSL | hspX | 95.65% |
| 1006 | GSFVRTVSLPVGADE | hspX | 95.65% |
| 1007 | ELPGVDPDKDVDIMV | hspX | 86.96% |
| 1008 | PEFSELFAAFPSFAG | hspX | 100.00% |
| 1009 | VDIMVRDGQLTIKAE | hspX | 100.00% |
| 1010 | EGKPTEKHIQIRSTN | hspX | 78.26% |
| 1012 | DTRLMRLEDEMKEGR | hspX | 82.61% |
| 1015 | VAVSEGKPTEKHIQI | hspX | 78.26% |
| 1017 | GKPTEKHIQIRSTN | hspX | 82.61% |
| 1020 | TYDKGILTVSVAVSE | hspX | 86.96% |
| 1022 | VGADEDDIKATYDKG | hspX | 95.65% |

- **One hundred and fifty-two epitopes with a conservancy score > 95%**

| Header | Peptide | Relevant protein | Conservancy | Ag Score | Ag/non-Ag |
| --- | --- | --- | --- | --- | --- |
| 31 | VLMGGVPGVE | ald | 96.83% | 0.5397 | Non-Ag |
| 39 | YASVEAANASPLQVA | RS03355 | 99.30% | 0.8161 | Non-Ag |
| 40 | FVQALTTAAASYASV | RS03355 | 100% | 0.1215 | Non-Ag |
| 48 | PTGPASVQGMSQDPVAVAASNNPEL | RS03490 | 96.43% | 0.5971 | Non-Ag |
| 51 | QTSPANVVGTRQTLQGASVTVTGQG | RS03490 | 96.43% | 1.189 | Ag |
| 59 | PTNAAFSKLPASTIDELKTN | RS03490 | 96.43% | 0.5192 | Non-Ag |
| 69 | ANATVYMIDSVLMPP | RS03490 | 100.00% | 0.1181 | Non-Ag |
| 79 | TVYMIDSVLMPPA | RS03490 | 100.00% | -0.0691 | Non-Ag |
| 80 | VNLVDTLNSGQYT | RS03490 | 96.43% | 0.0945 | Non-Ag |
| 81 | AAFSKLPASTIDELK | RS03490 | 96.43% | 0.2782 | Non-Ag |
| 84 | NAAFSKLPAST | RS03490 | 96.43% | 0.374 | Non-Ag |
| 131 | DWYQPACGKAGCQTYKWETF | ag85A | 98.11% | 1.0896 | Ag |
| 132 | THSWEYWGAQLNAMKPDLQR | ag85A | 98.11% | 0.6747 | Non-Ag |
| 133 | NGVFDFPDSGTHSWEYWGAQ | ag85A | 98.11% | -0.1209 | Non-Ag |
| 139 | QRNDPLLNVGKLIANNTRVW | ag85A | 96.23% | -0.6736 | Non-Ag |
| 141 | PVGGQSSFYSDWYQPACGKA | ag85A | 98.11% | 0.7002 | Non-Ag |
| 142 | GNNLPAKFLEGFVRTSNIKF | ag85A | 98.11% | 0.6896 | Non-Ag |
| 145 | GPKEDPAWQRNDPLLNVGKL | ag85A | 96.23% | -0.0784 | Non-Ag |
| 148 | PDSGTHSWEYWGAQLNAMK | ag85A | 96.23% | 0.6729 | Non-Ag |
| 152 | LAIYHPQQFVYAGAMSGLLD | ag85A | 98.11% | 0.0192 | Non-Ag |
| 156 | LGGNNLPAKFLEGFVRTSNI | ag85A | 98.11% | 0.4281 | Non-Ag |
| 157 | SALTLAIYHPQQFVYAGAMS | ag85A | 98.11% | 0.1281 | Non-Ag |
| 160 | DIKVQFQSGGANSPALYLLD | ag85A | 96.23% | 1.3288 | Ag |
| 162 | DPAWQRNDPLLNVGKLIAN | ag85A | 98.11% | -0.4166 | Non-Ag |
| 163 | GNGKPSDLGGNNLPAKFLEG | ag85A | 96.23% | 1.2838 | Ag |
| 164 | QQFVYAGAMSGLLDPSQAMG | ag85A | 96.23% | -0.1864 | Non-Ag |
| 175 | AVVGLSMAASSALTLAIYHP | ag85A | 98.11% | 0.7643 | Non-Ag |
| 176 | VFDFPDSGTHSWEYW | ag85A | 98.11% | 0.3294 | Non-Ag |
| 177 | ACGKAGCQTYKWETF | ag85A | 98.11% | 1.2434 | Ag |
| 178 | FYSDWYQPACGKAGC | ag85A | 98.11% | 1.1331 | Ag |
| 180 | AGCQTYKWETFLTSE | ag85A | 96.23% | 0.259 | Non-Ag |
| 183 | KEDPAWQRNDPLLNV | ag85A | 98.11% | 0.5082 | Non-Ag |
| 186 | DSGTHSWEYWGAQLN | ag85A | 98.11% | 0.7764 | Non-Ag |
| 188 | SWEYWGAQLNAMKPD | ag85A | 98.11% | 0.8406 | Non-Ag |
| 189 | GGQSSFYSDWYQPAC | ag85A | 98.11% | 0.4243 | Non-Ag |
| 191 | FLEGFVRTSNIKFQD | ag85A | 98.11% | 0.5403 | Non-Ag |
| 193 | LTLAIYHPQQFVYAG | ag85A | 100.00% | 0.0287 | Non-Ag |
| 194 | KWETFLTSELPGWLQ | ag85A | 96.23% | -0.4456 | Non-Ag |
| 196 | QTYKWETFLTSELPG | ag85A | 96.23% | 0.1357 | Non-Ag |
| 199 | VMPVGGQSSFYSDWY | ag85A | 96.23% | 0.1457 | Non-Ag |
| 201 | GGHNGVFDFPDSGTH | ag85A | 96.23% | 0.3359 | Non-Ag |
| 203 | WQRNDPLLNVGKLIA | ag85A | 100.00% | -0.7814 | Non-Ag |
| 204 | AYNAGGGHNGVFDFP | ag85A | 96.23% | 1.3298 | Ag |
| 205 | YHPQQFVYAGAMSGL | ag85A | 98.11% | 0.0201 | Non-Ag |
| 207 | AIYHPQQFVYAGAMS | ag85A | 98.11% | -0.2345 | Non-Ag |
| 210 | VVMPVGGQSSFYSDW | ag85A | 96.23% | -0.0204 | Non-Ag |
| 214 | PLLNVGKLIANNTRV | ag85A | 96.23% | -0.7717 | Non-Ag |
| 221 | ATAGAFSRPGLPVEY | ag85A | 96.23% | -0.0417 | Non-Ag |
| 223 | KVQFQSGGANSPALY | ag85A | 96.23% | 1.1385 | Ag |
| 227 | FVYAGAMSGLLDPSQ | ag85A | 96.23% | -0.2233 | Non-Ag |
| 228 | FQSGGANSPALYLLD | ag85A | 96.23% | 0.8437 | Non-Ag |
| 229 | GAQLNAMKPDLQRAL | ag85A | 96.23% | 0.7224 | Non-Ag |
| 233 | AMSGLLDPSQAMGPT | ag85A | 96.23% | -0.1115 | Non-Ag |
| 235 | MGRDIKVQFQSGGAN | ag85A | 98.11% | 1.5879 | Ag |
| 236 | PSMGRDIKVQFQSGG | ag85A | 98.11% | 1.5022 | Ag |
| 237 | LSVVMPVGGQSSFYS | ag85A | 96.23% | 0.2489 | Non-Ag |
| 240 | AMGPTLIGLAMGDAG | ag85A | 96.23% | 0.3533 | Non-Ag |
| 241 | SMAASSALTLAIYHP | ag85A | 98.11% | 0.8329 | Non-Ag |
| 242 | QSGLSVVMPVGGQSS | ag85A | 96.23% | 0.8807 | Non-Ag |
| 255 | EISTNIRQAGVQYSRADEEQ | esxB | 95.12% | 1.0216 | Ag |
| 258 | LDEISTNIRQAGVQYSRAD | esxB | 95.12% | 0.7119 | Non-Ag |
| 264 | ATLAQEAGNFERISGDLK | esxB | 95.12% | 0.3537 | Non-Ag |
| 268 | NIRQAGVQYSRADEEQQQ | esxB | 95.12% | 1.1735 | Ag |
| 275 | SRADEEQQQALSSQMGF | esxB | 95.12% | 1.0284 | Ag |
| 282 | TNIRQAGVQYSRADE | esxB | 95.12% | 0.8695 | Non-Ag |
| 284 | RADEEQQQALSSQMGF | esxB | 95.12% | 1.032 | Ag |
| 285 | DEISTNIRQAGVQYS | esxB | 95.12% | 0.9299 | Non-Ag |
| 288 | STNIRQAGVQYSRAD | esxB | 95.12% | 0.9949 | Non-Ag |
| 292 | ISTNIRQAGVQYSRA | esxB | 95.12% | 0.7725 | Non-Ag |
| 293 | ERISGDLKTQIDQVE | esxB | 90.24% | 0.8317 | Non-Ag |
| 296 | DAATLAQEAGNFERI | esxB | 95.12% | 0.1455 | Non-Ag |
| 299 | TDAATLAQEAGNFER | esxB | 97.56% | 0.2156 | Non-Ag |
| 302 | KTDAATLAQEAGNFE | esxB | 97.56% | 0.556 | Non-Ag |
| 305 | RQAGVQYSRADEEQQ | esxB | 95.12% | 1.0748 | Ag |
| 316 | ADEEQQQALSSQMGF | esxB | 95.12% | 0.8533 | Non-Ag |
| 337 | EEQQQALSSQMGF | esxB | 95.12% | 0.669 | Non-Ag |
| 343 | QYSRADEEQQ | esxB | 97.56% | 1.1832 | Ag |
| 344 | QQALSSQMGF | esxB | 95.12% | 0.5549 | Non-Ag |
| 354 | EMKTDAATL | esxB | 95.12% | 1.282 | Ag |
| 357 | VESTAGSL | esxB | 97.56% | 1.1878 | Ag |
| 361 | SAIQGNVTSIHSLLDEGKQSLTKLA | esxA | 100.00% | 0.2453 | Non-Ag |
| 365 | VTSIHSLLDEGKQSLTKLAAAWGG | esxA | 100.00% | -0.0907 | Non-Ag |
| 379 | NVTSIHSLLDEGKQSLTKLA | esxA | 100.00% | 0.0331 | Non-Ag |
| 390 | SIHSLLDEGKQSLTKLAA | esxA | 100.00% | -0.1019 | Non-Ag |
| 391 | AIQGNVTSIHSLLDEGK | esxA | 100.00% | 0.3036 | Non-Ag |
| 400 | IHSLLDEGKQSLTKLA | esxA | 100.00% | -0.214 | Non-Ag |
| 402 | LDEGKQSLTKLAAAWG | esxA | 100.00% | 0.0151 | Non-Ag |
| 404 | QGNVTSIHSLLDEGK | esxA | 100.00% | 0.2011 | Non-Ag |
| 405 | GNVTSIHSLLDEGKQ | esxA | 100.00% | 0.1435 | Non-Ag |
| 409 | IQGNVTSIHSLLDEG | esxA | 100.00% | 0.3992 | Non-Ag |
| 420 | TSIHSLLDEGKQSLT | esxA | 100.00% | 0.0325 | Non-Ag |
| 423 | HSLLDEGKQSLTKLA | esxA | 100.00% | -0.2553 | Non-Ag |
| 526 | LLDEGKQSL | esxA | 100.00% | -1.0186 | Non-Ag |
| 534 | GSIRSLARAVVHAANLGVGV | mycP1 | 99.34% | 0.1098 | Non-Ag |
| 535 | AQIIHRITATARHPG | mycP1 | 98.01% | -0.0834 | Non-Ag |
| 536 | APYNVRRLPPPVVEP | mycP1 | 96.03% | 0.6003 | Non-Ag |
| 537 | DDLVGAGVIDAVA | mycP1 | 97.35% | 0.3884 | Non-Ag |
| 542 | QNYTYKWETFLTREMPAWLQ | ag85C | 96.23% | 0.2548 | Non-Ag |
| 545 | GFLNPSEGWWPTLIGLAMND | ag85C | 96.23% | -0.3175 | Non-Ag |
| 548 | RDTYAADGGRNGVFNFPPNG | ag85C | 98.11% | 0.7851 | Non-Ag |
| 549 | SGGYNANSMWGPSSDPAWKR | ag85C | 98.11% | 0.4792 | Non-Ag |
| 551 | PVGGQSSFYTDWYQPSQSNG | ag85C | 96.23% | 0.409 | Non-Ag |
| 553 | FSRPGLPVEYLQVPSASMGR | ag85C | 98.11% | 0.1997 | Non-Ag |
| 555 | YYQSGLSVIMPVGGQSSFYT | ag85C | 98.11% | 0.2641 | Non-Ag |
| 560 | GQNYTYKWETFLTRE | ag85C | 98.11% | 0.3497 | Non-Ag |
| 562 | VYLLDGLRAQDDYNG | ag85C | 98.11% | 0.5788 | Non-Ag |
| 567 | QVPSASMGRDIKVQF | ag85C | 96.23% | 1.4152 | Ag |
| 586 | MVNKSRMMPAV | lpqL | 96.63% | -0.3676 | Non-Ag |
| 598 | VVHPAVVQANRVRTWLLAVSNVFGQ | RS13395 | 95.65% | 0.3458 | Non-Ag |
| 599 | GQSVTGYNNSVSVTS | RS13395 | 96.27% | 1.2471 | Ag |
| 600 | GIVALIALGILEHFD | ggtA | 96.92% | -0.0059 | Non-Ag |
| 604 | TTSNVSVAK | rpfA | 99.61% | 1.0643 | Ag |
| 610 | GGLVRTVHLPAPNVA | rpfB | 96.03% | 0.599 | Non-Ag |
| 611 | AGVQVHDADTIVLRR | rpfB | 96.83% | 0.6229 | Non-Ag |
| 612 | TVHLPAPNVAGLLSA | rpfB | 97.62% | 0.3258 | Non-Ag |
| 614 | TAMRVTTMK | rpfB | 95.24% | 0.6439 | Non-Ag |
| 633 | PGTRINQETVSLDANGVSGS | apa | 97.93% | 1.695 | Ag |
| 645 | AAMASASLVTVAVPATANAD | apa | 97.24% | 0.4595 | Non-Ag |
| 661 | RTVSLPVGA | hspX | 100.00% | 0.1165 | Non-Ag |
| 663 | AFPSFAGL | hspX | 100.00% | -0.4392 | Non-Ag |
| 664 | SLFPEFSEL | hspX | 100.00% | -0.3702 | Non-Ag |
| 668 | ARVIMRSAIG | ripC | 98.10% | 0.0074 | Non-Ag |
| 860 | YPITGKLGSELTMTDTVGQVVLGWK | mpt63 | 96.67% | 0.7957 | Non-Ag |
| 887 | VALAAYPITGKLGSE | mpt63 | 93.33% | 0.8576 | Non-Ag |
| 899 | VLGWKVSDL | mpt63 | 96.67% | 0.6467 | Non-Ag |
| 900 | AAYPITGKL | mpt63 | 96.67% | 0.1988 | Non-Ag |
| 901 | QVWEATATV | mpt63 | 100.00% | 0.0481 | Non-Ag |
| 902 | TMTDTVGQV | mpt63 | 100.00% | 0.7589 | Non-Ag |
| 903 | TMIKTAVAVV | mpt63 | 96.67% | -0.1244 | Non-Ag |
| 911 | HPTTTYKAFDWDQAYRKPIT | mpt64 | 100.00% | 0.004 | Non-Ag |
| 913 | APKTYCEELKGTDTGQACQI | mpt64 | 90.20% | 1.3385 | Ag |
| 916 | LKVYQNAGGTHPTTTYKAFD | mpt64 | 96.08% | 1.0021 | Ag |
| 929 | AIPPRGTQAVVLKVYQNAGG | mpt64 | 96.08% | 0.6497 | Non-Ag |
| 960 | GTHPTTTYK | mpt64 | 96.08% | 1.2579 | Ag |
| 966 | LFAAFPSFAGLRPTFDTRLM | hspX | 100.00% | 0.9493 | Non-Ag |
| 969 | SEFAYGSFVRTVSLPVGADE | hspX | 95.65% | 0.2215 | Non-Ag |
| 972 | TVSLPVGADEDDIKATYDKG | hspX | 95.65% | 0.8443 | Non-Ag |
| 974 | PRSLFPEFSELFAAFPSFAG | hspX | 100.00% | -0.0016 | Non-Ag |
| 975 | RSLFPEFSELFAAFPSFAGL | hspX | 100.00% | -0.1618 | Non-Ag |
| 984 | PSFAGLRPTFDTRLM | hspX | 100.00% | 1.1786 | Ag |
| 990 | FDGRSEFAYGSFVRT | hspX | 95.65% | 0.2681 | Non-Ag |
| 992 | DGRSEFAYGSFVRTV | hspX | 95.65% | 0.3817 | Non-Ag |
| 993 | AYGSFVRTVSLPVGA | hspX | 95.65% | -0.0165 | Non-Ag |
| 994 | YGSFVRTVSLPVGAD | hspX | 95.65% | 0.0425 | Non-Ag |
| 998 | TIKAERTEQKDFDGR | hspX | 95.65% | 1.7884 | Ag |
| 1000 | GRSEFAYGSFVRTVS | hspX | 95.65% | 0.1878 | Non-Ag |
| 1004 | FVRTVSLPVGADEDD | hspX | 95.65% | 0.1714 | Non-Ag |
| 1005 | RSEFAYGSFVRTVSL | hspX | 95.65% | 0.2046 | Non-Ag |
| 1006 | GSFVRTVSLPVGADE | hspX | 95.65% | 0.1939 | Non-Ag |
| 1008 | PEFSELFAAFPSFAG | hspX | 100.00% | 0.148 | Non-Ag |
| 1009 | VDIMVRDGQLTIKAE | hspX | 100.00% | 1.1226 | Ag |
| 1022 | VGADEDDIKATYDKG | hspX | 95.65% | 1.1029 | Ag |

- **Fourteen antigenic and non-allergenic epitopes**

| Header | Peptide | Relevant protein | Conservancy | Ag Score | Ag/non-Ag | Allergenicity |
| --- | --- | --- | --- | --- | --- | --- |
| 160 | DIKVQFQSGGANSPALYLLD | ag85A | 96.23% | 1.3288 | Ag | Non-Allergen |
| 163 | GNGKPSDLGGNNLPAKFLEG | ag85A | 96.23% | 1.2838 | Ag | Non-Allergen |
| 178 | FYSDWYQPACGKAGC | ag85A | 98.11% | 1.1331 | Ag | Non-Allergen |
| 204 | AYNAGGGHNGVFDFP | ag85A | 96.23% | 1.3298 | Ag | Non-Allergen |
| 223 | KVQFQSGGANSPALY | ag85A | 96.23% | 1.1385 | Ag | Non-Allergen |
| 275 | SRADEEQQQALSSQMGF | esxB | 95.12% | 1.0284 | Ag | Non-Allergen |
| 284 | RADEEQQQALSSQMGF | esxB | 95.12% | 1.032 | Ag | Non-Allergen |
| 343 | QYSRADEEQQ | esxB | 97.56% | 1.1832 | Ag | Non-Allergen |
| 354 | EMKTDAATL | esxB | 95.12% | 1.282 | Ag | Non-Allergen |
| 567 | QVPSASMGRDIKVQF | ag85C | 96.23% | 1.4152 | Ag | Non-Allergen |
| 599 | GQSVTGYNNSVSVTS | RS13395 | 96.27% | 1.2471 | Ag | Non-Allergen |
| 913 | APKTYCEELKGTDTGQACQI | mpt64 | 90.20% | 1.3385 | Ag | Non-Allergen |
| 998 | TIKAERTEQKDFDGR | hspX | 95.65% | 1.7884 | Ag | Non-Allergen |
| 1022 | VGADEDDIKATYDKG | hspX | 95.65% | 1.1029 | Ag | Non-Allergen |
