# Supplementary material for: Design of a multi-epitope vaccine against Mycobacterium tuberculosis using reverse vaccinology and immunoreactive peptides
Source: Genomics Inform. 2026 Jul 8;24:13. doi: 10.1186/s44342-026-00075-6 (PMC13348615; doi:10.1186/s44342-026-00075-6)
Supplement: Supplementary file 6 — Supplementary Material 6: Data 6. MHC I and MHC II epitopes of the MEV identified using NetCTL-1.2 and IEDB web tool. [file 44342_2026_75_MOESM6_ESM.docx]

**Supplementary Data 6.** MHC I and MHC II epitopes of the designed MEV.

**MHC I epitopes**

| **Allele** | **Epitope** | **Score** |
| --- | --- | --- |
| HLA-A1 supertype | YSDWYQPAC | 0.7885 |
| HLA-A2 Supertype | SVTGYNNSV | 0.8914 |
| HLA-A3 supertype | SVTSEAAAK | 0.8429 |
| HLA-B39 | AKDIEKEIL | 1.0947 |
| HLA-B58 | GANSPALYL | 0.8302 |
| HLA-B62 | LLDGPGPGF | 0.9797 |
| HLA-B7 | GPGPGGQSV | 1.0627 |
| HLA-B7 | GPGPGKVQF | 1.0366 |
| HLA-B7 | FPGPGPGKV | 0.9917 |
| HLA-B8 | EMKTDAATL | 0.9026 |

**MHC II epitopes**

| **Allele** | **Core_peptide** | **Epitope** | **Score** | **Rank** |
| --- | --- | --- | --- | --- |
| HLA-DRB1*01:01 | YRILASKIV | TGQKYRILASKIVDF | 0.988 | 0.01 |
| HLA-DRB1*11:01 | FNNLVKLEQ | SNNFNNLVKLEQSLG | 0.9797 | 0.01 |
| HLA-DRB4*01:01 | IEKEILDLA | AKDIEKEILDLAAAT | 0.941 | 0.04 |
| HLA-DRB1*08:02 | YVLVMKANS | AGQYVLVMKANSSYS | 0.9382 | 0.02 |
| HLA-DRB1*04:01 | YNNSVSVTS | VTGYNNSVSVTSEAA | 0.8506 | 0.37 |
| HLA-DRB1*03:01 | MGRDIKVQF | SASMGRDIKVQFGPG | 0.8456 | 0.58 |
| HLA-DRB1*15:01 | VTGYNNSVS | GQSVTGYNNSVSVTS | 0.8179 | 0.5 |
| HLA-DRB1*01:01 | VLVMKANSS | GQYVLVMKANSSYSG | 0.7765 | 1.2 |
| HLA-DQA1*01:02/DQB1*06:02 | NNLVKLEQS | NNFNNLVKLEQSLGD | 0.7447 | 0.22 |
| HLA-DRB3*02:02 | YSNNFNNLV | FNIYSNNFNNLVKLE | 0.6812 | 0.4 |
| HLA-DRB1*04:01 | YLLDGPGPG | PALYLLDGPGPGFYS | 0.6357 | 1.8 |
| HLA-DQA1*05:01/DQB1*03:01 | YNAGGGHNG | PGAYNAGGGHNGVFD | 0.6161 | 1.3 |
| HLA-DQA1*01:02/DQB1*06:02 | NSVSVTSEA | GYNNSVSVTSEAAAK | 0.5809 | 0.87 |
| HLA-DRB3*02:02 | LNSNPAGNL | LTDALNSNPAGNLYD | 0.5516 | 0.78 |
| HLA-DPA1*03:01/DPB1*04:02 | IYSNNFNNL | VDFNIYSNNFNNLVK | 0.4933 | 0.18 |
| HLA-DRB3*01:01 | MKTDAATLG | PGEMKTDAATLGPGP | 0.4903 | 0.9 |
| HLA-DRB4*01:01 | VKDHYVDIS | GDGVKDHYVDISLDA | 0.4631 | 0.85 |
| HLA-DQA1*01:02/DQB1*06:02 | TDAATLGPG | EMKTDAATLGPGPGQ | 0.4618 | 1.8 |
| HLA-DRB3*02:02 | YSGNYPYSI | NSSYSGNYPYSILFQ | 0.4226 | 1.2 |
| HLA-DPA1*01:03/DPB1*04:01 | FYSDWYQPA | GPGFYSDWYQPACGK | 0.4164 | 0.54 |
| HLA-DRB3*01:01 | ISLDAGQYV | HYVDISLDAGQYVLV | 0.405 | 1.3 |
| HLA-DRB3*02:02 | MKANSSYSG | VLVMKANSSYSGNYP | 0.3128 | 1.8 |
| HLA-DPA1*02:01/DPB1*14:01 | KYRILASKI | TGQKYRILASKIVDF | 0.1493 | 1.7 |
| HLA-DQA1*03:01/DQB1*03:02 | DHYVDISLD | GVKDHYVDISLDAGQ | 0.0324 | 0.37 |
| HLA-DQA1*03:01/DQB1*03:02 | VTSEAAAKD | SVSVTSEAAAKDIEK | 0.0324 | 0.37 |
| HLA-DQA1*03:01/DQB1*03:02 | YVDISLDAG | VKDHYVDISLDAGQY | 0.0259 | 0.62 |
| HLA-DQA1*01:01/DQB1*05:01 | KDHYVDISL | DGVKDHYVDISLDAG | 0.0251 | 1.7 |
| HLA-DQA1*01:01/DQB1*05:01 | IVDFNIYSN | ASKIVDFNIYSNNFN | 0.0228 | 2 |
| HLA-DQA1*01:01/DQB1*05:01 | HNGVFDFPG | GGGHNGVFDFPGPGP | 0.0226 | 2 |
| HLA-DQA1*03:01/DQB1*03:02 | SVSVTSEAA | YNNSVSVTSEAAAKD | 0.0215 | 1.1 |
